# The prognostic value and clinical significance of mitophagy-related genes in hepatocellular carcinoma

**DOI:** 10.3389/fgene.2022.917584

**Published:** 2022-08-05

**Authors:** Wei Xu, Dongxu Zhao, Xiaowei Huang, Man Zhang, Minyue Yin, Lu Liu, Hongyu Wu, Zhen Weng, Chunfang Xu

**Affiliations:** ^1^ Department of Gastroenterology, The First Affiliated Hospital of Soochow University, Suzhou, China; ^2^ Department of Interventional Radiology, The First Affiliated Hospital of Soochow University, Suzhou, China; ^3^ Department of General Surgery, The First Affiliated Hospital of Soochow University, Suzhou, China; ^4^ Department of Emergency Medicine, The Affiliated Hospital of Xuzhou Medical University, Xuzhou, China; ^5^ Cyrus Tang Hematology Center and Ministry of Education Engineering Center of Hematological Disease, and the Collaborative Innovation Center of Hematology, Soochow University, Suzhou, China

**Keywords:** hepatocellular carcinoma, mitophagy, prognosis, tumor microenvironment, chemotherapy, targeted therapy, immune checkpoint

## Abstract

**Background:** Mitophagy has been found to play a significant part in the cancer process in a growing number of studies in recent years. However, there is still a lack of study on mitophagy-related genes’ (MRGs) prognostic potential and clinical significance in hepatocellular carcinoma (HCC).

**Methods:** We employed bioinformatics and statistical knowledge to examine the transcriptome data of HCC patients in the TCGA and GEO databases, with the goal of constructing a multigene predictive model. Then, we separated the patients into high- and low-risk groups based on the score. The model’s dependability was determined using principal components analysis (PCA), survival analysis, independent prognostic analysis, and receiver operating characteristic (ROC) analysis. Following that, we examined the clinical correlations, pharmacological treatment sensitivity, immune checkpoint expression, and immunological correlations between patients in high and low risk groups. Finally, we evaluated the variations in gene expression between high- and low-risk groups and further analyzed the network core genes using protein-protein interaction network analysis.

**Results:** Prognostic models were built using eight genes (*OPTN*, *ATG12*, *CSNK2A2*, *MFN1*, *PGAM5*, *SQSTM1*, *TOMM22*, *TOMM5*). During validation, the prognostic model demonstrated high reliability, indicating that it could accurately predict the prognosis of HCC patients. Additionally, we discovered that typical HCC treatment medicines had varying impacts on patients classified as high or low risk, and that individuals classified as high risk are more likely to fail immunotherapy. Additionally, the high-risk group expressed more immunological checkpoints. The immunological status of patients in different risk categories varies as well, and patients with a high-risk score have a diminished ability to fight cancer. Finally, PPI analysis identified ten related genes with potential for research.

**Conclusion:** Our prognostic model had good and reliable predictive ability, as well as clinical diagnosis and treatment guiding significance. Eight prognostic MRGs and ten network core genes merited further investigation.

## Introduction

Primary liver cancer is the second most frequently occurring malignant tumor disease on a global scale, second only to lung cancer in terms of incidence. The two primary types of liver cancer are hepatocellular carcinoma (HCC) and intrahepatic cholangiocarcinoma (ICC), with hepatocellular carcinoma being the more common pathological type (comprising 75%–85% of cases) (Wallace et al., 2015; Sayiner et al., 2019; Sung et al., 2021). Any factor that contributes to hepatic cirrhosis is a significant risk factor for hepatocellular cancer. Alcoholism and hepatitis C virus infection are the most common in European and American countries, while hepatitis B virus infection is the most common in Asian countries (Massarweh and El-Serag, 2017; Carcinoma, 2021; Shi et al., 2021). Additionally, aflatoxin, non-alcoholic fatty liver disease, polycyclic aromatic hydrocarbons in cigarettes, and medication poisoning (e.g., aristolochic acid) have been established as significant risk factors for HCC (Nixon, 1990; Chen et al., 2002; Degasperi and Colombo, 2016; Lu et al., 2020). Surgical resection is the primary therapeutic option for HCC. Chemotherapy, radiation, immunotherapy, and tranarterial embolization (TAE) are other treatment options (Adam, 2003; Aitken and Hawkins, 2014; Xu et al., 2018; van Rosmalen et al., 2019). Despite in-depth investigations, the prognosis of HCC patients remains dismal and unpredictable due to the high heterogeneity of the disease (Li and Wang, 2016; Ma et al., 2019; Losic et al., 2020). The Barcelona Clinical Staging System (BCLC), which evaluates the prognosis of patients by examining characteristics such as tumor size, number, and physical state, is currently the most widely used method for predicting the prognosis of HCC patients (Mauro and Forner, 2022; Reig et al., 2022). Furthermore, several biomarkers have been gradually discovered to be effective in determining the prognosis of HCC patients (Gao et al., 2020; Nault and Villanueva, 2021). However, long-term clinical experience has shown that they are not always correct. As a result, finding new prognostic prediction methods is still an important task in HCC research at the moment, not only to better understand the potential adverse outcomes of patients so that unnecessary overtreatment can be avoided, but also to generate new ideas for future research on HCC mechanisms.

Autophagy is a multistage process that occurs in eukaryotic cells. Its primary function is to transport intracellular components to lysosomes for degradation (Parzych and Klionsky, 2014; Klionsky et al., 2021). This function is critical for energy metabolism, stress defense, differentiation, and development (Mizushima et al., 2008; Kim and Lee, 2014; Levine and Kroemer, 2019). We now have adequate evidence to demonstrate that autophagy is involved in a variety of human illnesses, most notably cancer (Scrivo et al., 2018; Poillet-Perez and White, 2019; Wen et al., 2021). Mitophagy is an autophagic process that occurs in mitochondria, as well as a process by which mitochondria clear defective mitochondrial proteins (Montava-Garriga and Ganley, 2020; Belousov et al., 2021). Mitochondrial DNA and protein are susceptible to mutation and folding mistakes as a result of being exposed to high quantities of reactive oxygen species, resulting in alterations in mitochondrial function. Thus, mitochondrial autophagy is a mechanism by which mitochondria regulate their own amount and function (Ashrafi and Schwarz, 2013; Yan et al., 2019). On the basis of previous research on autophagy, researchers sought to determine whether mitophagy played a similar role in human diseases, and the study’s findings confirmed their hypothesis that mitophagy did play a distinct role in the occurrence of cardiovascular system diseases, nervous system diseases, and tumor diseases (Bravo-San Pedro et al., 2017; Lou et al., 2020; Panigrahi et al., 2020). Thus, we have grounds to wonder whether hepatocellular cancer likewise exhibits an aberrant increase in mitochondrial autophagy. Is mitophagy related to the prognosis of HCC? Given the scarcity of previous research on mitophagy and hepatocellular carcinoma, we carried out this study to see how mitophagy-related genes were expressed and how they affected prognosis in HCC patients.

Our study collected transcriptome and clinical data from HCC patients using public databases such as TCGA and GEO and conducted in-depth and extensive analysis to develop a risk model for HCC prognosis based on mitophagy-related genes. To demonstrate that our model is scientific, dependable, and stable, we validated it using a number of validation approaches. Additionally, we undertook a more thorough analysis of this prognostic model to determine its link to clinical risk variables and tumor-specific immune function. In conclusion, our study was trustworthy, has clinical relevance, and could provide a new direction for future research on HCC and mitophagy.

## Materials and methods

### Acquisition of research data

The Cancer Genome Atlas (TCGA) and Gene Expression Omnibus (GEO) databases were used to obtain transcriptome and clinical data for all patients in this study. The TCGA data are from the TCGA platform hepatocellular carcinoma data (TCGA-LIHC), and the GEO data are from the GSE54236 microarray data. Additionally, we processed patient clinical data. For analysis, we extracted basic data (age, gender), survival data (survival time/month, survival status), and pathology data (grade, stage, and T, N, M stage) from patients in the TCGA cohort. In the GEO cohort, only survival data was selected for analysis (survival time and survival status). Finally, the patients in the TCGA database serve as a training set for developing the prognosis model, while the patients in the GEO database serve as a validation set for developing and validating the prognosis model.

We searched the Gene Set Enrichment Analysis (GSEA) database for all mitophagy-related genes (MRGs). Furthermore, we performed annotations on the final selected gene functions and biological processes using the online databases The Human Protein Atlas (HPA) and The Database for Annotation, Visualization and Integrated Discovery (DAVID).

### Construction of prognostic models

We used R software to analyze the data after we completed the data download and collation. We first used the “limma” package to compare the expression of MRGs in tumor and non-tumor patients in the TCGA and GEO cohorts, and the intersection was determined by the screening results of the two groups. We set the filter standard to FDR <0.75 (the log function calculation results based on the difference multiple of two were performed, and the positive and negative values represented the up-regulation or down-regulation of genes, and the value represented the ratio of the two groups of expression levels). The selected gene expression was then combined with clinical data, and we used the single factor COX regression analysis of ‘survival’ package to screen for prognostic related genes (the standard is that *p* value was less than 0.05 or the confidence interval of HR does not contain 1). The construction of the prognostic model consisted of a training set (TCGA cohort) and a validation set (GEO cohort). The prognostic genes co-expressed by TCGA and GEO were extracted. Finally, we used the “glmnet” package to plug the prognostic genes into the Least absolute shrinkage and selection operator (LASSO) regression to build the prognostic model, and the prognostic model genes were multiplied by the coefficient to get the risk score formula.

### The reliability of the prognostic model was verified by multiple methods

After developing a prognostic model, it is necessary to validate it in order to demonstrate its dependability in predicting prognosis. The prognostic models were verified in TCGA and GEO cohorts, respectively. For further analysis, we divide the TCGA and GEO cohorts into high- and low-risk groups using the risk score algorithm. Principal component analysis (PCA) is a method for reducing the dimension of a dataset by capturing the primary features and ignoring superfluous repeated elements. Two groups of patients were analyzed using PCA, and the model was validated using observation point separation or fusion. Subsequently, we analyzed the overall survival (OS) and progression-free survival (PFS) of the two groups of patients based on the product-limit method (KM method) to observe whether there were significant differences in survival between the two groups. Independent prognostic analysis is a method that uses single-factor and multi-factor COX regression analysis to determine whether the prognostic model is independent of various other clinical traits and to predict prognosis. The difference between univariate COX regression analysis and multivariate regression analysis is whether the interaction between risk score and other clinical case factors is considered. Receiver operating characteristic (ROC) curve can reflect the trend of sensitivity (FPR) and specificity (TPR) of prognostic models when selecting different thresholds, and the area under the curve is called Area under roc Curve (AUC). ROC curve and AUC play a qualitative and quantitative analysis on the prognosis model, respectively. Therefore, we finally use ROC and AUC to evaluate the predictive ability of the prognostic model. When AUC fluctuates in the range of 0.5-1, it is considered that the model has good prediction accuracy.

### Further exploration on prognostic model

Following validation of the prognostic model’s reliability, we further investigated its potential guiding relevance in clinical diagnosis and treatment, immunological infiltration, and so on.

To assess the prognosis model’s utility in clinical diagnosis and treatment, we first determined whether clinically relevant variables (such as age, gender, grading, staging, and T, N, M stage) of TCGA patients were significantly different across risk groups, and then created a boxplot of clinical variables with statistically significant differences. Then, we used the “pRRophetic” package to investigate the drug sensitivity of regularly used HCC medications, to observe their potential therapeutic effects in high and low-risk groups, in order to recognize chemotherapeutic agents with increased sensitivity to low-risk groups. Additionally, we evaluated the expression of immunological checkpoints in patients belonging to various risk groups. Ultimately, we investigated the immune treatment of patients with varying risk groups to determine whether there is a higher probability of immune escape during immunotherapy in patients with a high-risk score compared to patients with a low-risk score, giving rise to the failure of immunotherapy.

In the end, the gene expression levels between the high and low-risk groups were analyzed again, and the differentially expressed genes were enriched and analyzed for protein interaction. The String online site was used for PPI analysis, and then the ' Cytoscape ' software was used to screen out the hub genes in the network and visualize them. We chose the top 10 core genes and conducted single-gene clinical correlation, single-gene survival correlation, and single-gene immunological correlation analyses. This is a continuation of the model’s study and examination of the prognostic model’s function. This model can be used to identify additional genes having a high value in HCC.

### Web address of online website

TCGA: https://www.cancer.gov/about-nci/organization/ccg/research/structural-genomics/tcga

GEO: https://www.ncbi.nlm.nih.gov/geo

HPA: https://www.proteinatlas.org

DAVID: https://david.ncifcrf.gov

STRING: https://cn.string-db.org

#### Statistical analysis

All statistical results were statistically significant when *p* < 0.05.

## Results

### The data of MRGs in patients from TCGA and GEO cohorts

**FIGURE 1:**
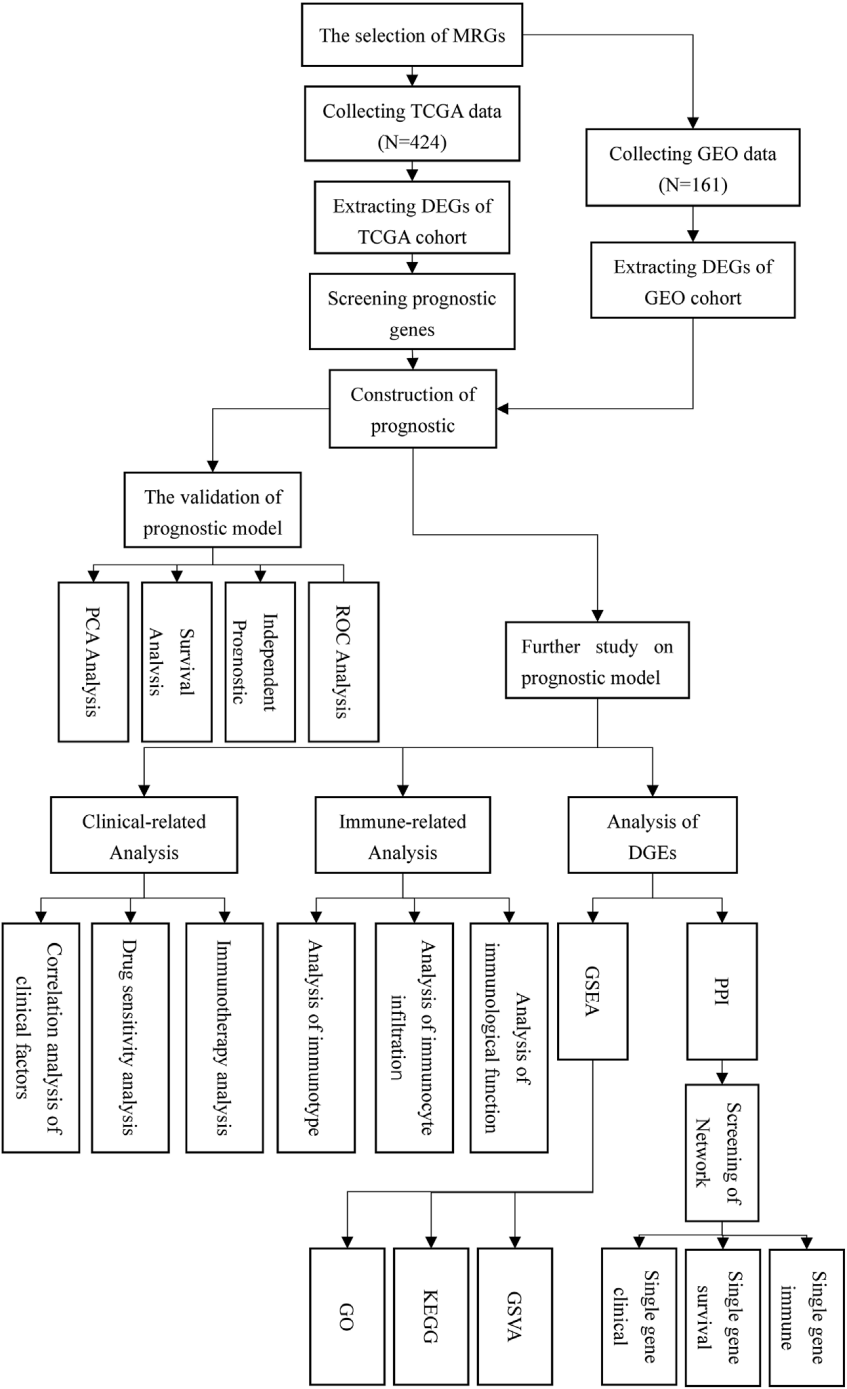
flow chart.

Figure 1 depicted the flow chart for this study. Detailed scripts could be seen in the Supplementary Data Sheet S2. Four hundred and twenty-four transcriptome data were obtained by TCGA in total. (FPKM adjusted), including 374 HCC tissue data and 50 normal tissue data. In GEO, one hundred and sixty-one HCC and normal tissue data were gathered from the same patient in total. Preliminary processing of patient data included gene annotation, deletion of missing data, and survival data shorter than 30 days. Additionally, to analyze gene mutations in tumor tissues, we downloaded the HCC gene mutation data from TCGA and selected the mutation data processed using the “varscan” software.

The GSEA database was searched for the term “mitophagy” and four gene sets were obtained. After deleting duplicate results, a total of 34 mitophagy-related genes was identified (Table 1). For future study, the MRGs expression levels in patients from the TCGA and GEO cohorts were retrieved. All 34 MRGs could be extracted for the levels of expression in TCGA cohort, while the GEO cohort could extract for 27 corresponding genes.

**TABLE.1:** All mitophagy-related genes and the biological functions of their encoded proteins.

Gene	Protein	Biological process
*CDC37(73)*	Cell division cycle 37	Regulating cell cycle
*HDAC6(74)*	Histone deacetylase 6	Autophagy, Transcription, Transcription regulation
*HUWE1(75)*	HECT, UBA and WWE domain containing E3 ubiquitin protein ligase 1	Biological rhythms, Differentiation, DNA damage, DNA repair, Ubl conjugation pathway
*MFN2(76)*	Mitofusin 2	Apoptosis, Autophagy, Unfolded protein response
*OPTN(77)*	Optineurin	Autophagy, Host-virus interaction, Immunity, Innate immunity
*PINK1(78)*	PTEN induced kinase 1	Autophagy
*PRKN(78)*	Parkin RBR E3 ubiquitin protein ligase	Autophagy, Transcription, Transcription regulation, Ubl conjugation pathway
*TOMM7(79)*	Translocase of outer mitochondrial membrane 7	Protein transport, Transport
*VPS13C(80)*	Vacuolar protein sorting 13 homolog C	Mitochondrial respiration
*ATG12(81)*	Autophagy related 12	Autophagy, Host-virus interaction, Ubl conjugation pathway
*ATG5(82)*	Autophagy related 5	Apoptosis, Autophagy, Host-virus interaction, Immunity
*CSNK2A1*	Casein kinase 2 alpha 1	Apoptosis, Biological rhythms, Cell cycle, Transcription, Transcription regulation, Wnt signaling pathway
*CSNK2A2*	Casein kinase 2 alpha 2	Apoptosis, Cell cycle, Transcription, Transcription regulation, Wnt signaling pathway
*CSNK2B(83)*	Casein kinase 2 beta	Wnt signaling pathway
*FUNDC1(84)*	FUN14 domain containing 1	Autophagy
*MAP1LC3A(85)*	Microtubule associated protein 1 light chain 3 alpha	Autophagy, Ubl conjugation pathway
*MAP1LC3B(84)*	Microtubule associated protein 1 light chain 3 beta	Autophagy, Ubl conjugation pathway
*MFN1(86)*	Mitofusin 1	GTP-binding, Nucleotide-binding
*MTERF3*	Mitochondrial transcription termination factor 3	Ribosome biogenesis, Transcription, Transcription regulation
*PGAM5(87)*	PGAM family member 5, mitochondrial serine/threonine protein phosphatase	Necrosis
*RPS27A*	Ribosomal protein S27a	Ribosomal metabolism
*SQSTM1(88)*	Sequestosome 1	Apoptosis, Autophagy, Differentiation, Immunity
*SRC(89)*	SRC proto-oncogene, non-receptor tyrosine kinase	Cell adhesion, Cell cycle, Host-virus interaction, Immunity
*TOMM20(90)*	Translocase of outer mitochondrial membrane 20	Protein transport, Transport
*TOMM22(83)*	Translocase of outer mitochondrial membrane 22	Protein transport, Translocation, Transport
*TOMM40(91)*	Translocase of outer mitochondrial membrane 40	Ion transport, Protein transport, Transport
*TOMM5*	Translocase of outer mitochondrial membrane 5	Protein transport, Transport
*TOMM6*	Translocase of outer mitochondrial membrane 6	Protein transport, Transport
*TOMM70(91)*	Translocase of outer mitochondrial membrane 70	Host-virus interaction
*UBA52(92)*	Ubiquitin A-52 residue ribosomal protein fusion product 1	Ribosomal metabolism
*UBB*	Ubiquitin B	Ribosomal metabolism
*UBC(93)*	Ubiquitin C	Ribosomal metabolism
*ULK1(94)*	Unc-51 like autophagy activating kinase 1	Autophagy
*VDAC1(88)*	Voltage dependent anion channel 1	Apoptosis, Host-virus interaction, Ion transport, Transport

All genes have been proved to be closely involved in mitophagy-related pathways. Gilad Twig et al. (Twig et al., 2008) marked and tracked mitochondria through fusion and fission, and finally identified nine genes closely related to mitophagy: HDAC6, HUWE1, OPTN, CDC37, PRKN, TOMM7, VPS13C, PINK1, MFN2. ATG5, TOMM22, MAP1LC3A, MFN2, TOMM40, MAP1LC3B, RPS27A, ATG12, UBC, TOMM70, MTERF3, PINK1, SQSTM1, UBB, MFN1, TOMM20, TOMM5, PRKN, TOMM7, VDAC1, TOMM6, UBA52. SQSTM1 selectively removes damaged mitochondria through PINK1-PRKN pathway and subsequently participates in the process of mitophagy through lysosome catabolism (https://reactome.org/PathwayBrowser/#/R-HSA-5205685). ATG5, FUNDC1, CSNK2A2, CSNK2A1, MAP1LC3A, MAP1LC3B, ATG12, ULK1, SRC, CSNK2B, CSNK2B, CSNK2B, CSNK2B, CSNK2B, CSNK2B, CSNK2B, CSNK2B, PGAM5 associates cell differentiation signals and mitochondrial function markers with scaffold proteins by participating in receptor-mediated mitophagy pathway, thereby further recruiting other autophagy proteins to form autophagosomes. Mitochondria destruction and recovery (https://reactome.org/PathwayBrowser/#/R-HSA-8934903).

### Screening of DEGs and construction of prognostic model

**FIGURE 2:**
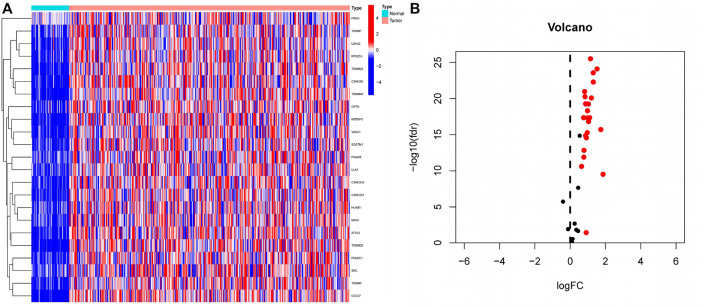
Extraction of differentially expressed mitophagy-related genes **(A)** A total of 23 genes related to mitophagy were differentially expressed between HCC tissues and normal tissues. In the heatmap, the front half of the transverse axis represented normal tissue, and the latter half represented tumor tissue. Red mean high gene expression, blue mean low gene expression; **(B)** Volcanic map of the expression of thirty-four MRGs in HCC tissues. The expression of red genes was up-regulated in HCC tissues, and the expression of black genes was not significantly changed in HCC tissues.

**FIGURE 3:**
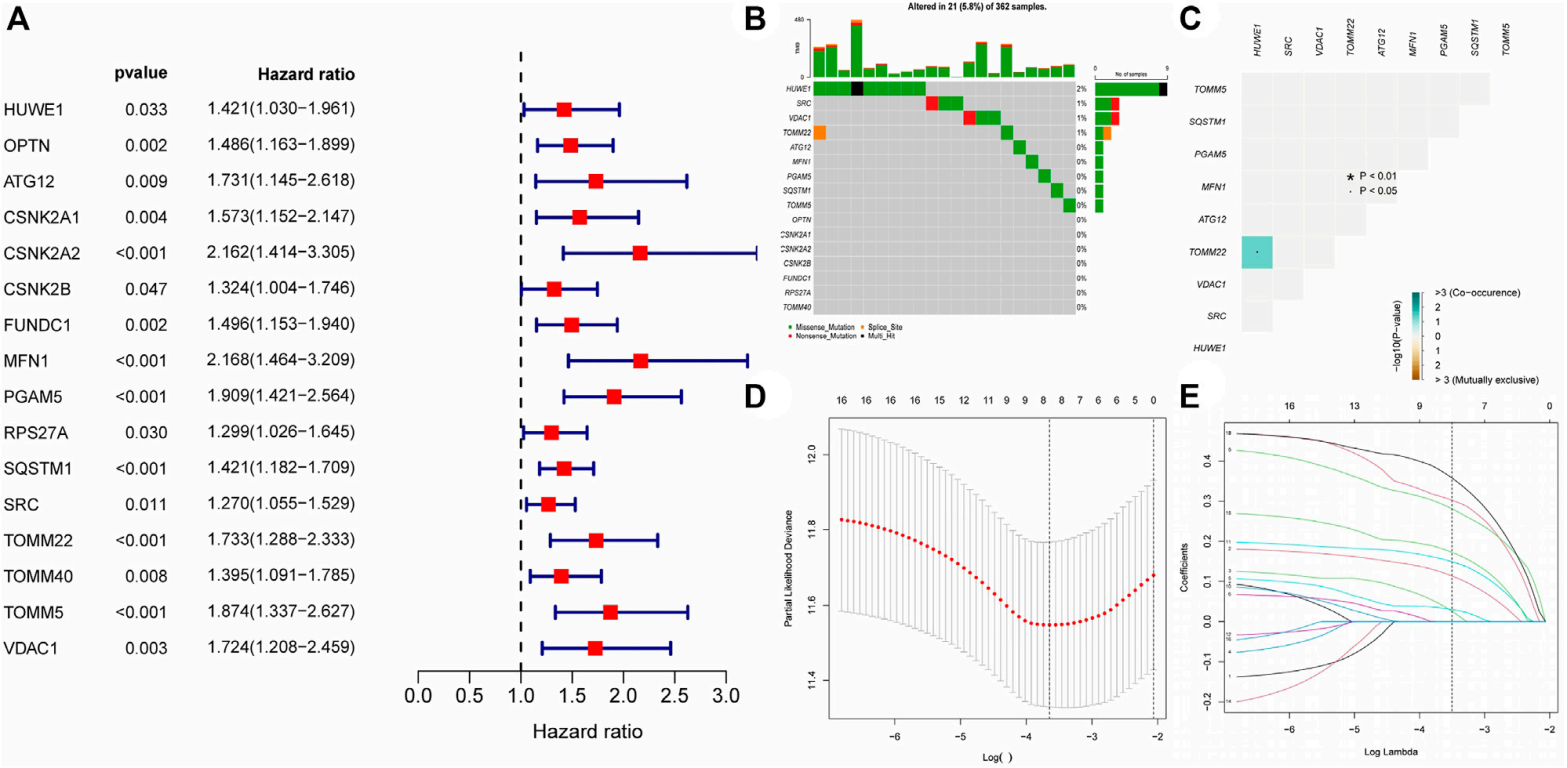
Gene mutation analysis and establishment of prognostic model **(A)** The result of Single factor COX regression The inclusion criteria were that the *p* value was less than 0.05 and the HR confidence interval did not include 1; **(B)** Waterfall map of gene mutation. **(C)** Co-mutation of 16 prognostic-related genes. Green represented that the mutation of one gene would promote the mutation of another gene. Brown indicated that one gene mutation inhibits another gene mutation; **(D–E)** The LASSON regression punished all variables. The coordinates at the lowest point of the red line in Figure D were the penalty values. A dotted line was drawn at the corresponding penalty value in Figure F, and all the independent variables that had great influence on the dependent variables were selected (each curve represents the change trajectory of each independent variable coefficient).

Differential expression analysis of 34 MRGs revealed that a total of 23 genes were differentially expressed across HCC and normal tissues in the TCGA cohort. The heatmap clearly demonstrated that these 23 genes were expressed at much greater levels in HCC tissues than in normal tissues (Figure 2A), and the volcano map demonstrated that these 23 genes were likewise up-regulated in HCC tissues (Figure 2B). Following that, we integrated the differential gene expression data with clinical data and utilized single-factor cox regression analysis to determine the link between differential genes and the prognosis of HCC patients. The result of univariate cox regression analysis demonstrated that 16 MRGs were positively connected with the prognosis of HCC and negatively correlated with the prognosis of HCC (Figure 3A). Following that, we examined the mutations in these 16 prognostic genes in tumor tissues. It is obvious that the mutation rates of these 16 genes in HCC tissues are modest; even HUWE1, which has the highest mutation rate, is just 2% (Figure 3B), and only *TOMM22* and HUWE1 show an indigenous co-mutation connection, implying that either of the two mutations will cause the other (Figure 3C).

**FIGURE 4:**
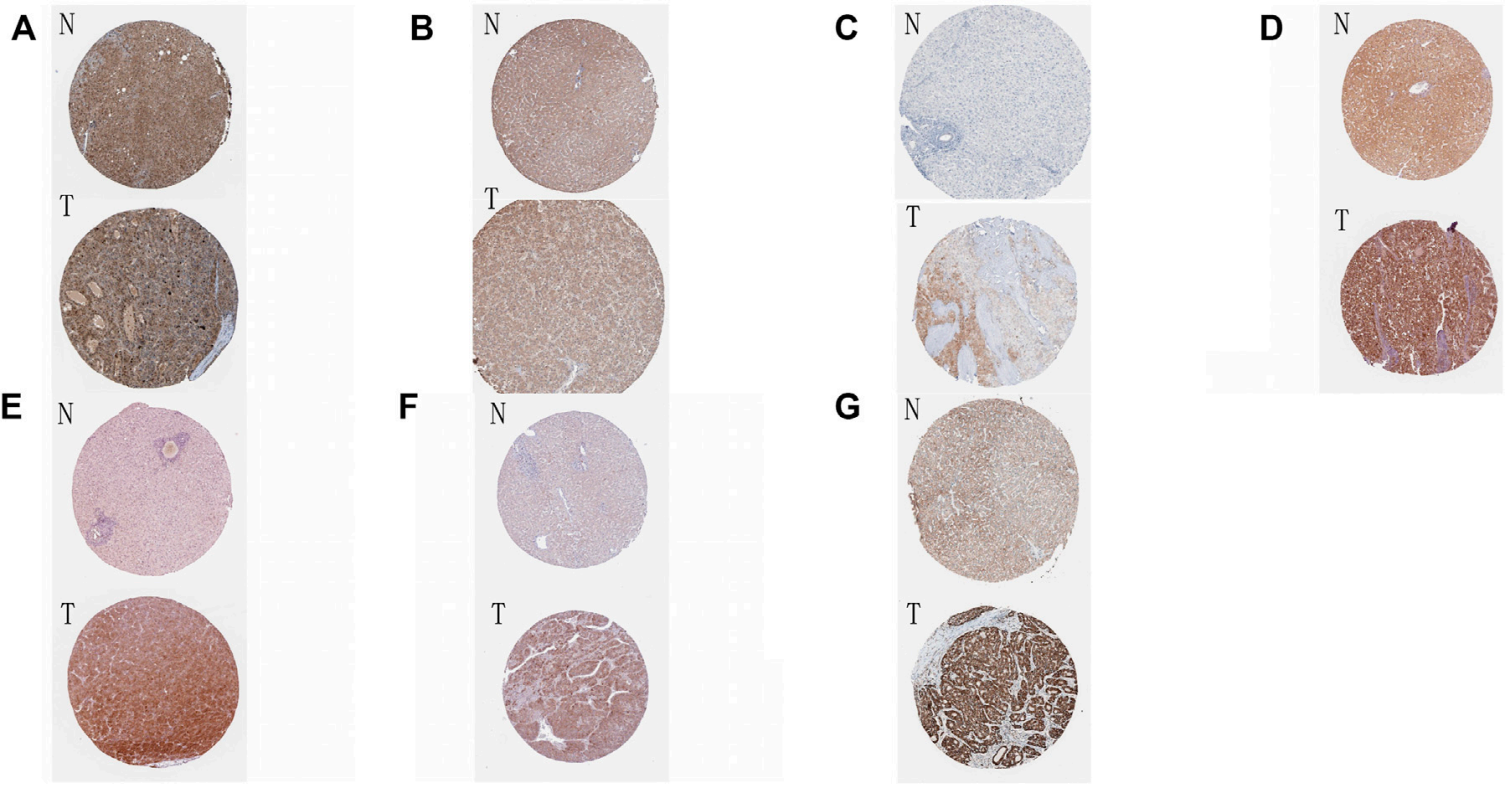
Immunohistochemical results of eight genes in tumor tissues and normal tissues **(A–G)** The immunohistochemical expression of eight genes in normal tissues and HCC tissues in HPA online database. N: normal tissue, T: HCC tissue. **(A)** *ATG*12; **(B)** *MFN1*; **(C)** *OPTN*; **(D)** *PGAM5*; **(E)** *SQSTM1*; **(F)** *TOMM5*; **(G)** *TOMM22*.

Finally, we incorporated these 16 genes into the LASSO regression analysis and finally obtained a prognostic model (Figures 3D,E). The prognostic model was constructed using a total of eight genes. Table 2 contains the gene name and LASSO coefficient. We examined the immunohistochemistry expression of seven genes (*CSNK2A2* was not identified) in normal human tissues and HCC tissues from the HPA database and discovered that the majority of genes were overexpressed in HCC tissues (Figures 4A–G). Thus, risk score formula was as followed:

$$\begin{array}{l} \text{Risk}\;\text{Score}=\mathrm{TOMM}22\;\text{expression}\times 0.373+MFN1\;\text{expression}\times 0.310\\ +PGAM5\;\text{expression}\times 0.292+TOMM5\;\text{expression}\times 0.181\\ +SQSTM1\;\text{expression}\times 0.156+OPTN\;\text{expression}\times 0.122\\ +ATG12\;\text{expression}\times 0.040+CSNK2A2\;\text{expression}\times 0.034 \end{array}$$

**TABLE.2:** Eight genes for prognostic model and their risk coefficient.

Gene	Coefficient
*TOMM22*	0.373,206
*MFN1*	0.310,108
*PGAM5*	0.291,848
*TOMM5*	0.180,964
*SQSTM1*	0.155,536
*OPTN*	0.122,111
*ATG12*	0.040225
*CSNK2A2*	0.033673

### Validation of prognostic models by various methods

**FIGURE 5:**
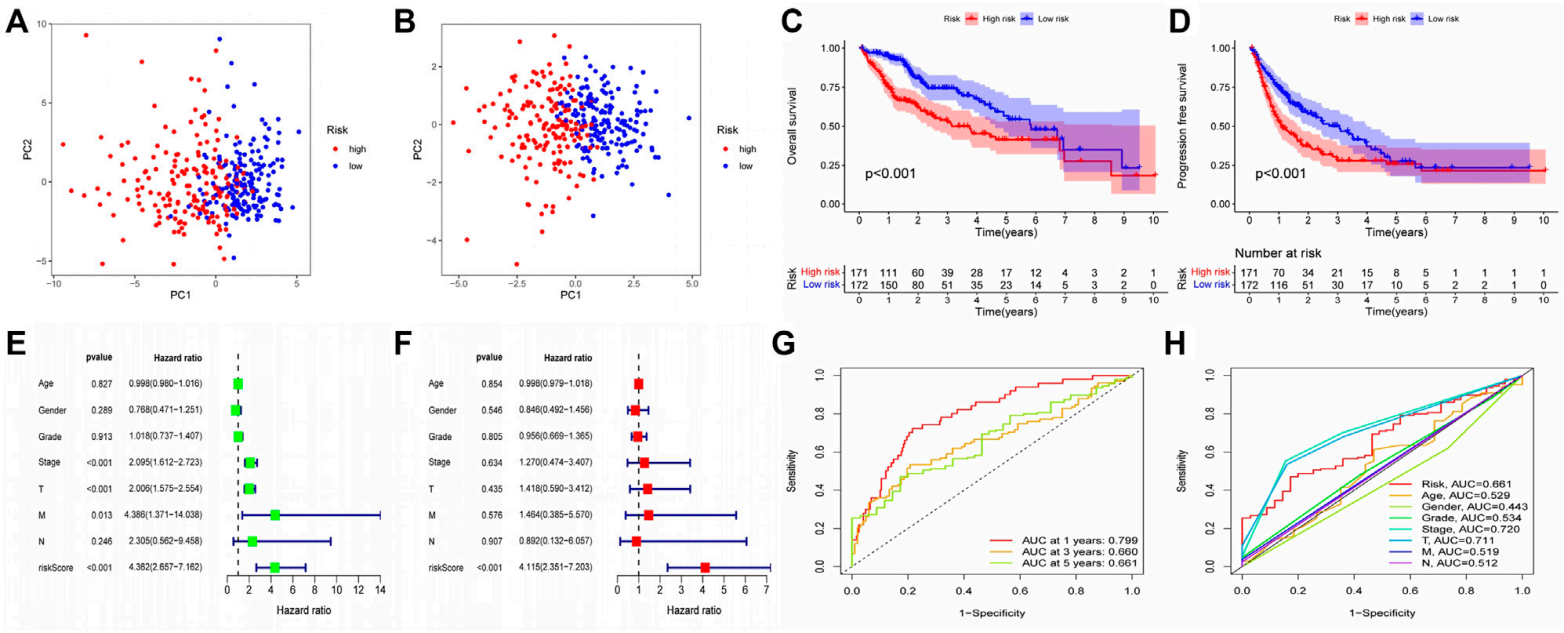
Validation of Prognostic Model Reliability by Several Methods **(A–B)** PCA analysis showed that patients with different risk scores could be well distinguished in the model. Blue points represented low risk group, and red points represented high risk score group; **(A)** training set (TCGA cohort). **(B)** validation set (GEO cohort) **(C–D)** Survival analysis based on Kaplan-Meier method was performed on the total survival time and progression-free survival time of patients with different risk scores. The horizontal axis under the image refers to the number of surviving patients in different years; **(C)** training set (TCGA cohort). **(D)** validation set (GEO cohort) **(E–F)** The prognostic model and common clinical features were analyzed by univariate or multivariate COX regression. HR value represents that the probability of death in the high-risk group per unit time was a multiple of that in the low-risk group. **(G)** The area under the ROC curve was used to evaluate the accuracy of the prognostic model. Red, yellow and green lines represent the 1 - year, 2 - year and 3 - year survival of patients, respectively. AUC >0.5 indicates that the prognostic model is meaningful, and the closer to one indicates that the accuracy of the prognostic model is higher. **(H)** This ROC curve compared the ability of clinical characteristics and risk score to predict the prognosis of patients. The area under the red curve represented the prediction ability of risk score.

After the establishment of the prognostic model, we carried out a rigorous validation to ensure that it was capable of accurately predicting the prognosis of all HCC patients. According to our risk scoring formula, patients were divided into high-risk and low-risk groups. We first performed PCA analysis, and the results indicated that regardless of whether the patients were in the TCGA or GEO cohort, we were able to easily distinguish between patients in the high-risk and low-risk groups (Figures 5A,B). Then, we analyzed the survival of patients classified into various risk groups. The results indicated that patients with high risk had significantly worse overall survival (OS) and progression-free survival (PFS) than those with low risk in the TCGA cohort (Figures 5C,D), confirming the predictive ability of our prognostic model. Following that, the risk score was subjected to independent prognostic analysis, and the relationship between the risk score and prognosis of patients were analyzed using single factor analysis and multi-factor analysis, respectively. Without taking other clinical parameters into account, the univariate COX regression analysis revealed that patients with a high-risk score had significantly worse OS than those with a low-risk score (Figure 5E). However, patients’ clinical situations are frequently rather complex, and their prognosis is also affected by a variety of circumstances. As a result, we examine the link between clinical parameters and patient risk scores and repeat the multivariate COX regression analysis. The results continue to indicate that patients’ risk scores are considerably adversely connected with their prognosis (Figure 5F), and both of these correlations are statistically significant. Finally, we determined the reliability of our prognostic model by plotting the receiver operating characteristic curve and calculating the area under the curve (AUC). The area under the receiver operating characteristic curve was 0.799, 0.660, and 0.661 for 1, 2, and 3 years, respectively. As a result, our prognostic model is effective at predicting patients’ prognoses, particularly for the first year (Figure 5G). The receiver operating characteristic (ROC) curve of the prognostic model and other clinical parameters were observed. The prognostic model outperformed other established prognostic factors by a significant margin (Figure 5H).

### The guiding significance of prognosis model for clinical diagnosis and treatment

**FIGURE 6:**
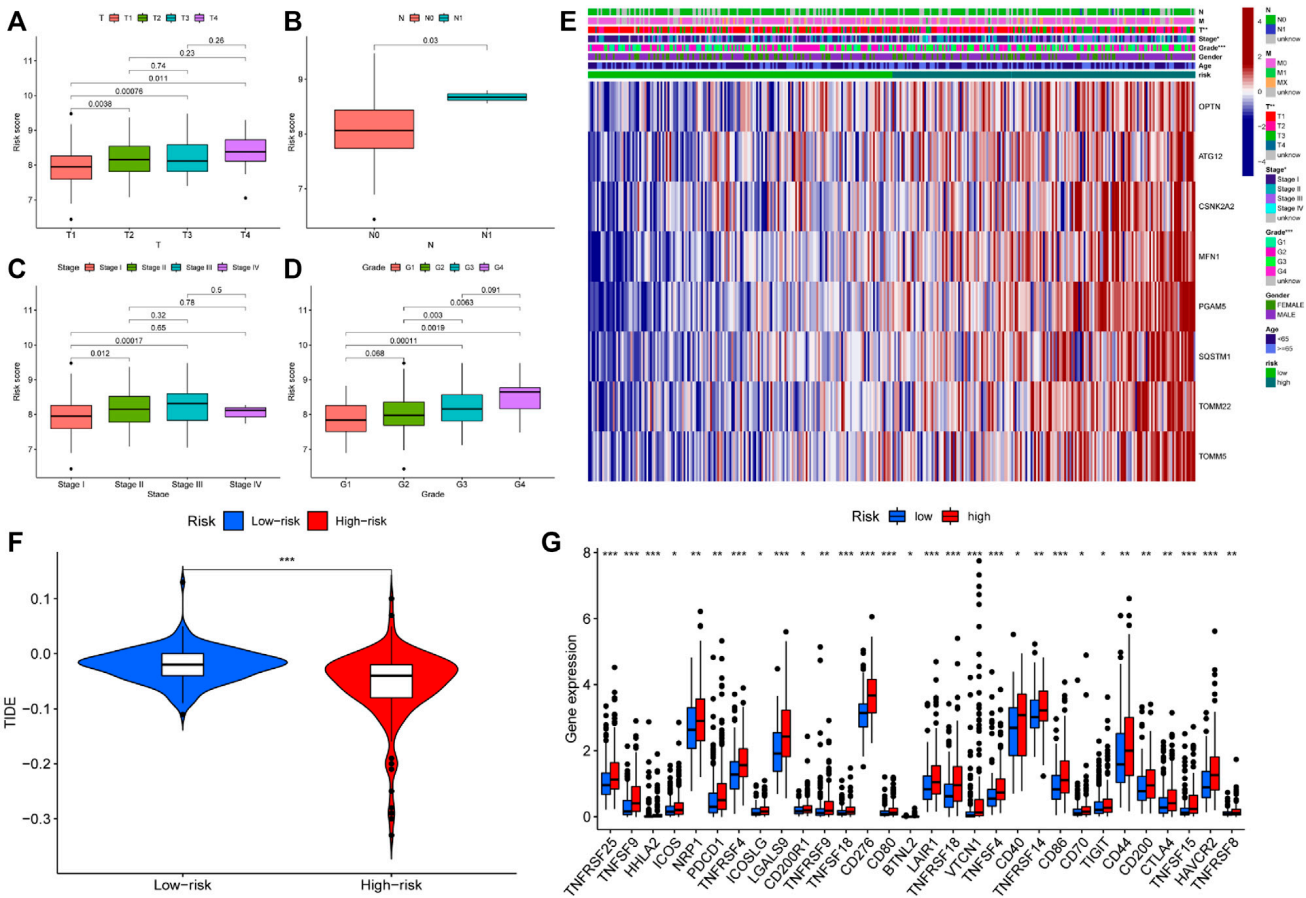
Differences in clinical features and efficacy of immunotherapy among patients with different risk scores **(A–D)** Risk score was correlated with T, N, Stage and Grade; (**E)** The expression of prognostic related genes in clinical factors and different risk groups; (**F)** The violin chart of patients’ response to immunotherapy in high and low-risk groups. Blue represented low-risk group, red represented high-risk group; (**G)** Expression of immune checkpoints in two risk subgroups. * represented *p* < 0.05, ** represented *p* < 0.01, and *** P represented <0.001.

We investigated the role of prognostic risk models in clinical practice and examined if there were differences in clinically relevant variables (such as age, gender, age, grade, stage, T, N, M, and so on) between patients classified as high or low risk. The findings indicated that there was no statistically significant difference in risk scores between patients aged 65 years and younger and those aged 65 years or older. Additionally, no significant variation in risk scores was observed across individuals with different genders and M (metastasis). However, risk scores for distinct T (primary tumor), N (regional lymph node), Stage, and Grade patients were significantly varied. For example, the risk score of T3 patients could be more than that of T1 and T2 patients, N1 patients might have a higher risk score than N0 patients, patients in Stage II could have a higher risk score than patients in Stage I, and G3 patients could have a higher risk score than G1 and G2 patients (Figures 6A–D). Following that, we created a clinical correlation heatmap to visualize it (Figure 6E). Therefore, we proved that the prognosis model has a strong correlation with clinical pathological factors such as T, N, Stage and Grade.

**FIGURE 7:**
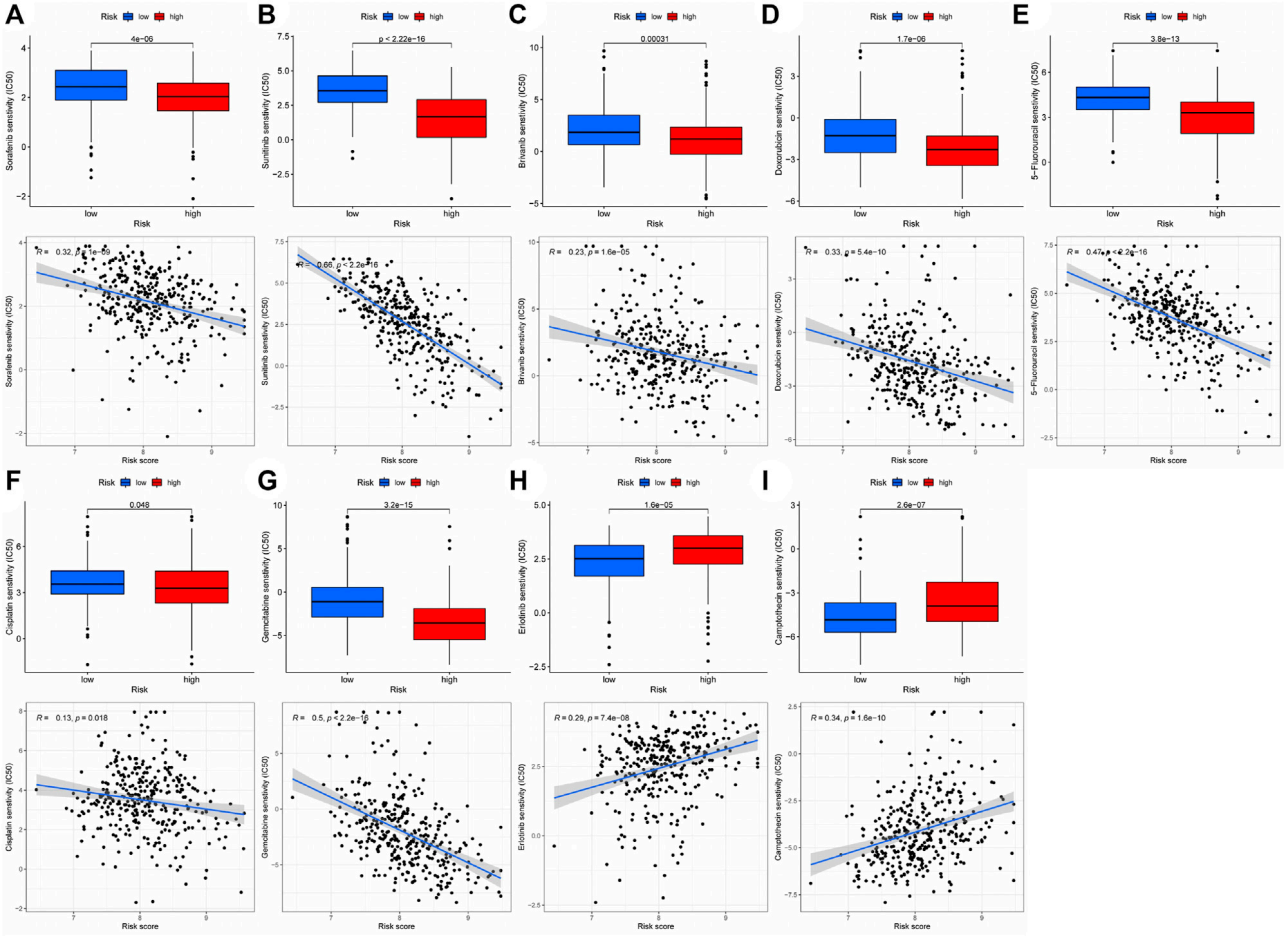
The treatment sensitivity analysis of HCC clinical commonly used drugs in patients with different risk scores **(A–I)** Correlation and sensitivity between different risk scores and drug treatment effect; **(A)** Sorafenib; **(B)** Sunitinib; **(C)** Brivanib; **(D)** Doxorubicin; **(E)** 5-Fluorouracil; **(F)** Cisplatin; **(G)** Gemcitabine; **(H)** Erlotinib; **(I)** Camptothecin.

Immunotherapy is the latest treatment for HCC patients, which is usually combined with traditional chemotherapy methods to achieve better efficacy. However, due to tumor heterogeneity, not all patients are sensitive to immunotherapy. Therefore, we finally evaluated the sensitivity of patients to immunotherapy under different risk scores. Our findings indicated that patients with a low-risk score responded more readily to immunotherapy, and the possibility of tumor immune escape was lower. Such patients would get better remission after immunotherapy in clinical treatment (Figure 6F). Additionally, we examined immune checkpoint expression in different risk groups. Common immune checkpoints in HCC including PD1, CTLA4, CD40, CD44, CD80, and CD86 were significantly increased in high-risk groups of patients (Figure 6G). Drug therapy (both conventional chemotherapy and targeted therapy) is a critical component of HCC treatment. In order to explore the risk score and the guiding significance for HCC drug treatment, we selected common therapeutic drugs for HCC for sensitivity and correlation analysis, and listed the drugs with therapeutic value. The analysis revealed a positive correlation between the sensitivity of Camptothecin and Erlotinib and the patient’s risk score, but a negative correlation between the sensitivity of other drugs and the patient’s risk score (Figures 7A–I). Therefore, Camptothecin and Erlotinib may be more effective in treating patients with high-risk scores, whereas the other drugs may be more effective in treating patients with low-risk scores.

### Relationship between the immune microenvironment and the prognosis model

**FIGURE 8:**
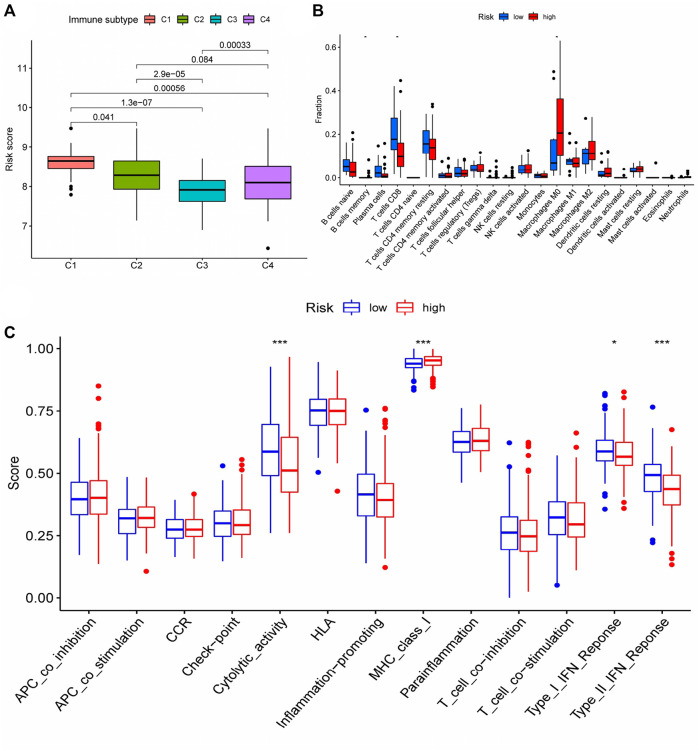
Differences in immune status among patients with different risk scores **(A)** Cluster analysis was performed on the immune cells infiltrated by HCC tissues in patients with different risk groups. **(B)** The number of CD8 + T cells in HCC tissues of patients in the high-risk group was significantly decreased, and the number of M0 macrophages was significantly increased (memory B cells were not discussed due to too few cells); **(C)** Changes of immune function in patients with high-risk group. * represented *p* < 0.05, *** represented *p* < 0.001.

We conducted a cluster analysis on the infiltrated immune cells in HCC tissues and divided them into four subgroups (C1 - C4). Immune typing based on CIBERSORT method, according to the expression of immune-related genes in the expression matrix to identify the corresponding functions of immune cells (such as wound healing, inflammation, etc.), clustering analysis. The results showed that compared with other immune subtypes, C1 patients might have a higher risk score, implying that increased immune cell infiltration in the C1 subgroup may predict poor patient prognosis and that immune cells may play a role in the prognosis of HCC patients (Figure 8A). Therefore, we further analyzed the difference of immune cells infiltrated by HCC tissues in different risk groups. (logFC = 1.5, *p* < 0.05) The infiltration of memory B cell, CD8 + T cells and M0 macrophages was significantly different in patients with different risk groups, according to the findings (Figure 8B). This suggests that changes in the infiltration of these three kinds of immune cells may contribute to the increased risk score of patients, as an important auxiliary cell in the tumor immune microenvironment promotes the malignant progression of tumors. Changes in infiltrated immune cells were bound to lead to changes in immune function, so we finally analyzed the immune function of patients with different risk scores. The results indicated that patients in the high-risk group had decreased cytolytic activity, type I/II IFN response, and type I MHC function, while patients in the low-risk group had increased type I MHC function (Figure 8C), implying that the immune microenvironment of patients in different risk groups had changed, affecting the prognosis of HCC patients to varying degrees.

### Gene mutation among different risk groups

**FIGURE 9:**
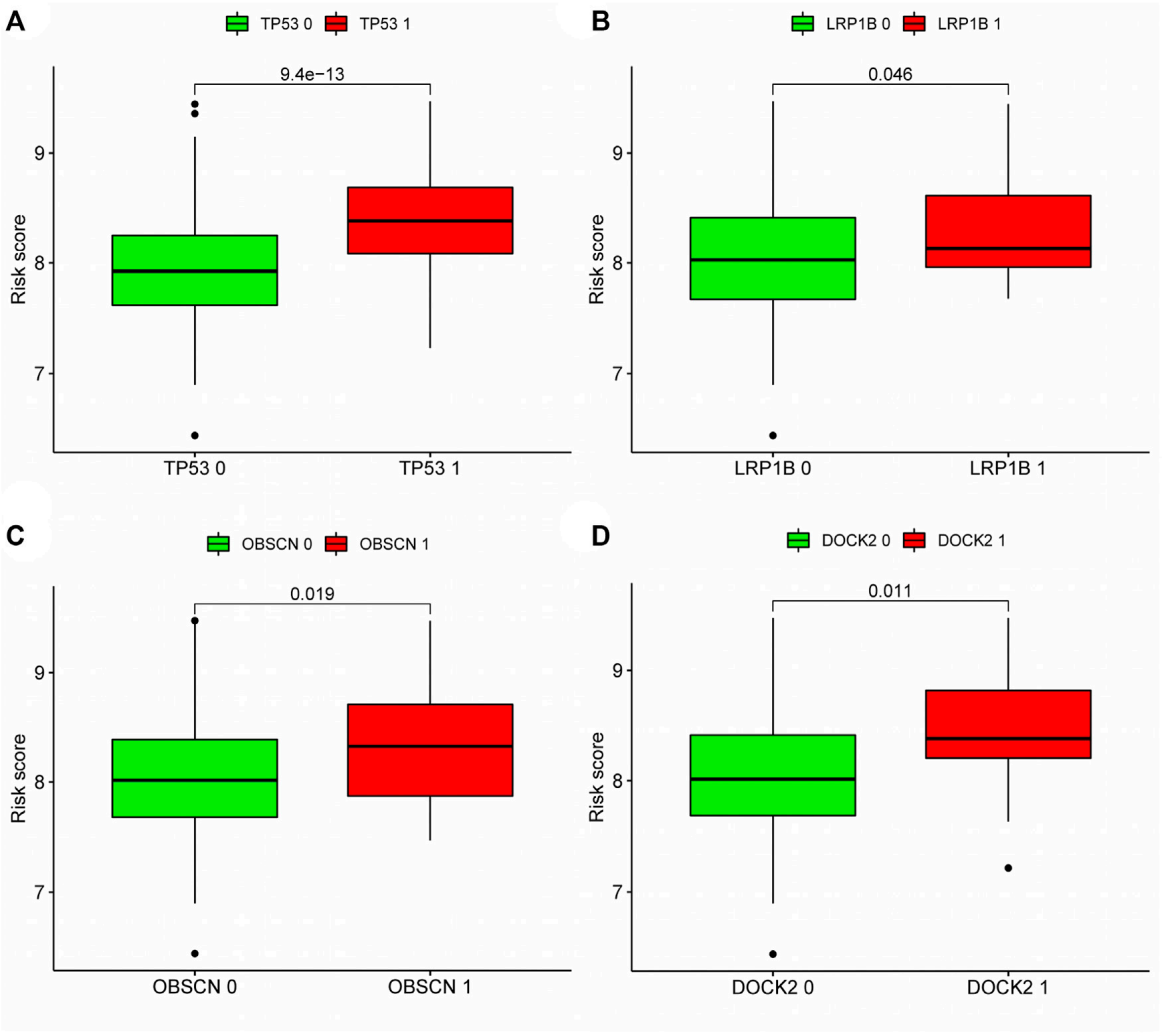
Correlation analysis between top 20 mutation genes and risk score: According to the expression level of mutant genes, patients in the TCGA cohort were divided into high-expression group and low-expression group, and whether there was difference in the risk score between the two groups was compared. **(A–D)** Analyze whether the top 20 genes with the highest mutation frequency are associated with the risk score. Patients with high expression of TP53, LRP1B, OBSCN and DOCK2 had higher risk scores. Green meant low expression of this gene, red meant high expression of this gene. 0: No mutation, 1: mutation.

According to our analysis, a total of 21 out of 362 tissues were detected with mutations, and a total of 12,084 genes in the mutant tissues showed different degrees of mutations. We chose the top twenty genes with the highest mutation frequency to determine whether they expressed differently in high- and low-risk groups. Four of the top twenty most frequently mutated genes (*TP53*, *LRP1B*, *OBSCN*, *DOCK2*) were found to have differential expression between high and low risk groups (Figures 9A–D), suggesting that the mutations in these four genes may contribute to an increased risk score for prognosis.

### Enrichment analysis of DEGs between different risk groups

**FIGURE 10:**
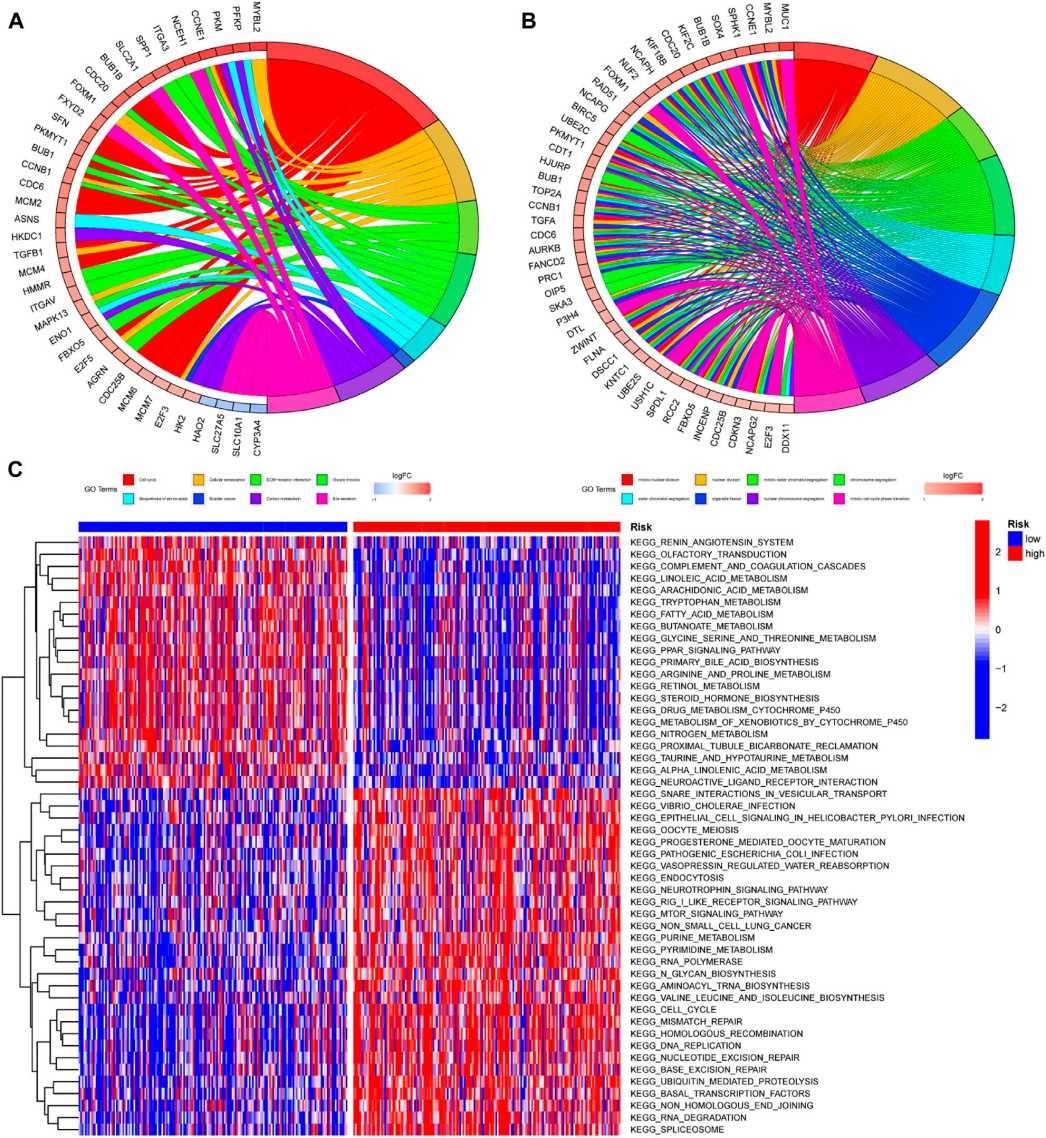
Enrichment analysis of DEGs among patients with different risk scores **(A,B)** GO and KEGG enrichment analysis was performed on genes differentially expressed among patients in different risk groups. The left half of the circle diagram was the gene item, and the right was the annotated function item; (**C)** GSVA analysis could analyze the pathways between patients with different risks, and the results were shown in the heatmap. Blue meant the pathway down, red meant the pathway up. The blue horizontal axis above the figure represented the low-risk group, and the red horizontal axis represented the high-risk group.

In order to further investigate the differences among patients with different risk groups under our prognostic model typing, we analyzed the gene expression among patients with high and low risk scores and obtained 756 DEGs. The DEGs were analyzed for GO and KEGG enrichment. According to the GO enrichment analysis, the primary enrichment function of DEGs was mitotic nuclear division, nuclear division, and so on (Figure 10A). KEGG enrichment analysis revealed that DEGs were primarily enriched for functions related to the cell cycle, cellular senescence, and ECM receptor interaction (Figure 10B). In addition, we used Gene Set Variation Analysis (GSVA) to determine which pathways or functions were up-regulated or down-regulated in high and low-risk groups. Heatmap was used to visualize the analysis results. The heatmap showed that compared with the patients in the low-risk group, the renin-angiotensin system, complement and coagulation cascade reactions in the high-risk group were significantly inhibited, while the vesicle transport function and Rig-I-like receptor were significantly activated. (Figure 10C).

### PPI analysis of DGEs

**FIGURE 11:**
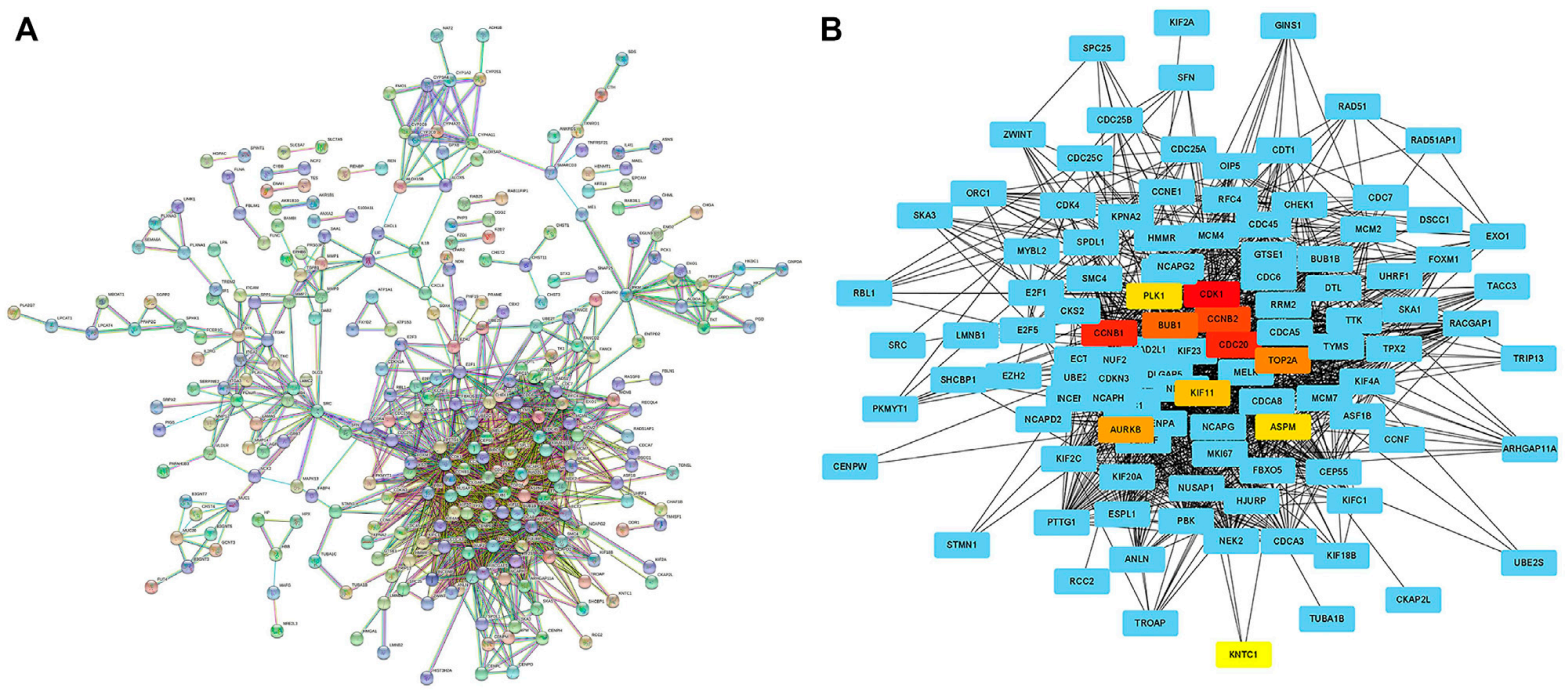
Searching network core genes by PPI analysis **(A)** PPI analysis of differentially expressed genes between different risk groups was performed on STRING online website (high confidence = 0.9); **(B)** The PPI analysis results were imported into Cytoscape software to search for network core genes, which were marked in non-blue.

**FIGURE 12:**
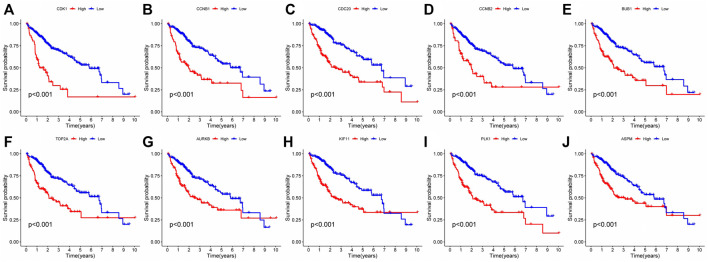
Single gene survival analysis of network core genes: **(A–J)** Single-gene survival analysis was performed on the top 10 network core genes. Red lines represented high-risk group, blue lines represented low-risk group.

**FIGURE 13:**
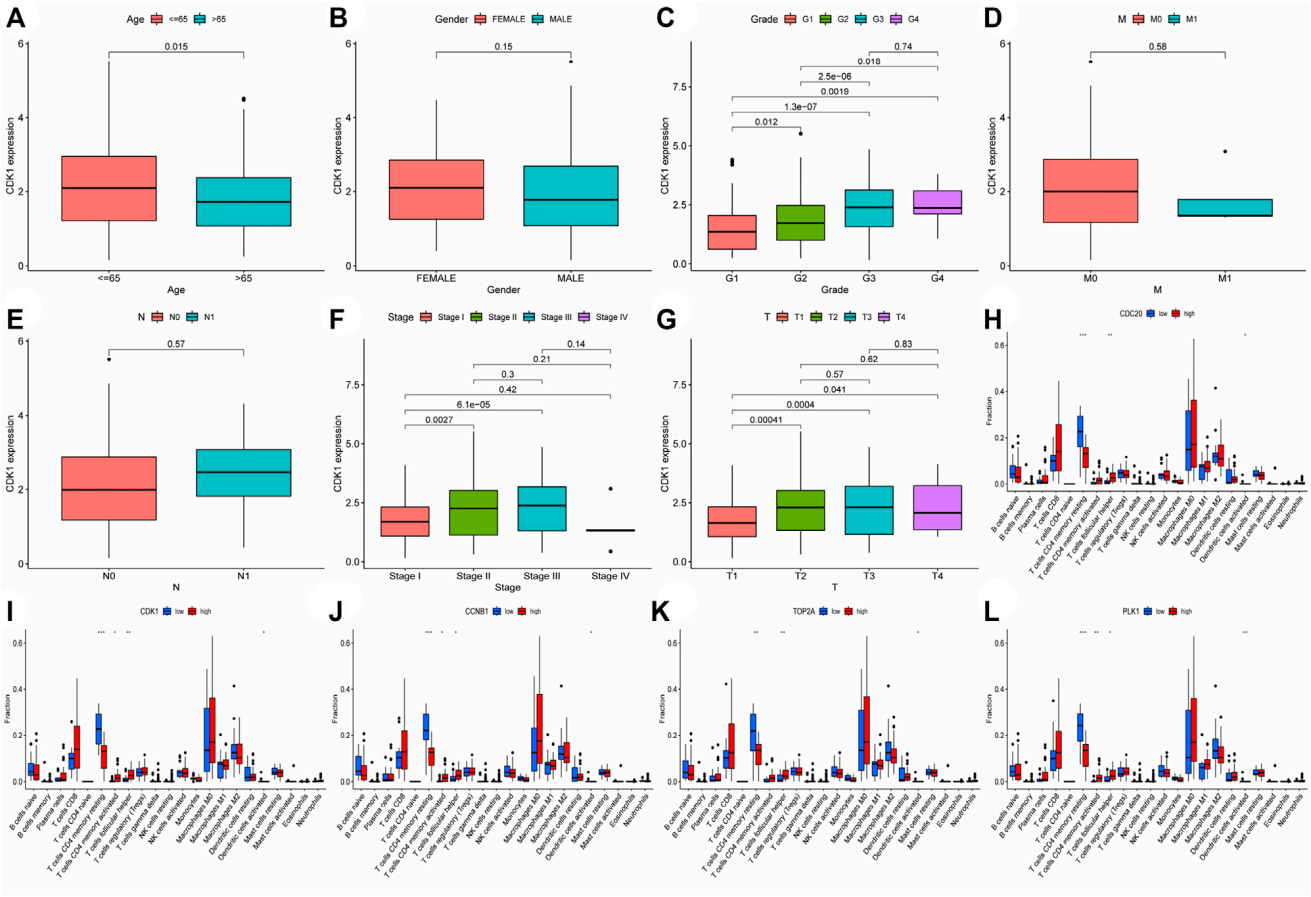
Analysis of clinical and immune correlation of core network genes **(A–G)** Clinical correlation analysis was performed on 10 core network genes, including gender, age, TNM stage, Stage and Grade (CDK1 analysis was shown only, other result could be seen in supplementary documents (Data Sheet 1); (**H–L)** Immunocyte infiltration of 10 core genes in the network was analyzed (only the results of the first five genes were shown, other result could be seen in supplementary documents (Data Sheet 2). * represented *p* < 0.05, ** represented *p* < 0.01, *** represented *p* < 0.001.

In order to analyze the interaction between genes and find valuable core genes, we screened differentially expressed genes for protein interaction analysis on STRING online website, and selected strongly associated genes according to the standard of high confidence = 0.9 (Figure 11A). Subsequently, the core genes were analyzed and visualized in Cytoscape software, and 10 network core genes were finally selected (Figure 11B). We performed single-gene survival analysis of these 10 core genes. Survival analysis revealed a negative correlation between these ten genes and patient prognosis (Figures 12A–J). Then, we repeated the clinical and immune correlation analyses on these ten genes. The results indicated that the expression levels of the majority of genes were not related to the patients’ age, gender, or M, but increased in correlation with the T, N, Stage, and Grade levels (Figures 13A–G). Additionally, the expression of core genes was positively correlated with the infiltration of CD4 + T memory cells and T follicular helper cells in the majority of high-risk groups (Figures 12H–L).

## Discussion

Researchers discovered several decades ago that cancer cells were more likely to continue energy metabolism via glycolysis even under aerobic settings, a phenomenon dubbed the Warburg effect. This impact is regarded as strong evidence for a link between cancer and mitochondrial malfunction (Koppenol et al., 2011; Zong et al., 2016). Subsequent studies discovered widespread alterations in mitochondrial genes in tumor tissues, implying that mitochondrial production and metabolic activities were changed. Retrograde signals between mitochondria and tumor cells govern this process (Wallace, 2012). Due to the presence of reactive oxygen species and other variables in the mitochondrial environment, mitochondria are readily damaged or even altered. These damaged mitochondria are repaired or cleared by Mitophagy. Mitophagy is a highly specific quality control process that has been linked to a variety of physiological processes, including early embryonic development, cell differentiation, inflammation, and apoptosis (Onishi et al., 2021). If the function of mitophagy was aberrant, damaged and mutant mitochondria survive, encouraging the emergence and progression of malignancies (Chang et al., 2017). Our research focused on the link between HCC and mitophagy, as well as the expression of MRGs in HCC patients and predictive genes. We created a prognostic model with the aid of LASSO regression. The model’s reliability was then confirmed, and its potential was investigated. Finally, we demonstrated that the model had clinical use.

In addition to participating in the regulation of mitochondrial autophagy function, previous studies have confirmed that mitochondrial autophagy-related gene sets are related to other pathophysiology of HCC. CDC37 can promote HCC progression by regulating cell cycle (Wang et al., 2015). CSNK2B promoted HCC cell proliferation, migration and angiogenesis (Xiao et al., 2020). MAP1LC3A can prevent iron death of hepatic stellate cells in HCC (Zhang et al., 2020a). MFN1 can change the glucose metabolism level of HCC (Zhang et al., 2020b). PGAM5 expression induced chemotherapy resistance by enhancing Bcl-xL signal (Cheng et al., 2018). RPS27A, SRC are associated with HBV-related HCC (Fatima et al., 2012; Hu et al., 2021). Other genes were not found to be associated with other specific HCC subtypes. In additionally, MFN2 was found to have heterozygous loss in HCC patients (Qu et al., 2013). Chang Hyeok An et al. found that somatic mutation and loss of ATG5 gene expression may play a role in the pathogenesis of gastrointestinal cancer by changing autophagy and apoptosis cell death (An et al., 2011). SRC-mediated co-activation of anti-tumor target genes inhibits MYC-induced liver cancer (Suresh et al., 2017).

This prognostic model consists of eight MRGs, namely *OPTN*, *ATG12*, *CSNK2A2*, *MFN1*, *PGAM5*, *SQSTM1*, *TOMM22*, and *TOMM5*. Existing studies have shown that *ATG12* promotes HCC by participating in a variety of long non-coding RNAs (Li et al., 2019; Wei et al., 2019), and *MFN1* reduces oxygen consumption and ATP production in HCC cells by promoting mitochondrial fusion (Li et al., 2020). *PGAM5* can inhibit BAX and cytochrome C-mediated apoptosis signal transduction by stabilizing Bcl-xl to obtain chemotherapy tolerance for HCC (Cheng et al., 2018). *SQSTM1* was found to bind to p62 to form a cohesive protein, binding to and isolating Kelch-like ECH-associated protein 1 (Keap1) to prevent NRF2 degradation (Feng et al., 2021). At the moment, no study exists on the mechanism through which *OPTN*, *CSNK2A2*, *TOMM22*, and *TOMM5* promote HCC. Existing research supports our conclusion that the eight MRGs in the prognostic model contribute to the formation and progression of HCC and can be used in combination as a risk factor for poor prognosis in HCC.

Our findings have significant clinical and pathological implications. There was no statistically significant difference in risk scores between patients of different ages, genders, and M, but risk scores frequently increased as the level of T, N, Grade, and Stage increased, suggesting that these eight genes affected some behaviors of tumor tissues such as tumor proliferation, lymph node metastasis, and had nothing to do with vascular metastasis. Systemic pharmacological therapy, including conventional chemotherapy and immunotherapy, is critical in the clinical treatment of HCC. We assessed the drug sensitivity of 251 medicines included in the “pRRophetic” package and identified and demonstrated the therapeutically available medications for the treatment of HCC (Carcinoma, 2021). The routinely used cytotoxic chemotherapeutic agents for HCC, such as Doxorubicin (Abou-Alfa et al., 2016), 5-Fluorouracil (Lyu et al., 2022), Cisplatin (Aramaki et al., 1990), Gemcitabine (Zhu et al., 2006) were considerably more sensitive to treatment in individuals with low risk scores compared to those with high risk scores. On the other hand, other medications, such as Camptothecin (Rowinsky et al., 2000) demonstrated efficacy in high-risk individuals. Since the FDA approved the first targeted medication, sorafenib, in 2007 for advanced HCC, targeted therapy has become an integral aspect of HCC treatment. The drug sensitivity analysis of the first-line medication Sorafenib (Cheng et al., 2009) for advanced HCC revealed that individuals with a low-risk score had considerably higher treatment sensitivity than those with a high-risk score. Additionally, we added additional small molecule inhibitors for HCC treatment, including Sunitinib (Cheng et al., 2013), Brivanib (Johnson et al., 2013), and Erlotinib (Zhu et al., 2015) Sunitinib and Brivanib had greater sensitivity in low-risk patients, while Erlotinib shown more benefit in high-risk patients. Finally, we assessed the probability of treatment failure due to immune escape in immunotherapy-treated HCC patients. Treatment failure occurred at a significantly higher rate in the high-risk group than in the low-risk group. These have important reference value for clinical drug therapy of HCC.

Immune checkpoints are molecules that protect the human immune system from inflammatory damage caused by T cell activation that is too strong. Tumor cells take advantage of this feature of the human immune system to suppress immune responses by overexpressing immune checkpoint molecules, allowing the immune system to escape (Diesendruck and Benhar, 2017; Lim et al., 2017). As discussed previously, the most frequently used targeted therapies for HCC, such as sorafenib, are selective inhibitors of the immunological checkpoint protein programmed death receptor-1 (PD-1, encoded by *PDCD1* (Palamarchuk et al., 2022). Therefore, we examined immune checkpoint expression in individuals belonging to various risk groupings. Almost all immune checkpoint expression levels were significantly higher in the high-risk group, indicating not only that the tumors in patients with high-risk scores were more malignant, but also that patients with high-risk scores were better candidates for targeted immune checkpoint drugs like sorafenib.

The tumor immune microenvironment (TME) is a dynamic system composed of cancer cells, a complex cytokine environment, extracellular matrix, and immune cell subsets (Fu et al., 2019), which has been shown in recent years to play a critical role in the progression of tumors. Immune cells, as a critical component of TME, not only fail to play an anti-tumor role when tumor cells and cytokines secreted by them are present, but also inhibit the body’s immune response against tumor cells (Lu et al., 2019; Oura et al., 2021). As a result, we examined the extent to which immune cells infiltrated tumor tissue in our investigation. We observed a decrease in CD8 + T cells and an increase in M0 macrophages in patients with a high-risk score. CD8 + T cells directly contributed to tumor eradication through the secretion of perforin, granzyme, and TNF or through their binding to Fas-FasL in tumor tissues, and their expansion may help patients live longer (Moreno-Cubero and Larrubia, 2016; Oura et al., 2021). M0 macrophages are a type of non-polarized macrophage that lacks anticancer activity. In addition, we also found that the function of Cytolytic activity, Type I IFN Reponse, Type II IFN Reponse decreased and the function of MHC class I increased slightly in patients with high-risk scores. This all indicated that patients with a high-risk score had a weakened ability to fight cancer.

After further examination of the prognostic model, we discovered that the differential genes of distinct risk groups contain a large number of significantly related core genes. Clinical connection, survival analysis, and immunological correlation all indicate that these core genes are extremely valuable HCC-related genes. Thus, in addition to the eight MRGs, these network core genes need additional investigation into their mode of action in HCC.

We noted that four previous articles were similar to our research, and we analyzed the similarities and differences between them. These four articles were roughly the same as our research in the overall research method, and all the subjects were hepatocellular carcinoma. But the largest difference was the selection of genes. The mitophagy-related genes selected by Tao Zhang et al. referred to genes that played an important role in mitochondrial metabolism, but we mainly focused on genes that played a key role in mitochondrial autophagy. Shengwei Shen et al. selected autophagy-related genes as the research object. However, the range of autophagy was large, and mitophagy was only a special type. Junbin Yan et al. selected apoptosis-related genes, apoptosis was a kind of programmed cell death, which was related to the changes of cell morphology and structure, and was related to mitophagy but not coincide. Finally, the research object of Hao Chen et al. was completely consistent with us, but the research methods were completely different. Firstly, the patients were divided into two groups according to the median immune score in their studies, and the MRGs between the two groups were searched for to construct the prognosis model. We chose MRGs that were differentially expressed between normal tissues and tumor tissues which were related to the prognosis of HCC patients, and used them to construct the prognosis model according to the different expression levels. Therefore, although the four studies were similar to our research, the final prognosis models were not the same. In summary, our research was not repeat the previous steps, but also had great innovation.

This study has limitations of its own. To begin, there is no experimental validation, which diminishes the credibility of our findings. In addition, the “pRRophetic” package was developed and maintained by Paul Geeleher et al. (Lee et al., 2010; Joo et al., 2011; Geeleher et al., 2014; Nezich et al., 2015; Chen et al., 2016; Lesage et al., 2016; Di Rita et al., 2018; Kravic et al., 2018; Setz et al., 2018; Sekine et al., 2019; Yao et al., 2019; Inokuchi et al., 2021; Towers et al., 2021; Yao et al., 2021); (Twig et al., 2008; Geisler et al., 2010; Bertolin et al., 2013; Fu et al., 2020; Yan et al., 2020; Yang et al., 2020; Iorio et al., 2021; Ma et al., 2022; Shang et al., 2022; Zheng et al., 2022) in 2014 as a tool for predicting clinical chemotherapy responses from tumor gene expression levels. However, as we know, the pRRophetic package has not been updated since 2017, so a considerable number of new drugs with good clinical efficacy and potential, such as PD-1/PD-L1 receptor blockers, have not been included in drug sensitivity analysis, which makes it impossible to predict the clinical efficacy and the possibility of drug resistance of these new drugs to guide clinical treatment.

## Conclusion

In conclusion, we investigated the association between mitochondrial autophagy-related genes and HCC prognosis and developed an eight-gene prognostic model. This prognostic model was highly predictive of the prognosis of HCC patients and could be used to guide clinical diagnosis and treatment. In addition, our study supports a more in-depth study of the mechanisms of these eight mitochondrial-related genes and 10 network core genes in HCC.

## Data Availability

The original contributions presented in the study are included in the article/supplementary material, further inquiries can be directed to the corresponding authors.

## Abbreviations

HCC, Hepatocellular carcinoma; ICC, Intrahepatic cholangiocarcinoma; MRGs, Mitophagy-related genes; DEGs, Different expression genes; PCA, principal component analysis; KM, Kaplan-Meier; OS, Overall survival; PFS, Progression free survival; HR, Hazard ratio; LASSO, Least absolute shrinkage and selection operator; ROC, receiver operating characteristic curve; AUC, Area under curve; GSVA, Gene set variation analysis; PPI, Protein-protein interaction.
