# Supplementary figures and images for: The prognostic value and clinical significance of mitophagy-related genes in hepatocellular carcinoma
Source: Front Genet. 2022 Aug 5;13:917584. doi: 10.3389/fgene.2022.917584 (PMC9388833; doi:10.3389/fgene.2022.917584)

Age 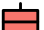 <=65 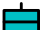 >65

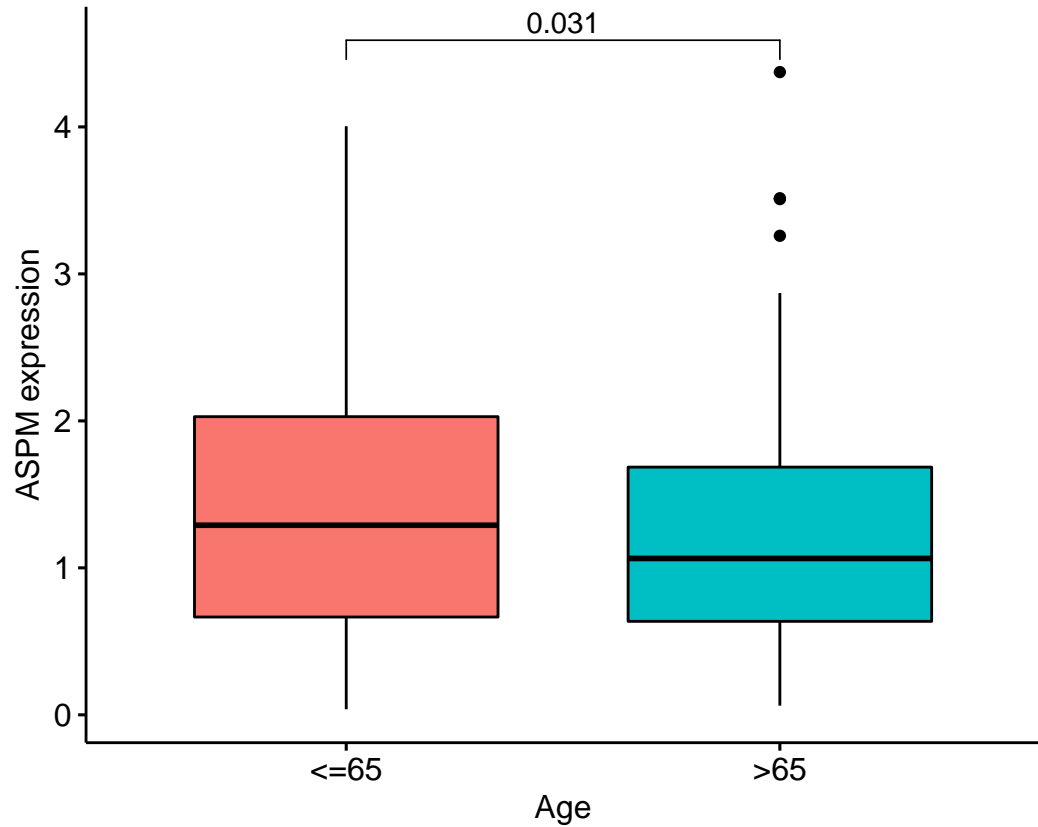

Supplement: Supplementary file 1 [file DataSheet1.zip › ASPM/Age.pdf]

Gender 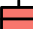 FEMALE 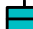 MALE

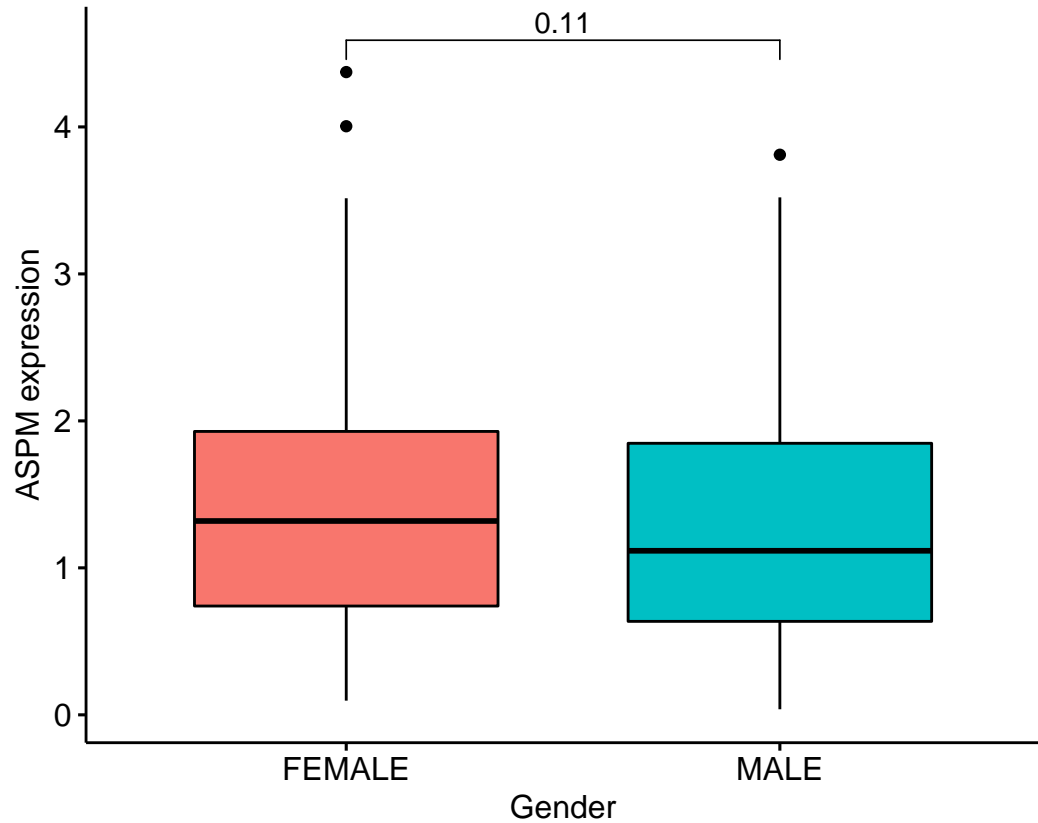

Supplement: Supplementary file 1 [file DataSheet1.zip › ASPM/Gender.pdf]

Grade G1 G2 G3 G4

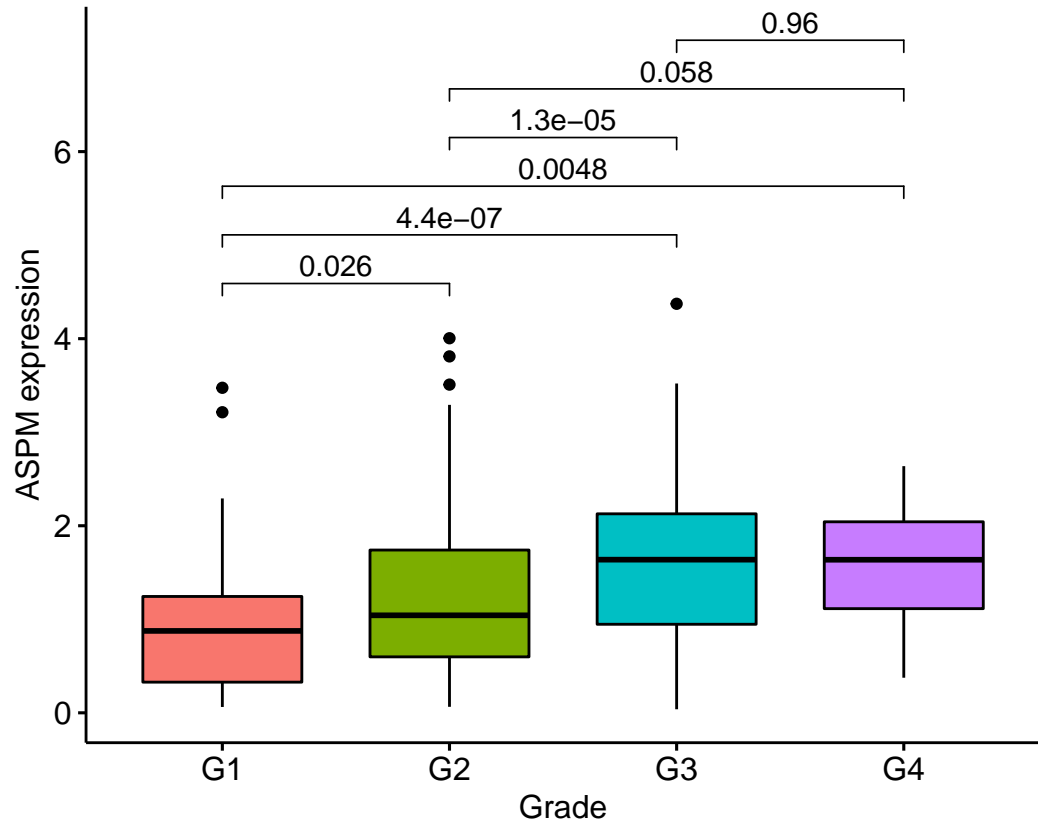

Supplement: Supplementary file 1 [file DataSheet1.zip › ASPM/Grade.pdf]

M M0 M1

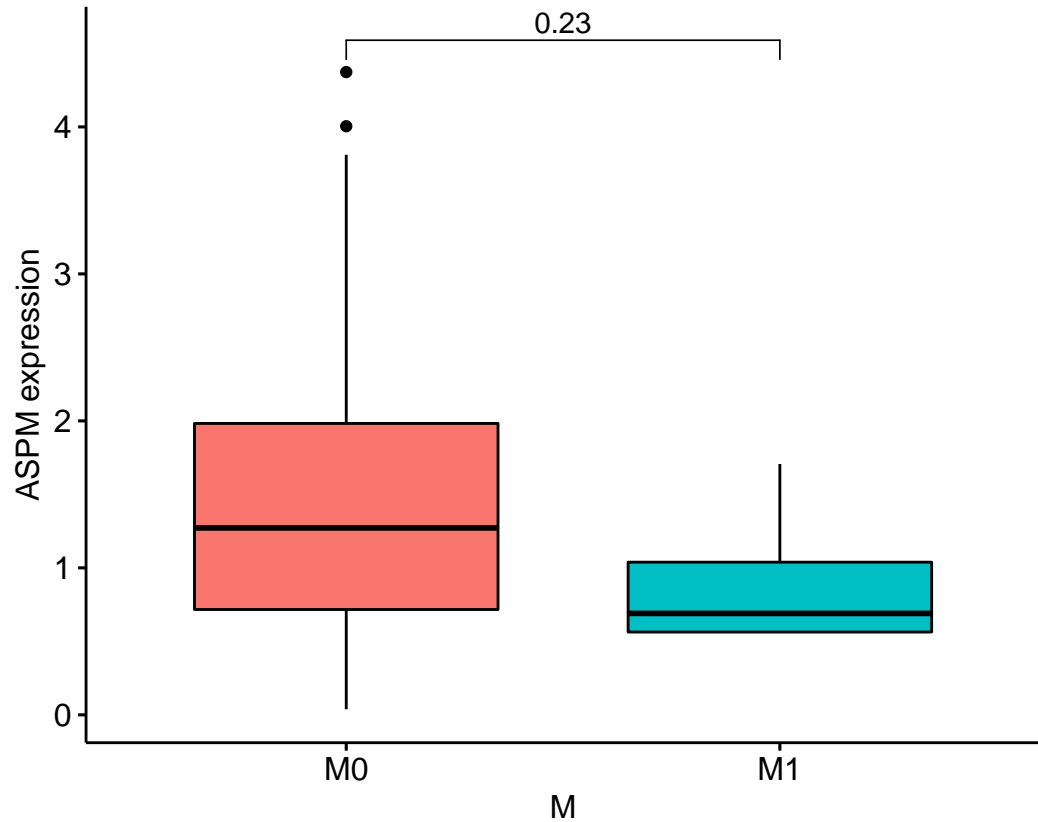

Supplement: Supplementary file 1 [file DataSheet1.zip › ASPM/M.pdf]

N N0 N1

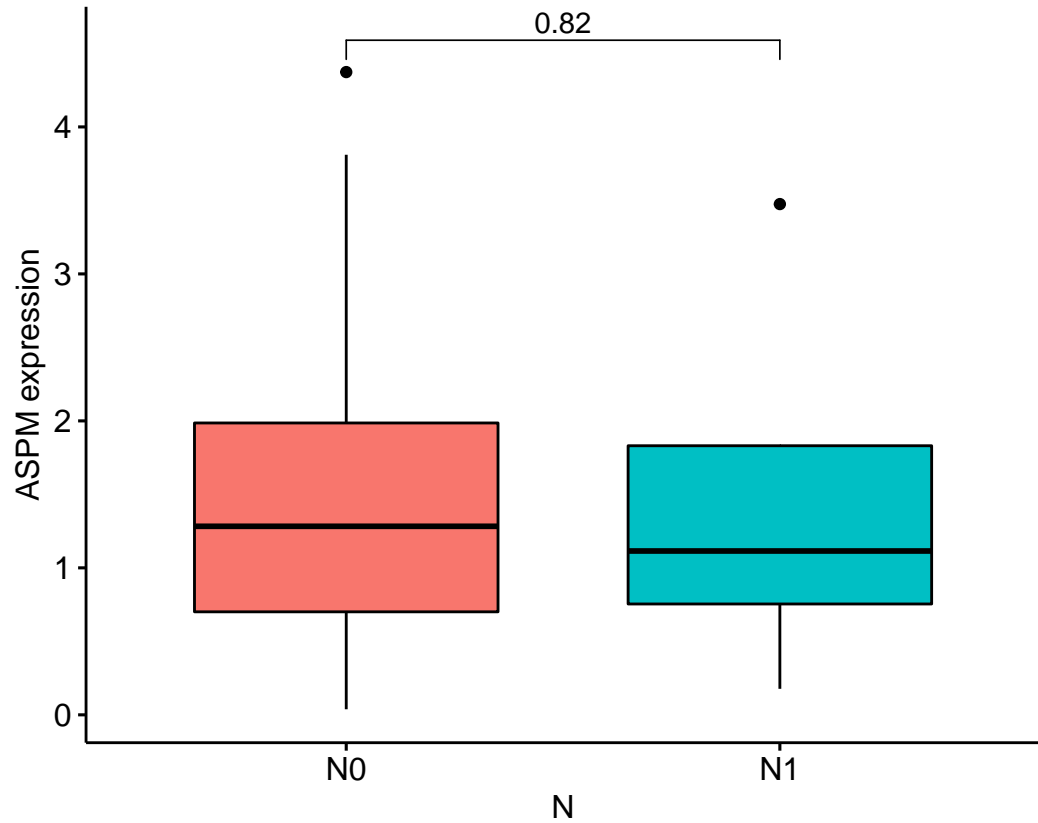

Supplement: Supplementary file 1 [file DataSheet1.zip › ASPM/N.pdf]

Stage I Stage II Stage III Stage IV

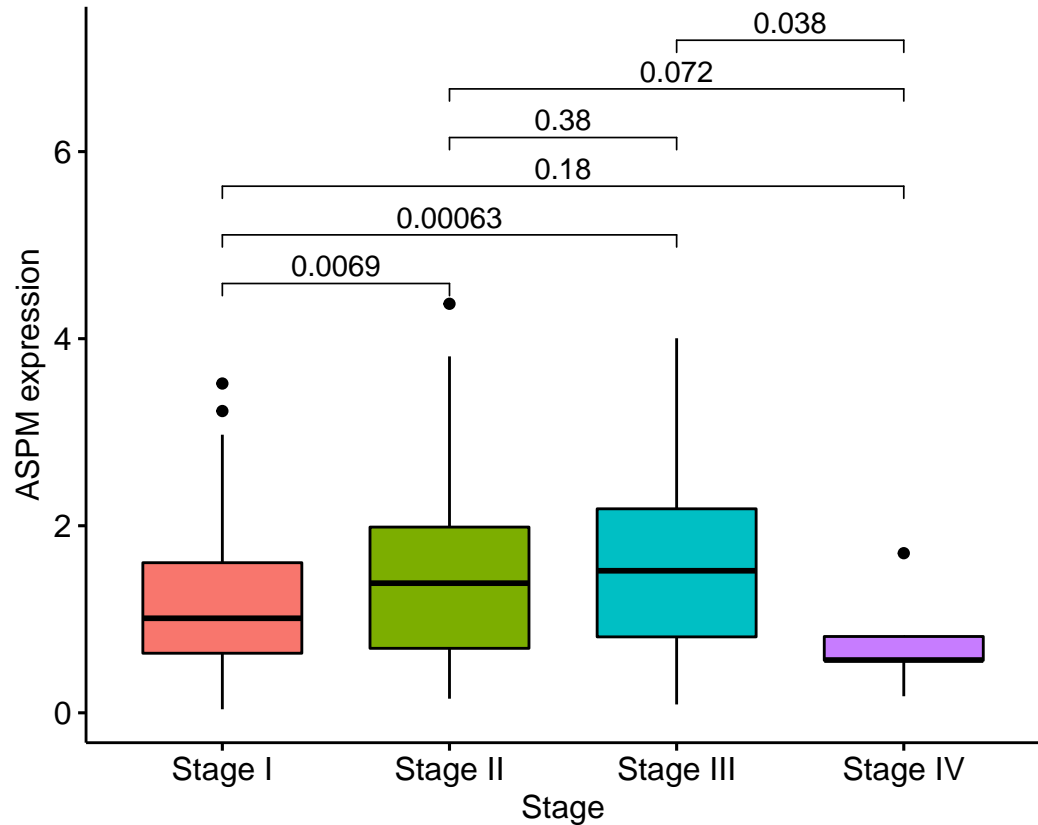

Supplement: Supplementary file 1 [file DataSheet1.zip › ASPM/Stage.pdf]

T T1 T2 T3 T4

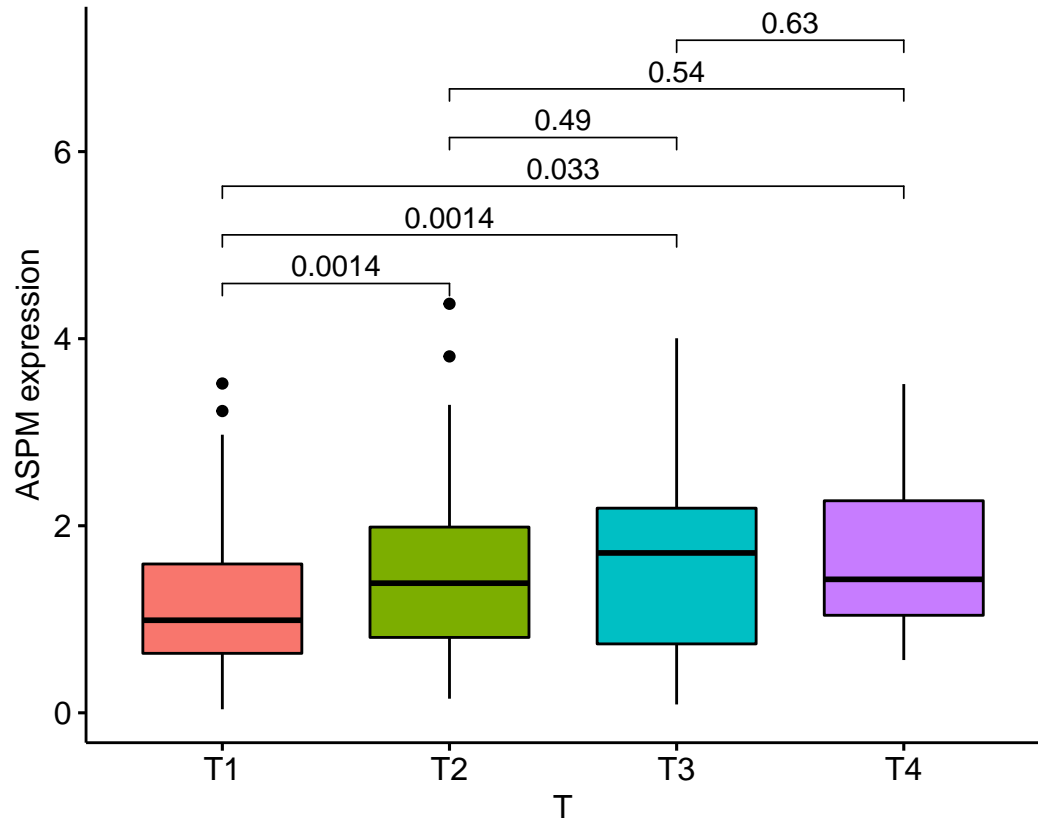

Supplement: Supplementary file 1 [file DataSheet1.zip › ASPM/T.pdf]

Age 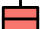 <=65 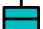 >65

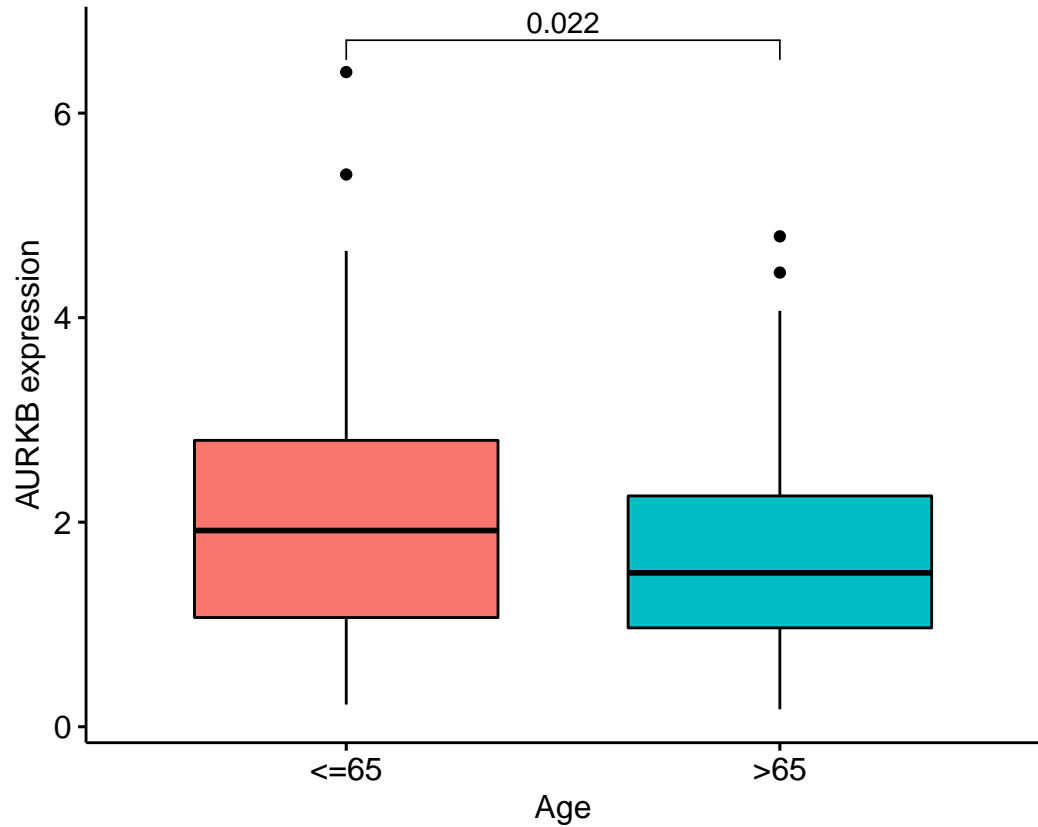

Supplement: Supplementary file 1 [file DataSheet1.zip › AURKB/Age.pdf]

Gender 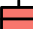 FEMALE 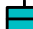 MALE

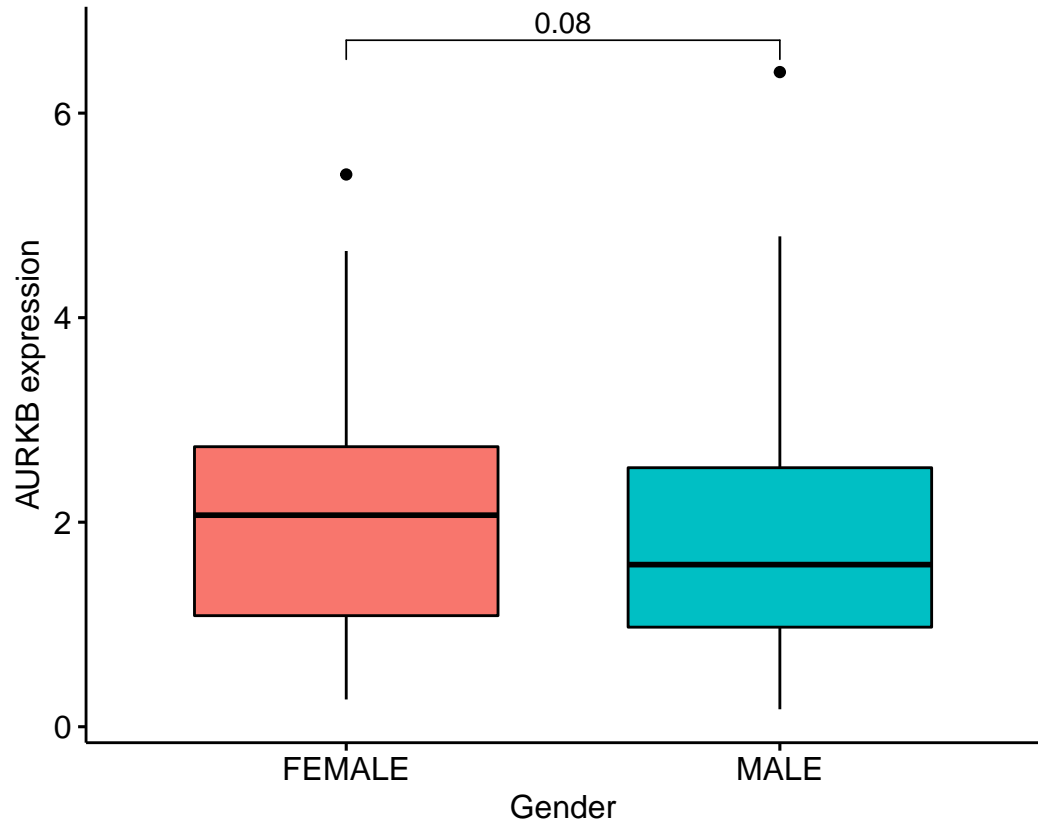

Supplement: Supplementary file 1 [file DataSheet1.zip › AURKB/Gender.pdf]

Grade G1 G2 G3 G4

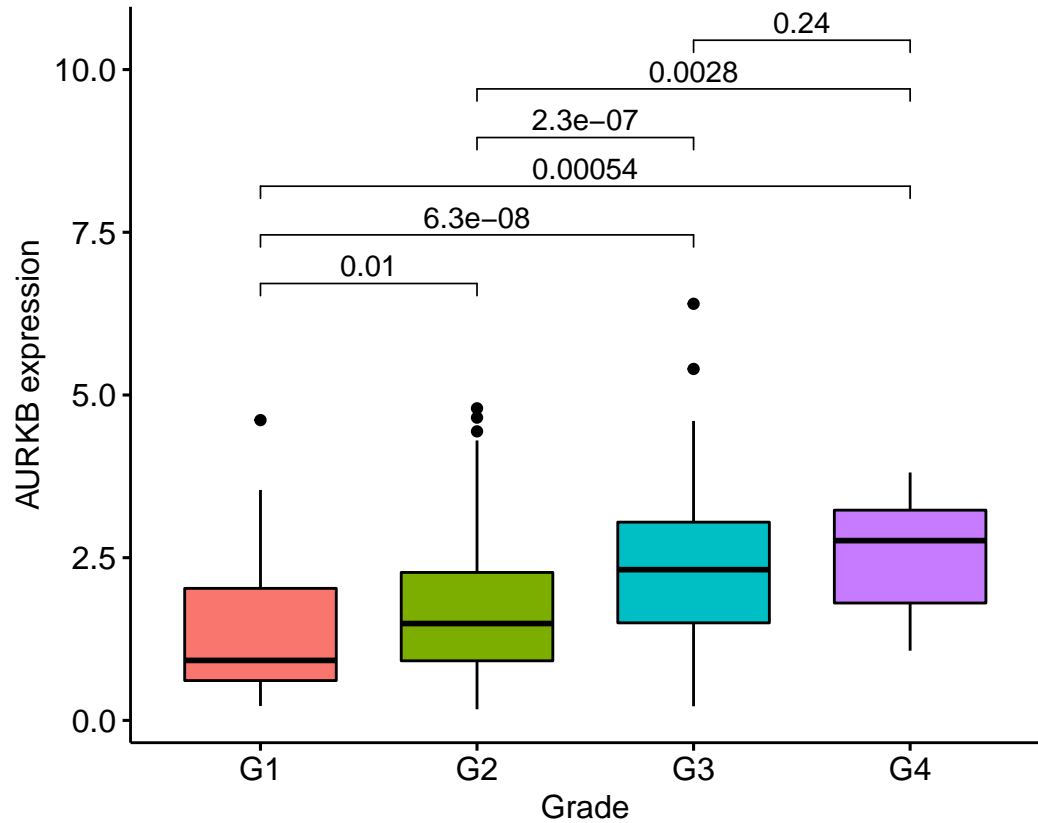

Supplement: Supplementary file 1 [file DataSheet1.zip › AURKB/Grade.pdf]

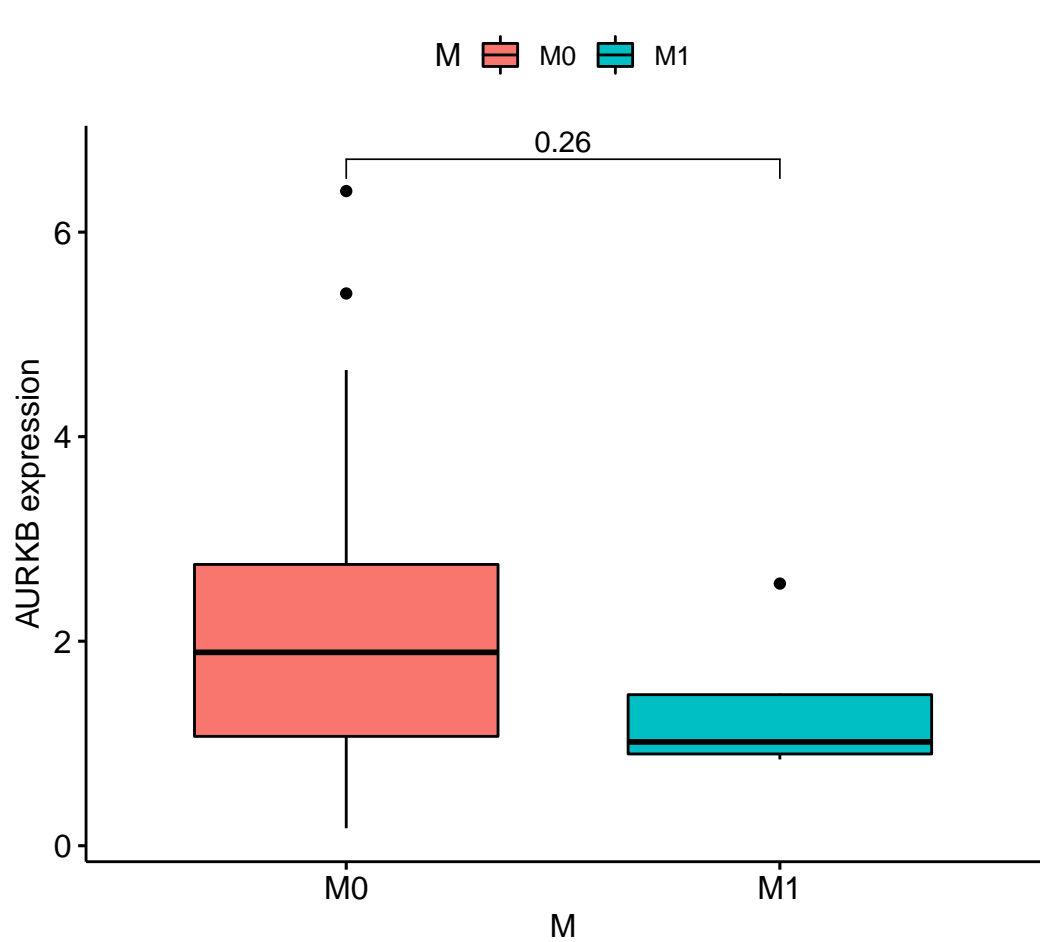

Supplement: Supplementary file 1 [file DataSheet1.zip › AURKB/M.pdf]

AURKB expression

N N0 N1

0.92

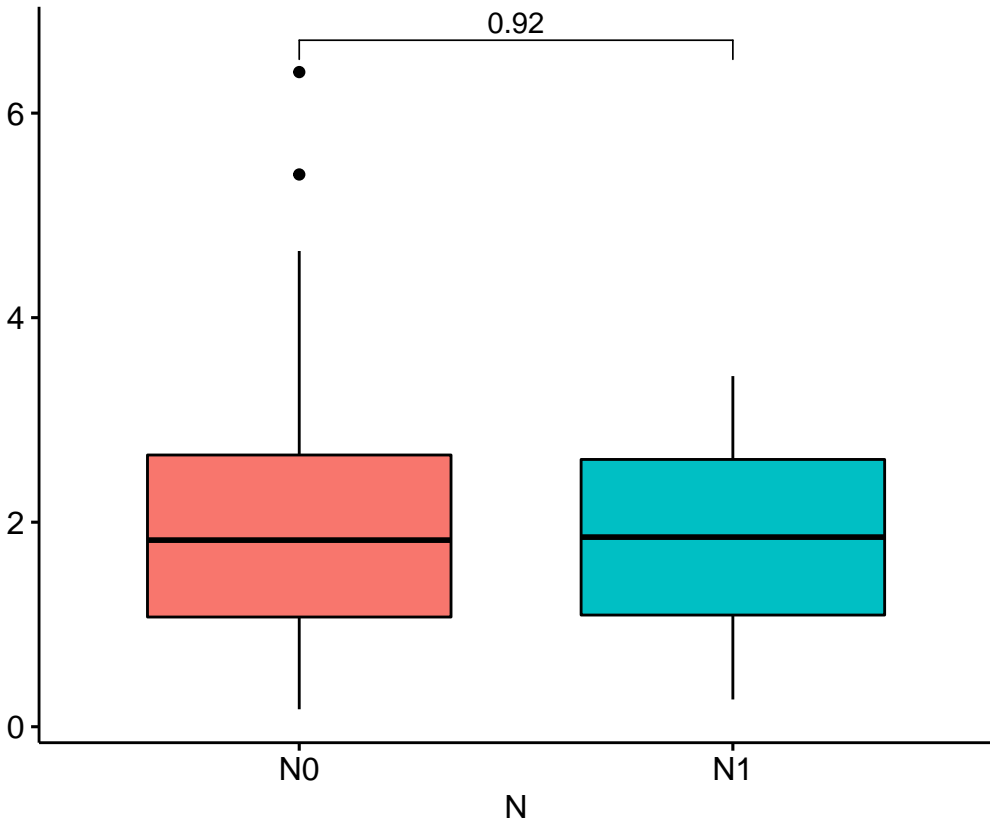

Supplement: Supplementary file 1 [file DataSheet1.zip › AURKB/N.pdf]

Stage 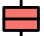 Stage I 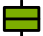 Stage II 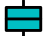 Stage III 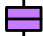 Stage IV

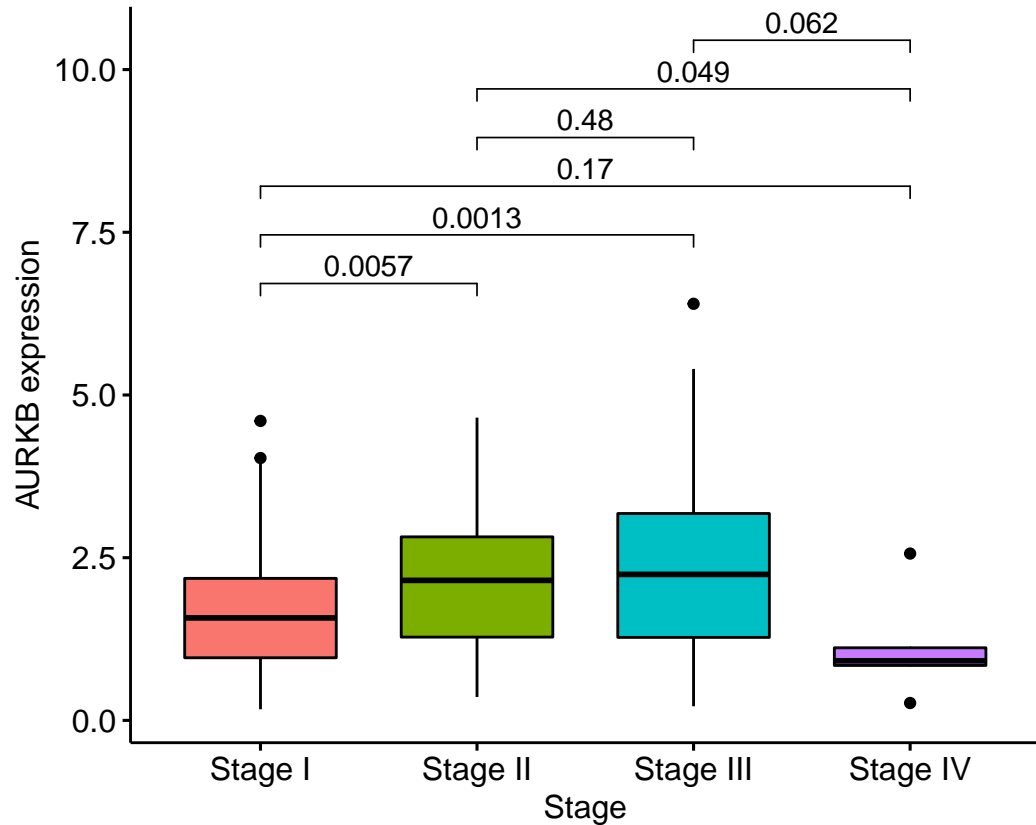

Supplement: Supplementary file 1 [file DataSheet1.zip › AURKB/Stage.pdf]

AURKB expression

T T1 T2 T3 T4

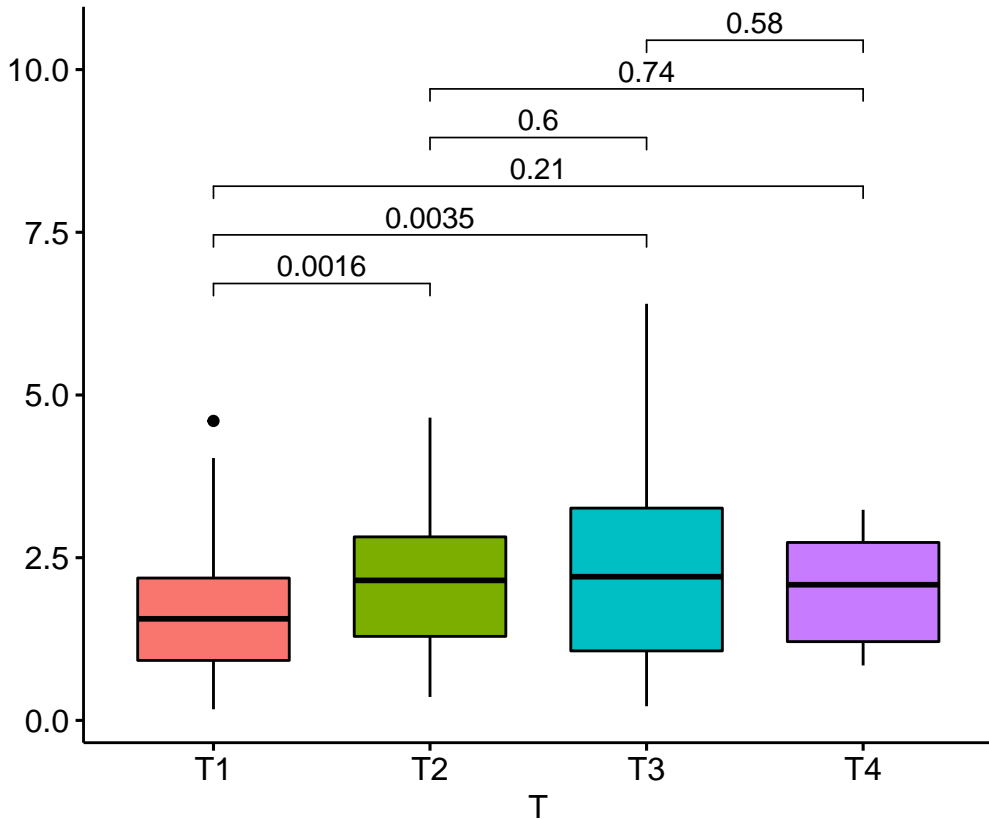

Supplement: Supplementary file 1 [file DataSheet1.zip › AURKB/T.pdf]

Age 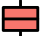 <=65 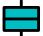 >65

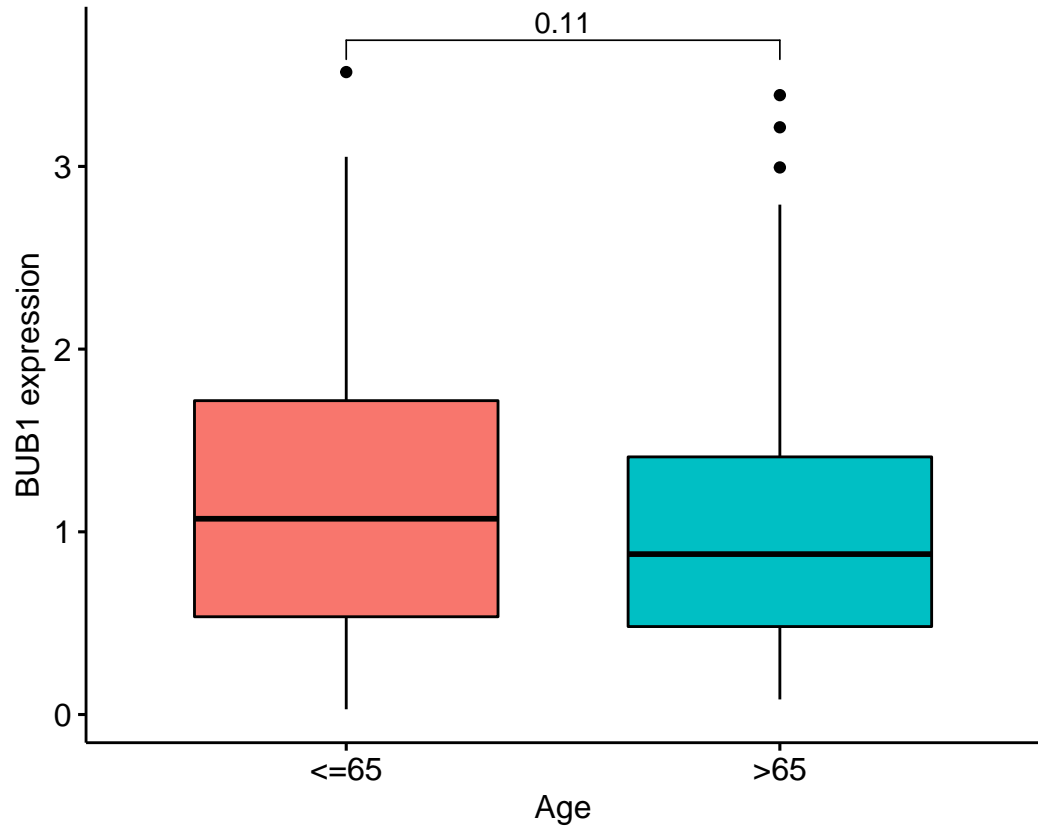

Supplement: Supplementary file 1 [file DataSheet1.zip › BUB1/Age.pdf]

Gender 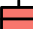 FEMALE 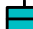 MALE

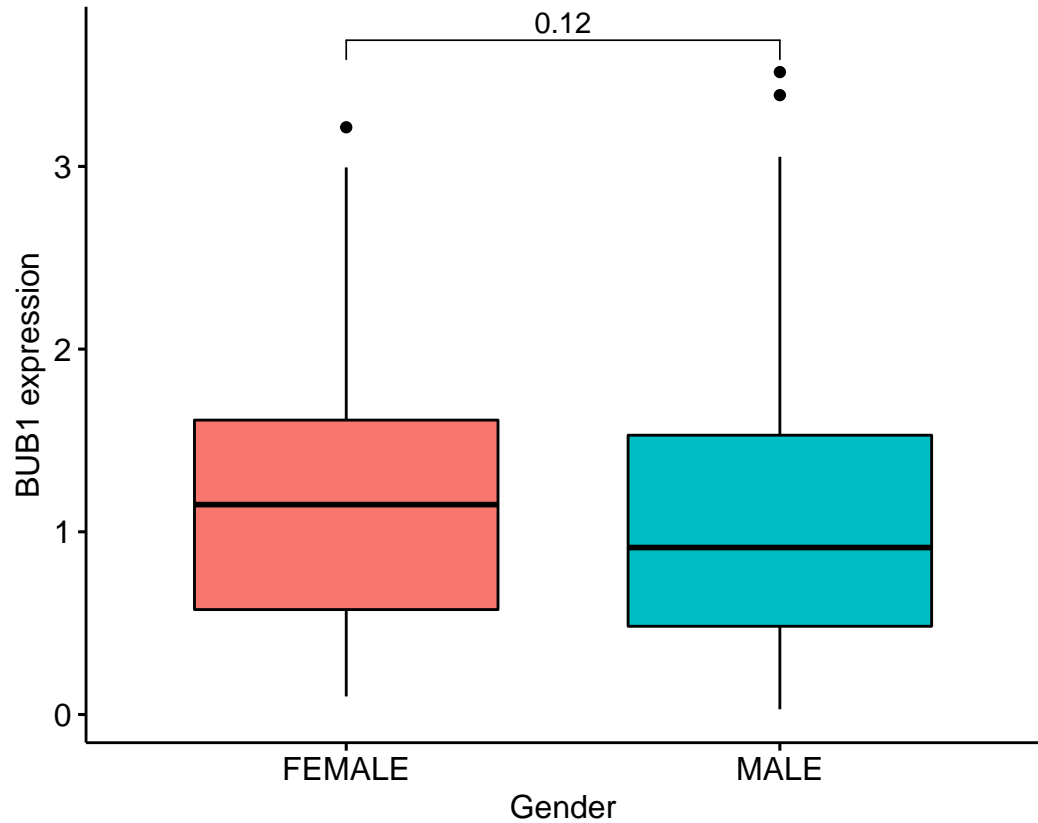

Supplement: Supplementary file 1 [file DataSheet1.zip › BUB1/Gender.pdf]

Grade G1 G2 G3 G4

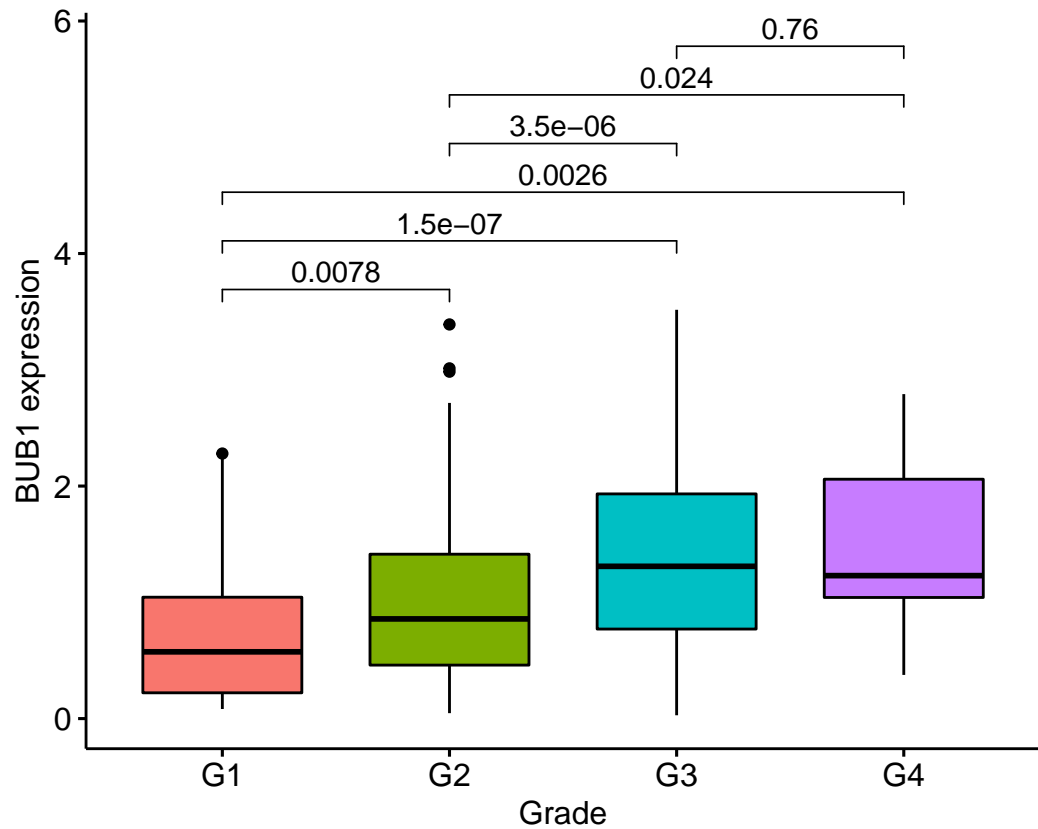

Supplement: Supplementary file 1 [file DataSheet1.zip › BUB1/Grade.pdf]

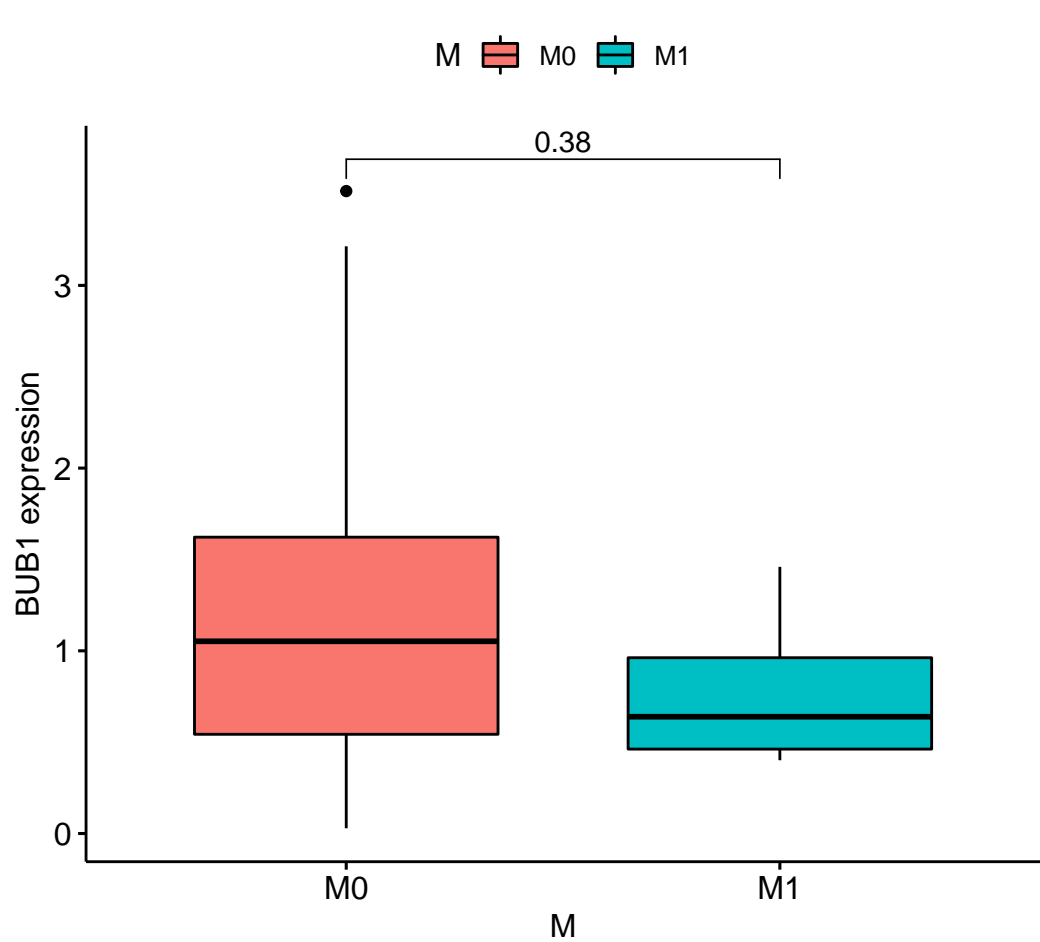

Supplement: Supplementary file 1 [file DataSheet1.zip › BUB1/M.pdf]

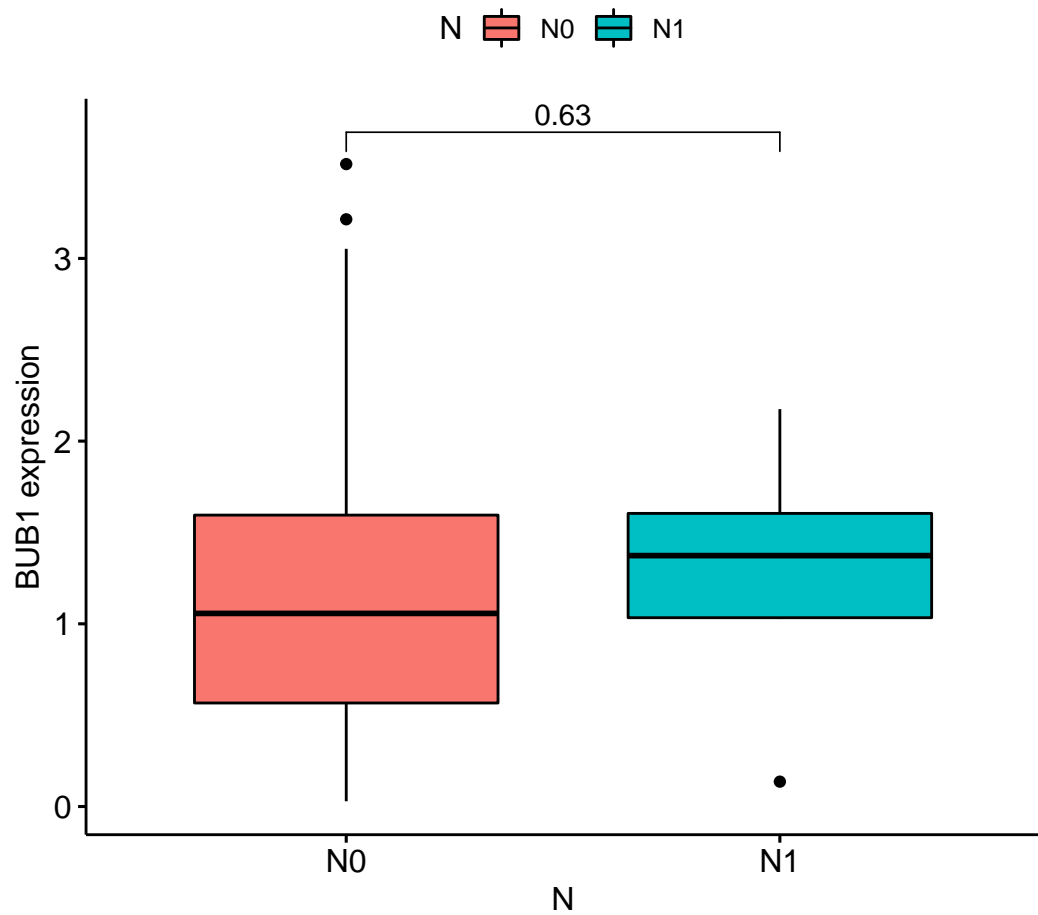

Supplement: Supplementary file 1 [file DataSheet1.zip › BUB1/N.pdf]

Stage I Stage II Stage III Stage IV

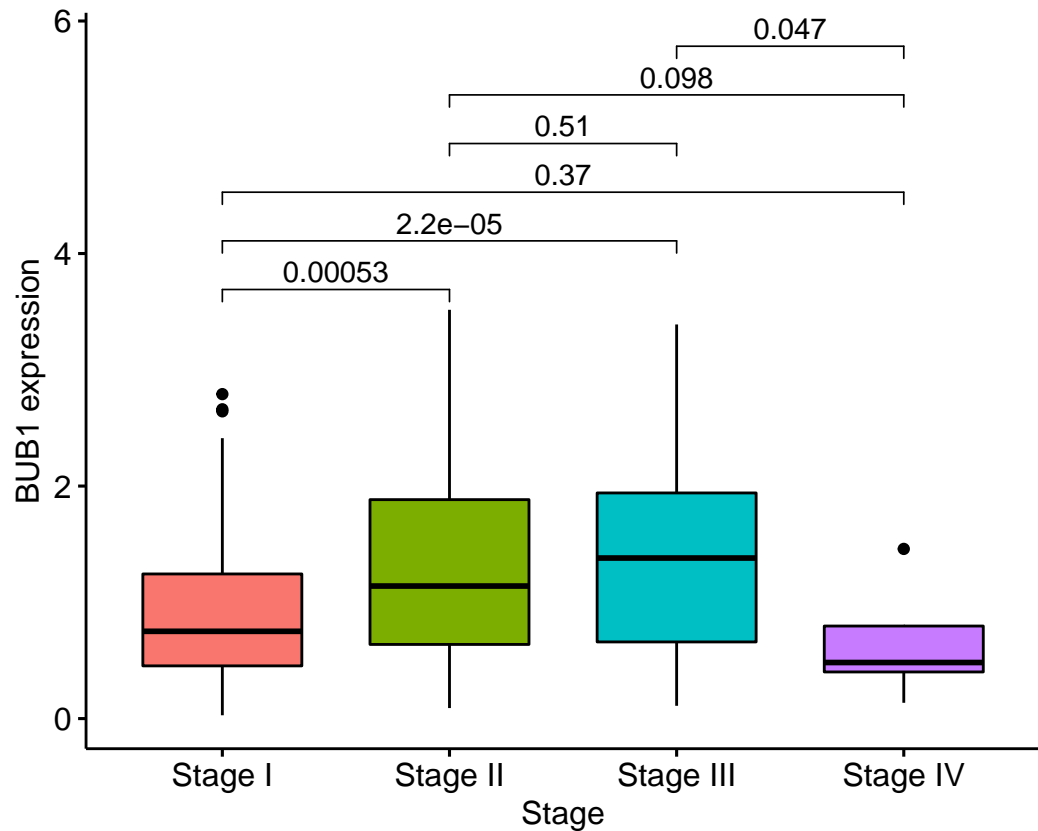

Supplement: Supplementary file 1 [file DataSheet1.zip › BUB1/Stage.pdf]

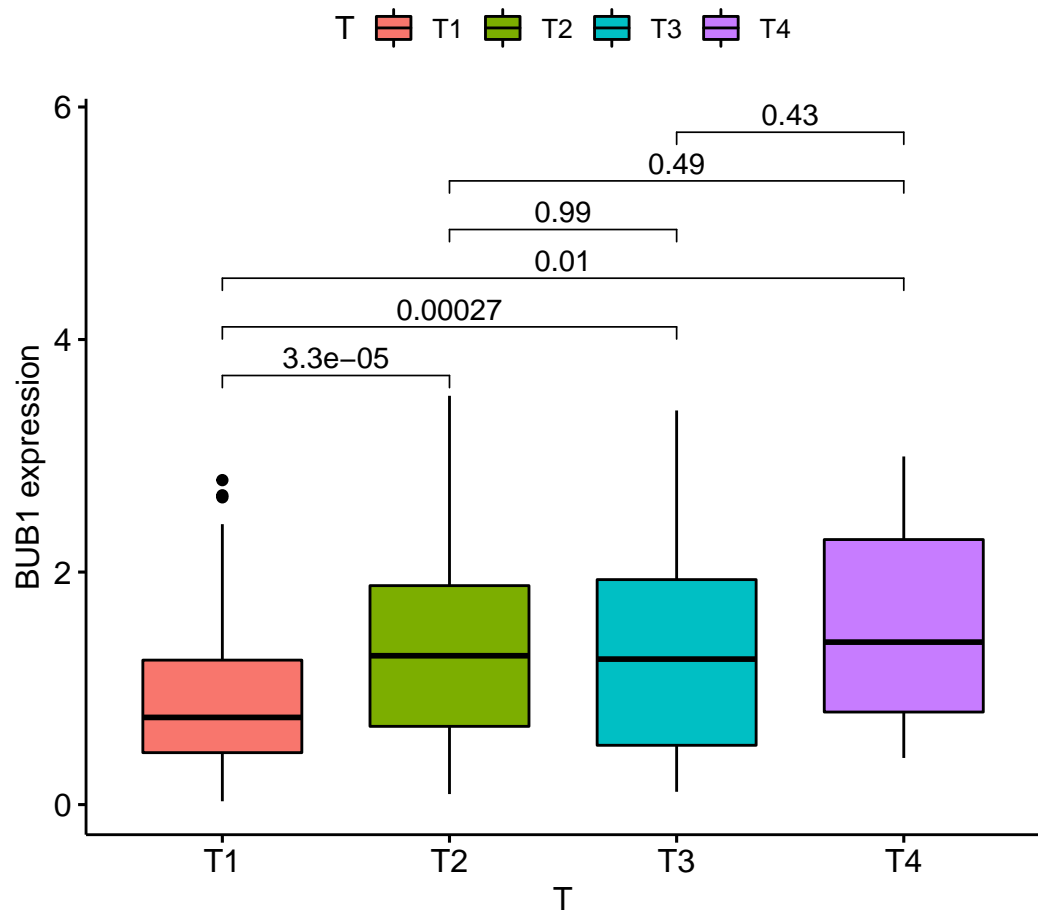

Supplement: Supplementary file 1 [file DataSheet1.zip › BUB1/T.pdf]

Age 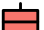 <=65 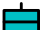 >65

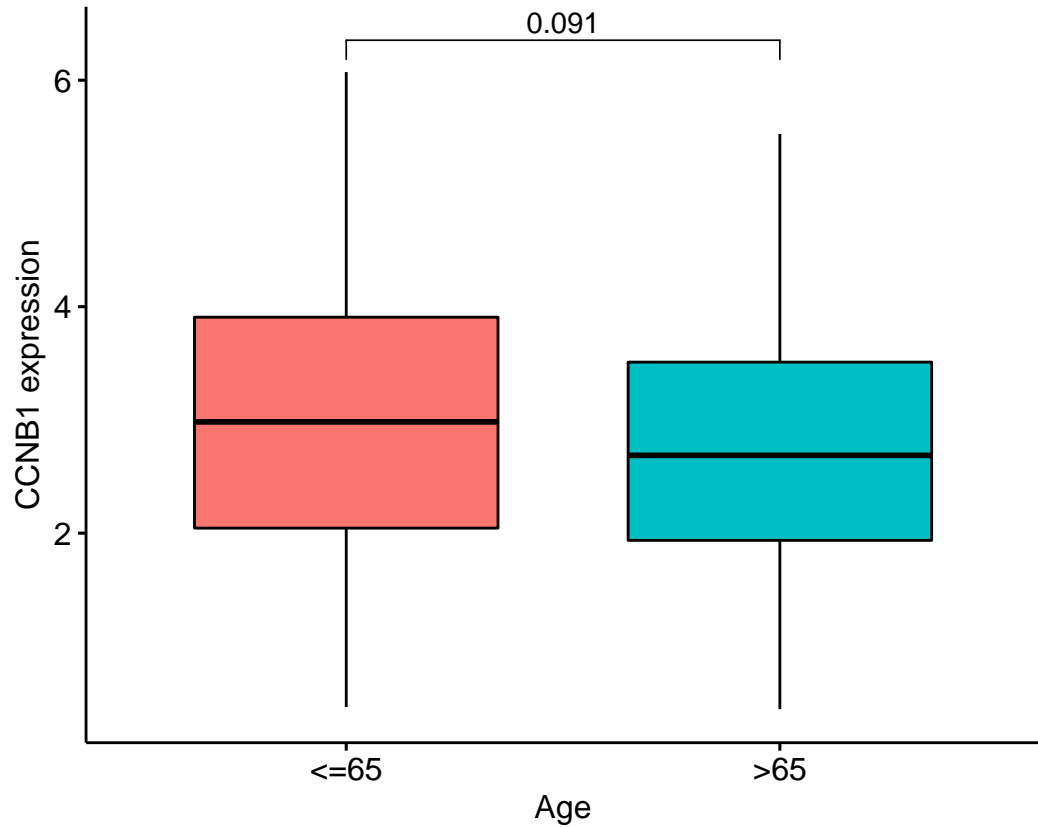

Supplement: Supplementary file 1 [file DataSheet1.zip › CCNB1/Age.pdf]

Gender 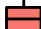 FEMALE 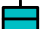 MALE

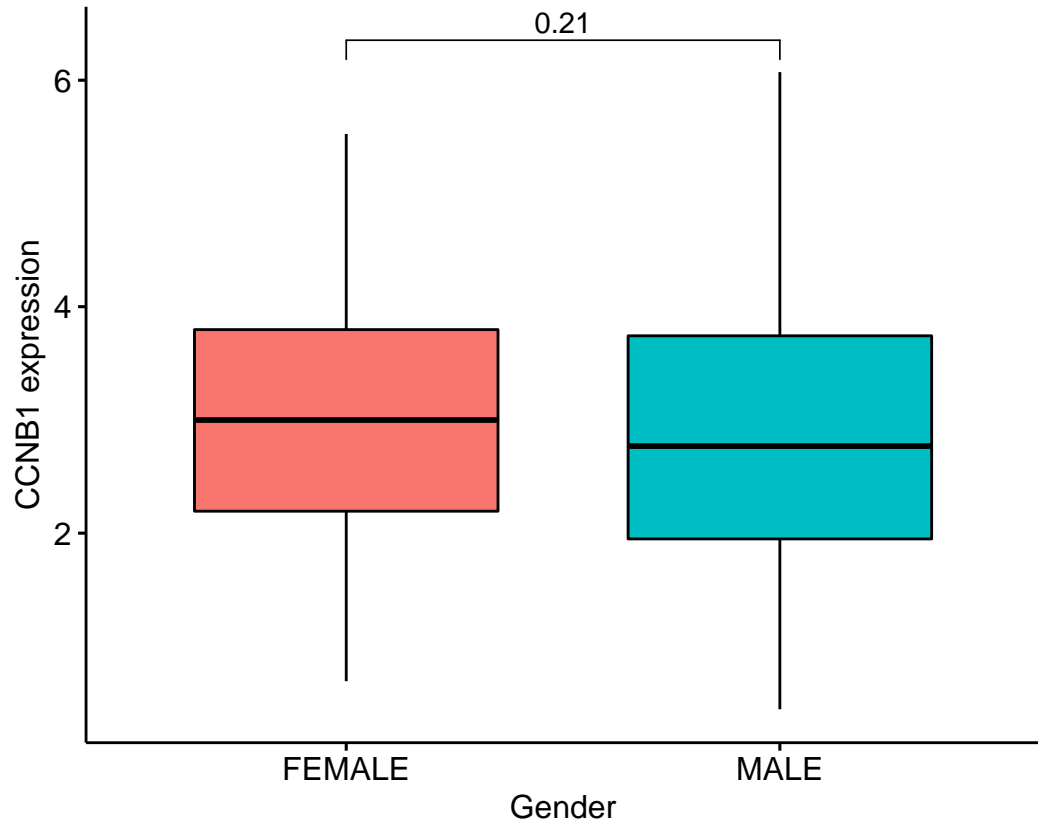

Supplement: Supplementary file 1 [file DataSheet1.zip › CCNB1/Gender.pdf]

Grade G1 G2 G3 G4

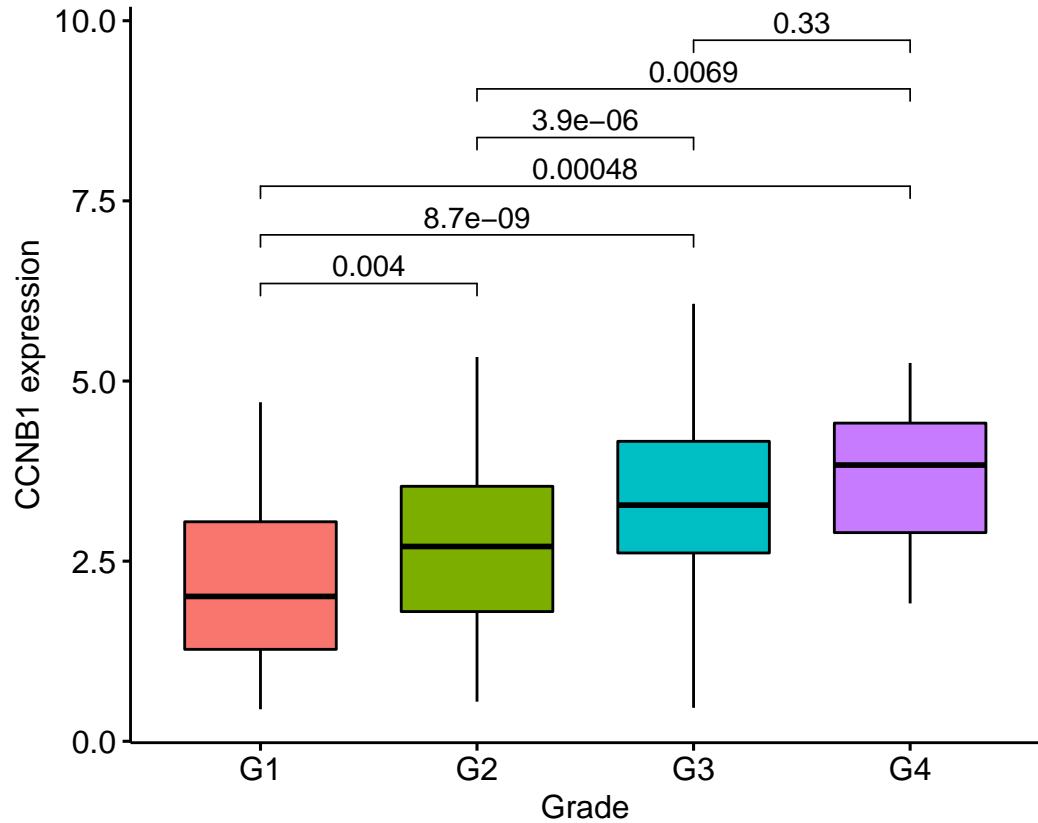

Supplement: Supplementary file 1 [file DataSheet1.zip › CCNB1/Grade.pdf]

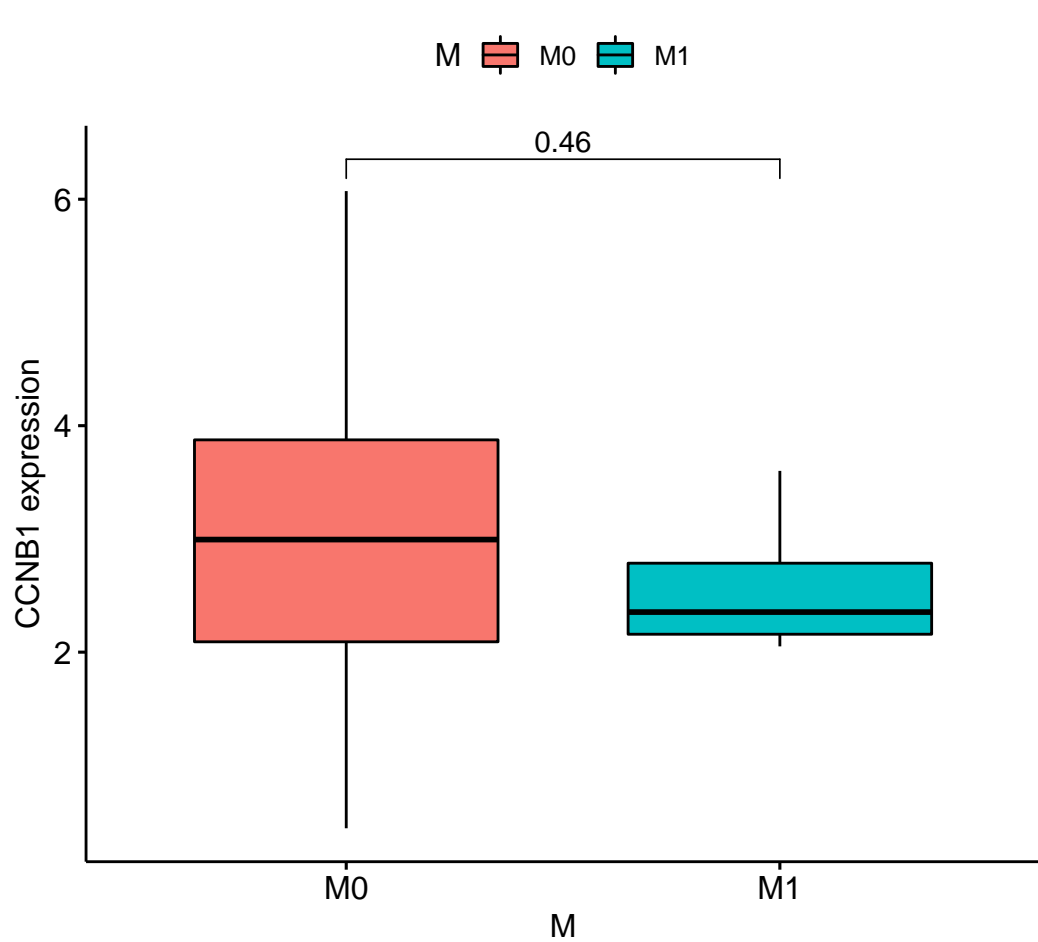

Supplement: Supplementary file 1 [file DataSheet1.zip › CCNB1/M.pdf]

CCNB1 expression

N 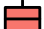 N0 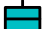 N1

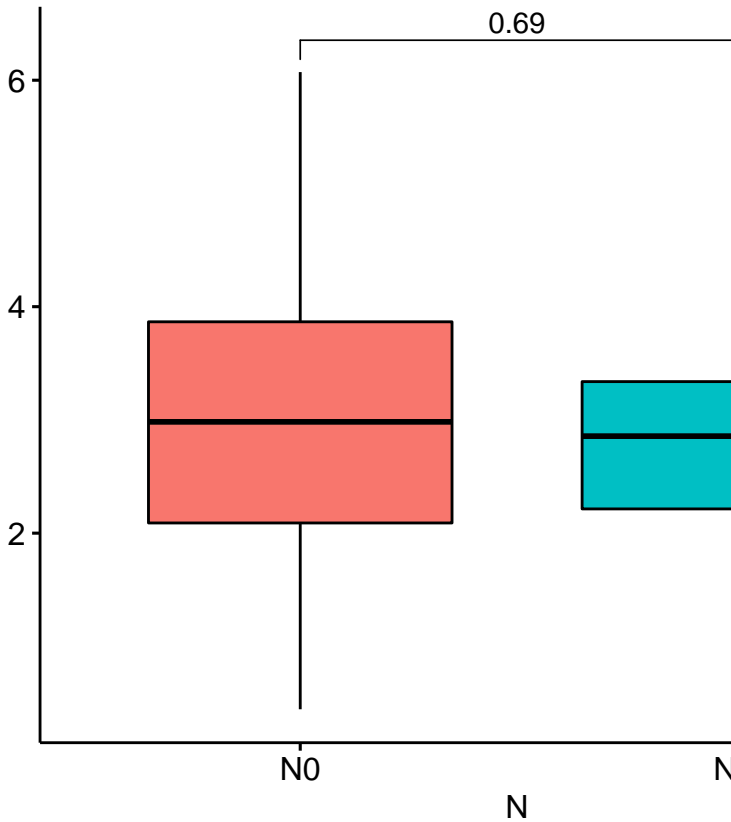

Supplement: Supplementary file 1 [file DataSheet1.zip › CCNB1/N.pdf]

Stage I Stage II Stage III Stage IV

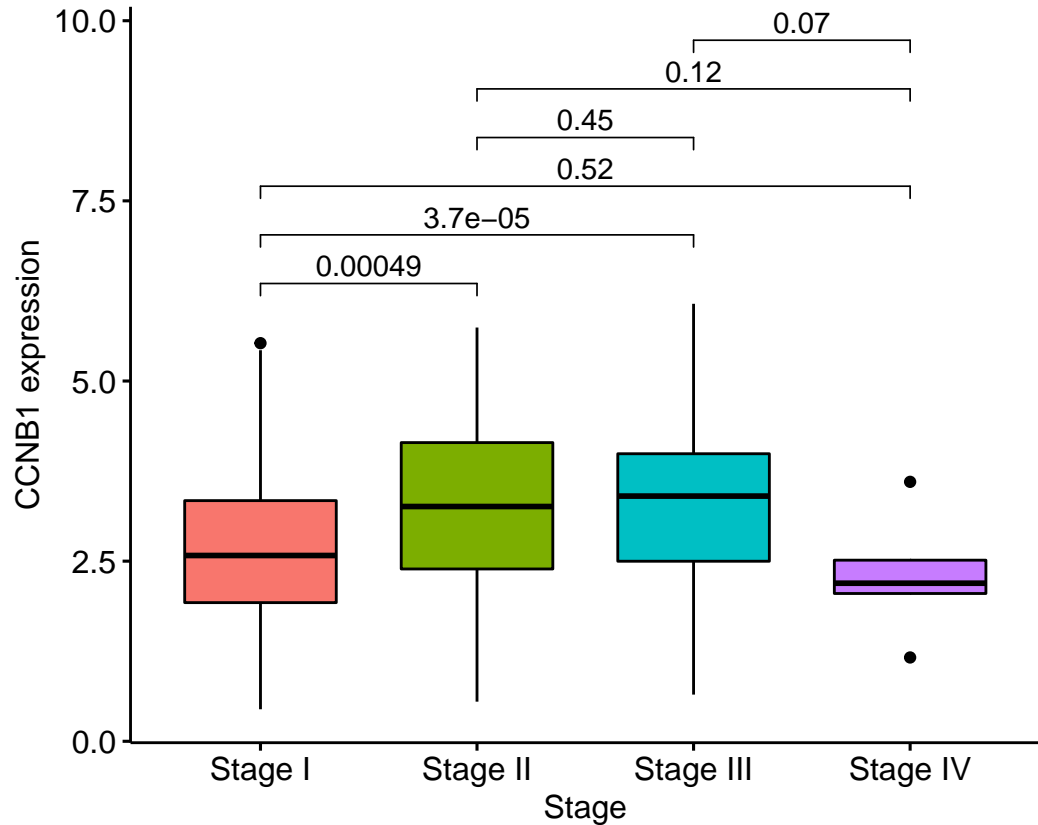

Supplement: Supplementary file 1 [file DataSheet1.zip › CCNB1/Stage.pdf]

T T1 T2 T3 T4

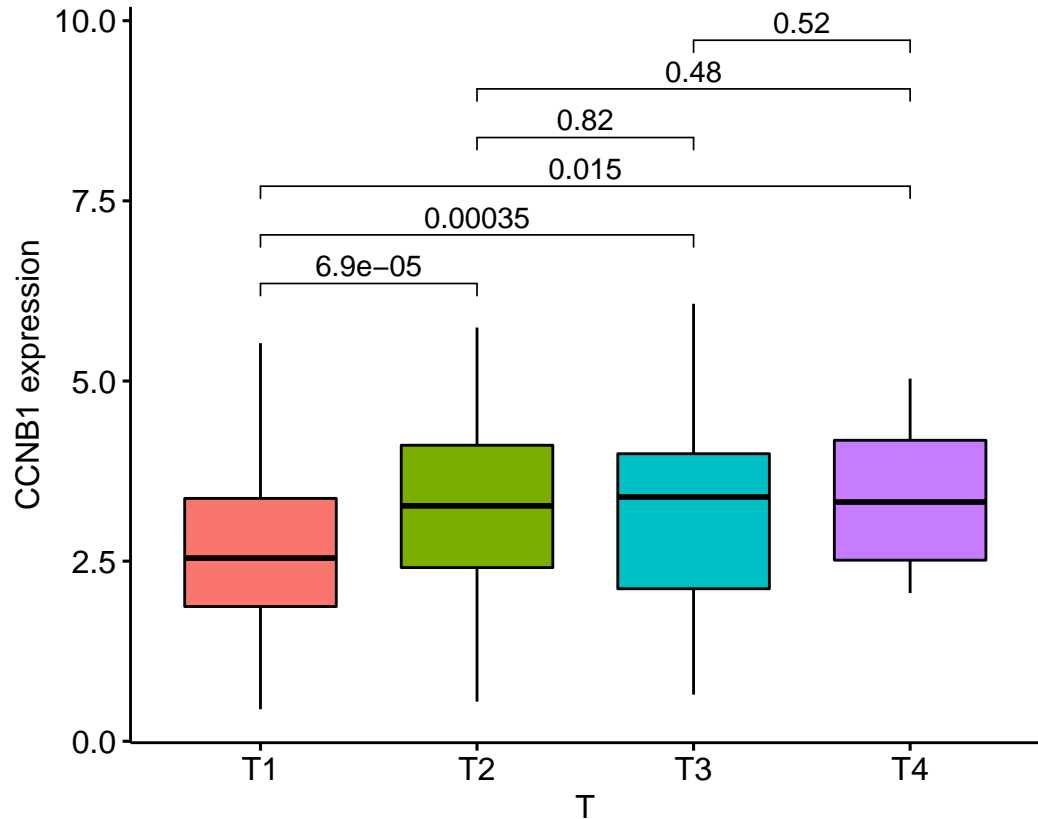

Supplement: Supplementary file 1 [file DataSheet1.zip › CCNB1/T.pdf]

Age 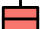 <=65 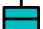 >65

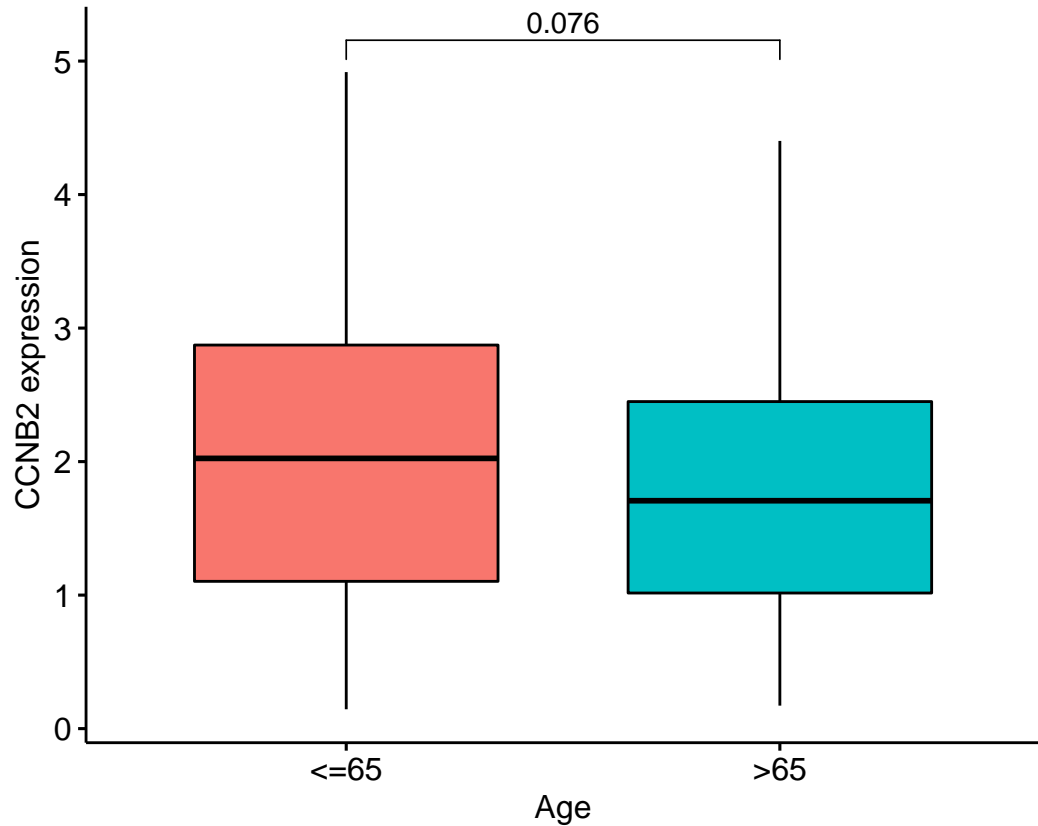

Supplement: Supplementary file 1 [file DataSheet1.zip › CCNB2/Age.pdf]

Gender 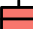 FEMALE 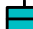 MALE

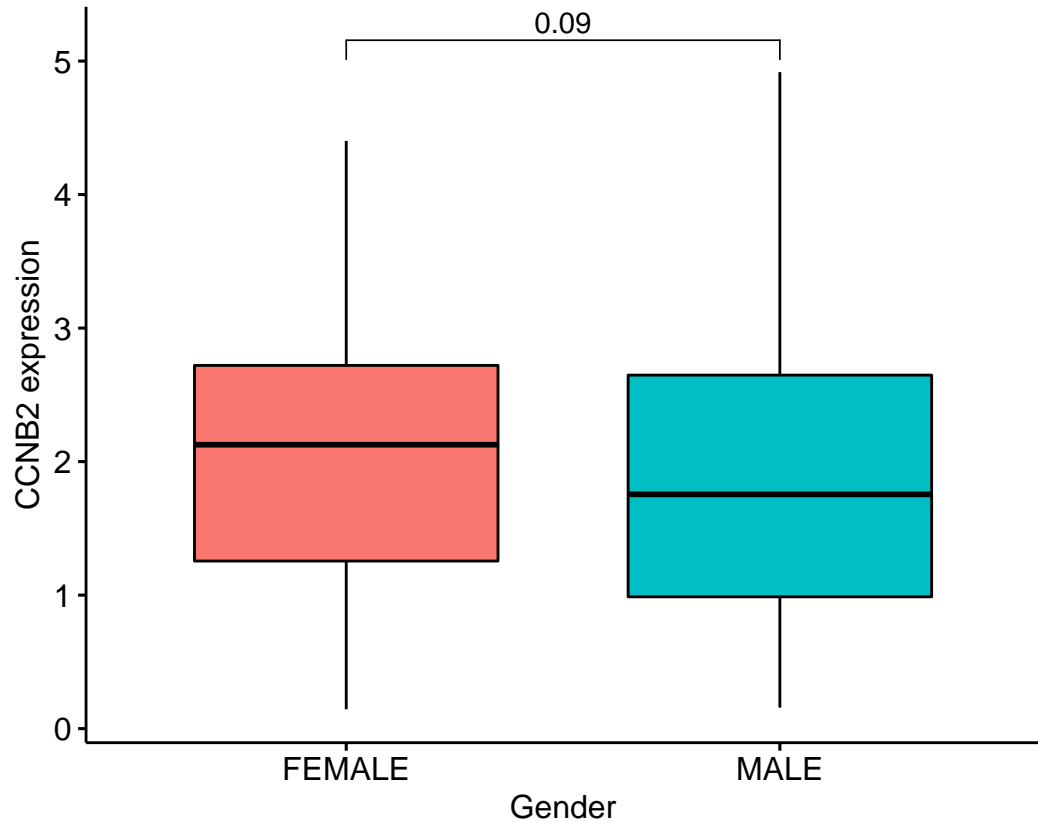

Supplement: Supplementary file 1 [file DataSheet1.zip › CCNB2/Gender.pdf]

Grade G1 G2 G3 G4

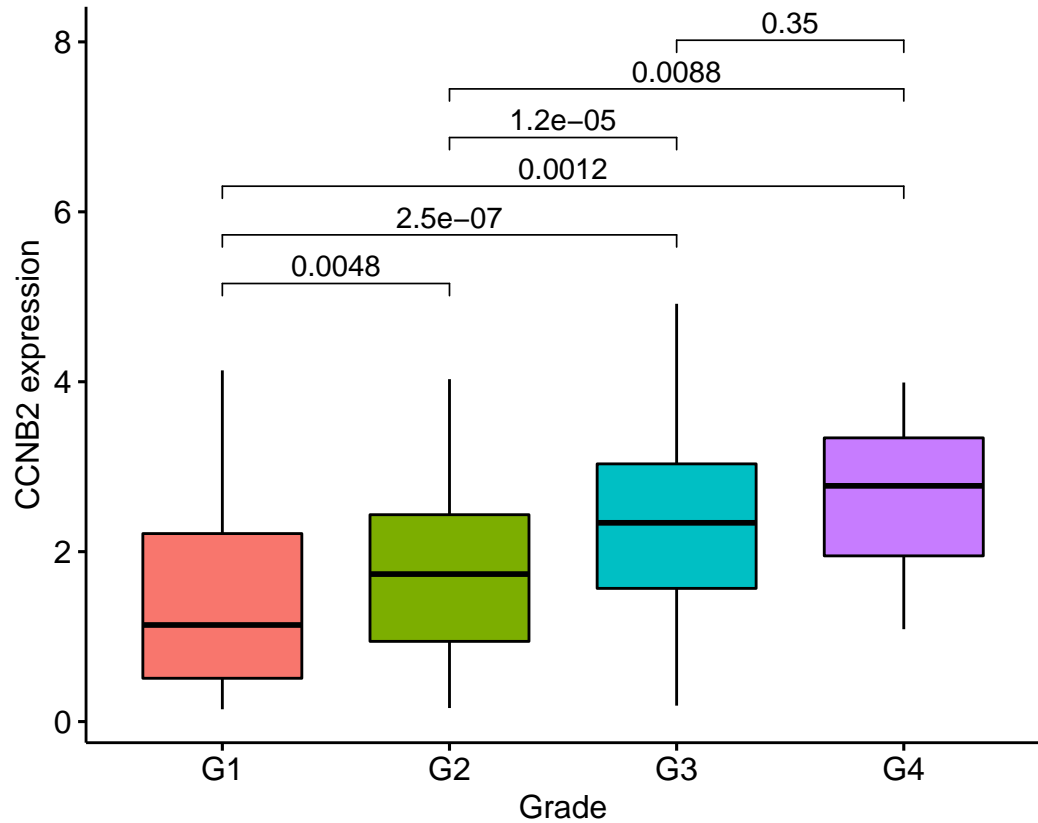

Supplement: Supplementary file 1 [file DataSheet1.zip › CCNB2/Grade.pdf]

M M0 M1

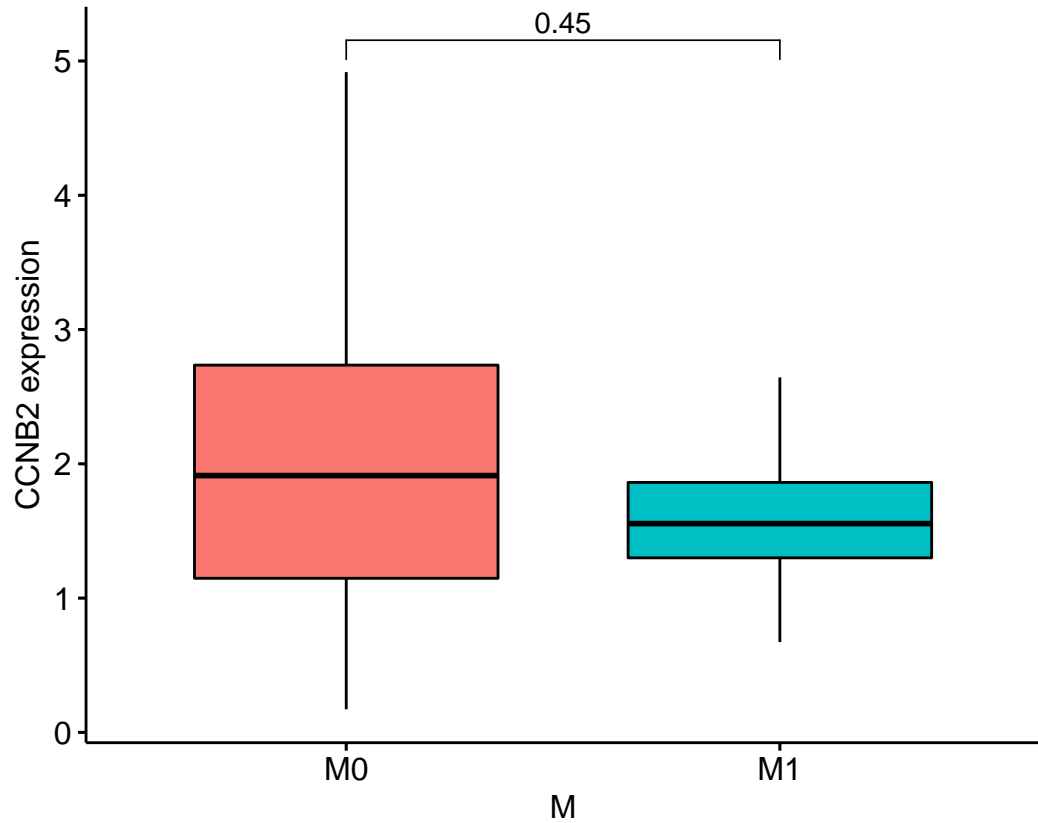

Supplement: Supplementary file 1 [file DataSheet1.zip › CCNB2/M.pdf]

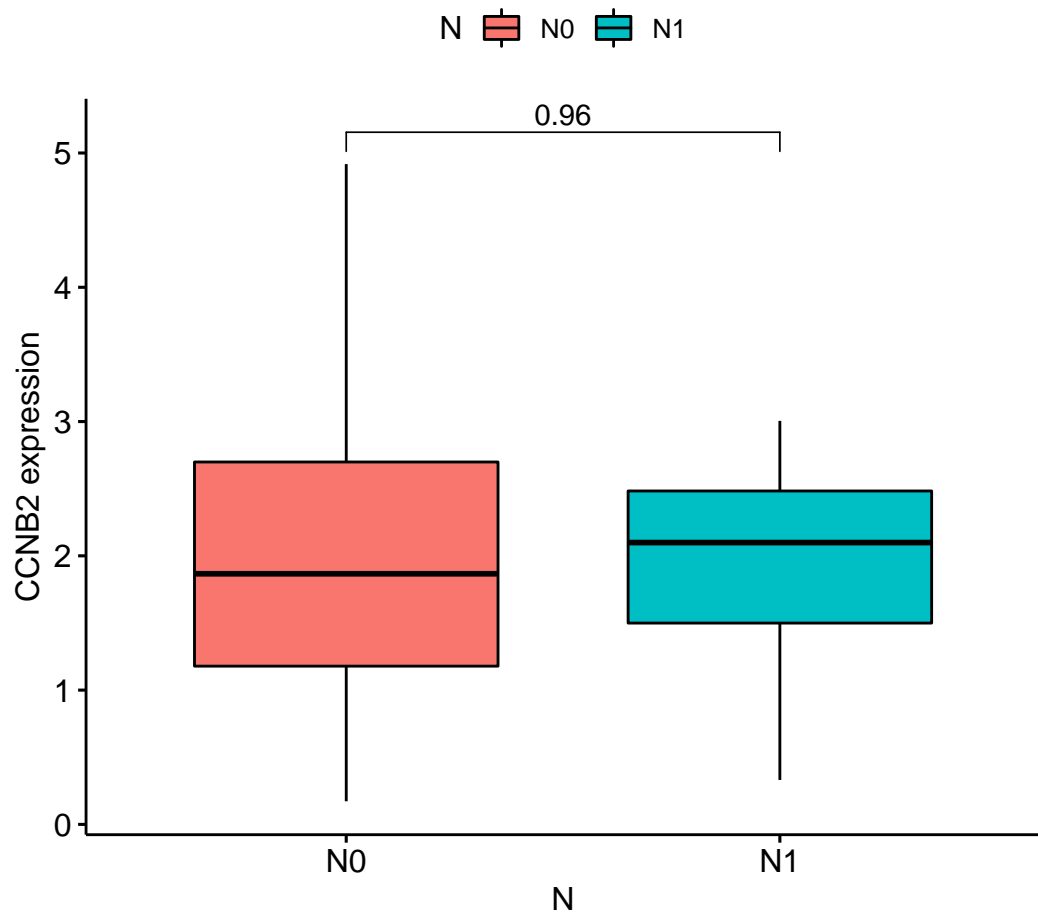

Supplement: Supplementary file 1 [file DataSheet1.zip › CCNB2/N.pdf]

Stage I Stage II Stage III Stage IV

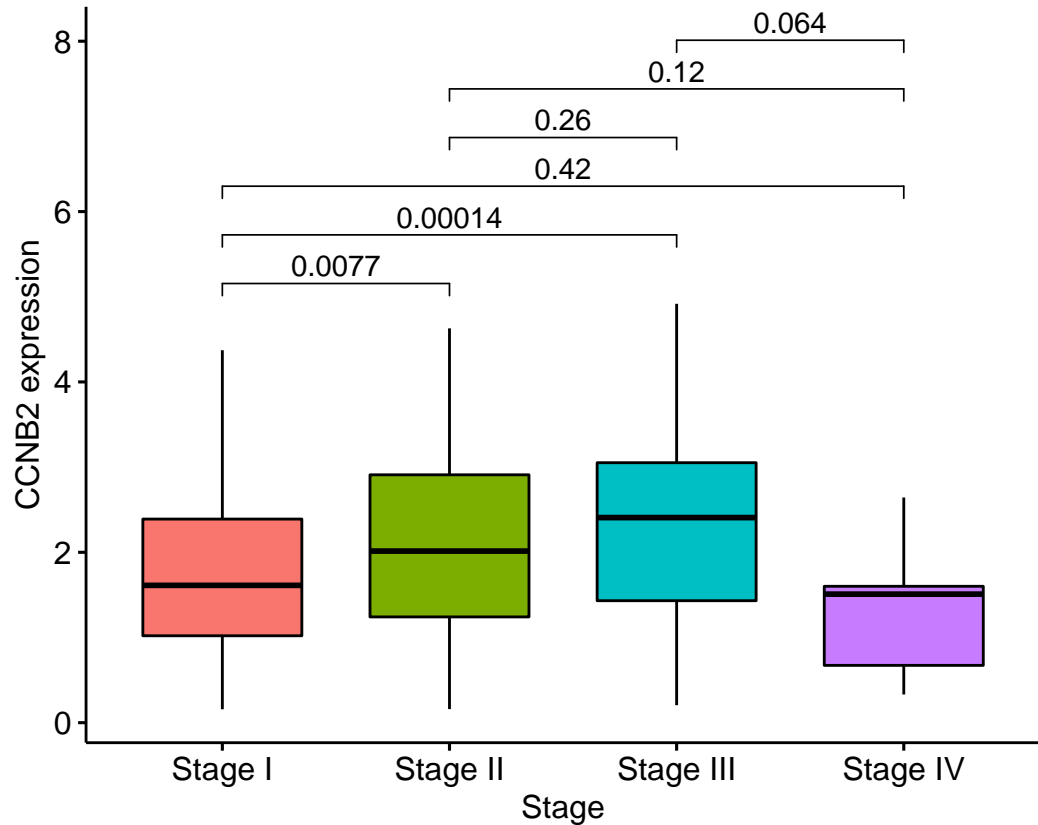

Supplement: Supplementary file 1 [file DataSheet1.zip › CCNB2/Stage.pdf]

T T1 T2 T3 T4

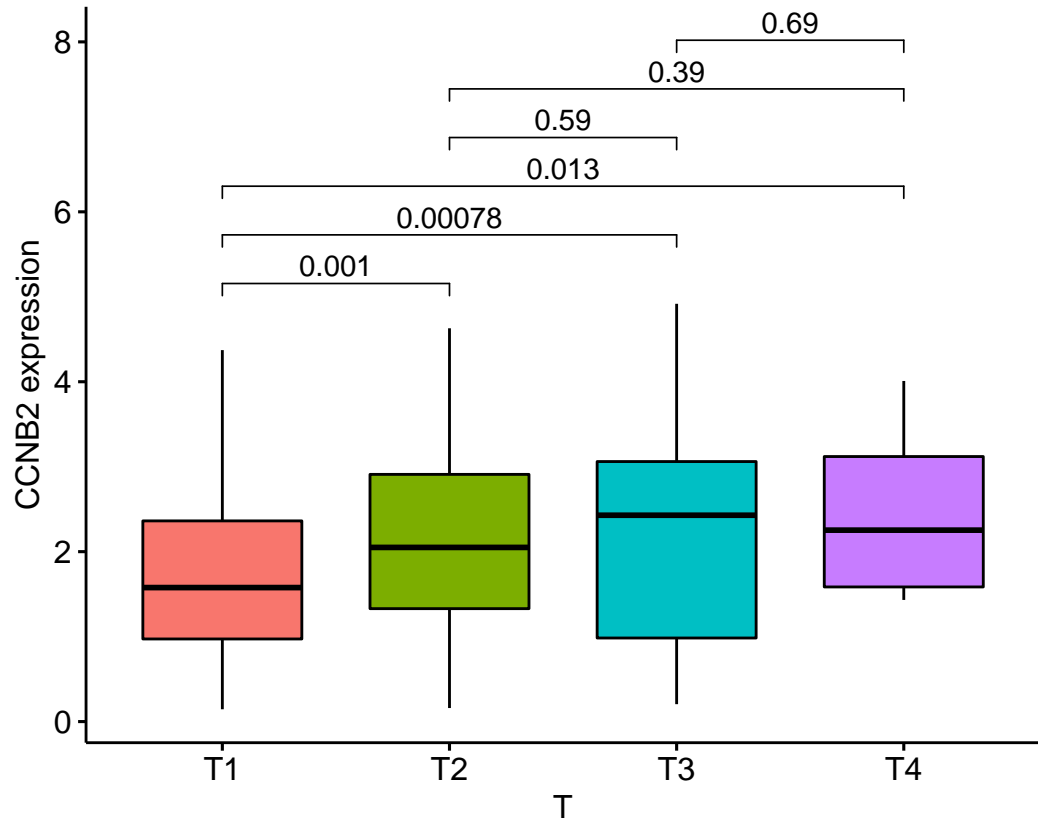

Supplement: Supplementary file 1 [file DataSheet1.zip › CCNB2/T.pdf]

Age 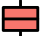 <=65 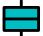 >65

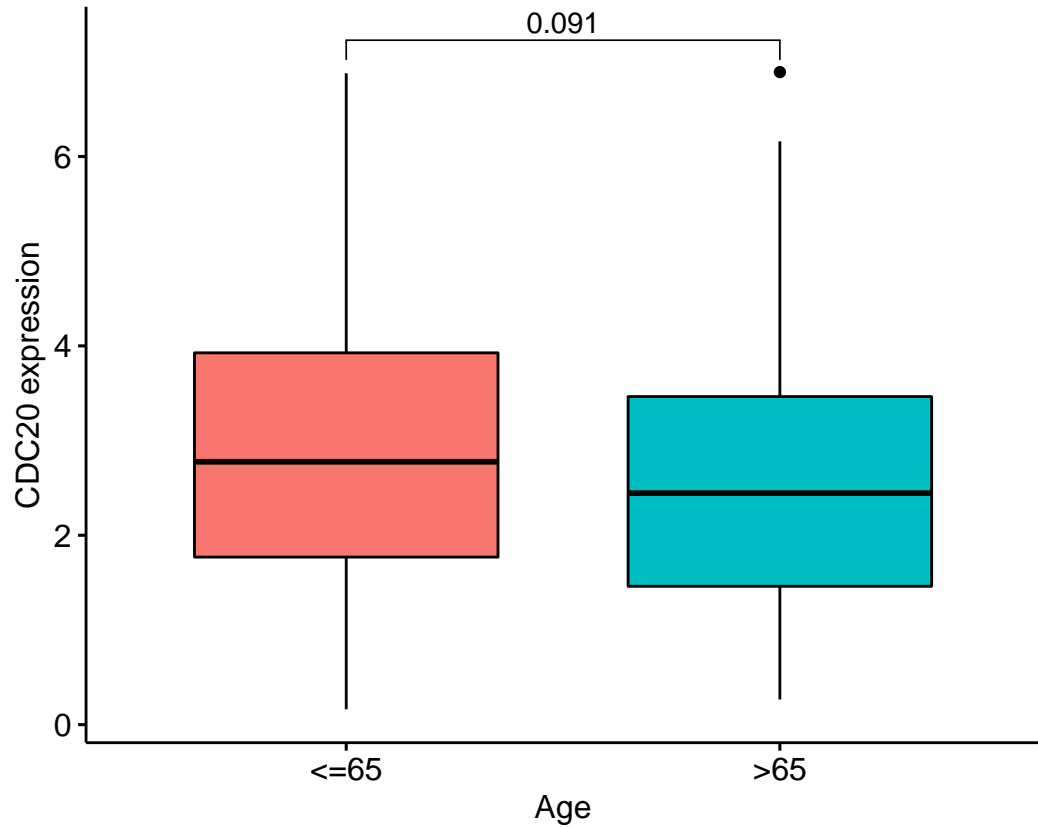

Supplement: Supplementary file 1 [file DataSheet1.zip › CDC20/Age.pdf]

Gender 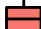 FEMALE 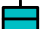 MALE

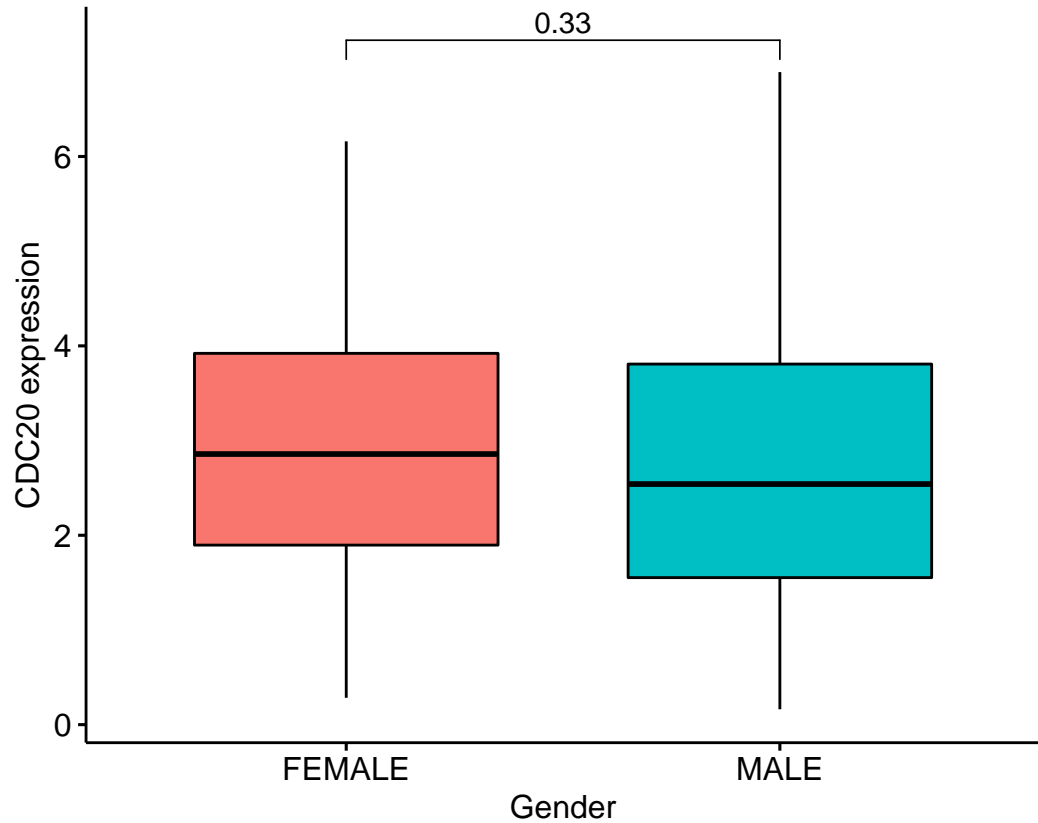

Supplement: Supplementary file 1 [file DataSheet1.zip › CDC20/Gender.pdf]

Grade G1 G2 G3 G4

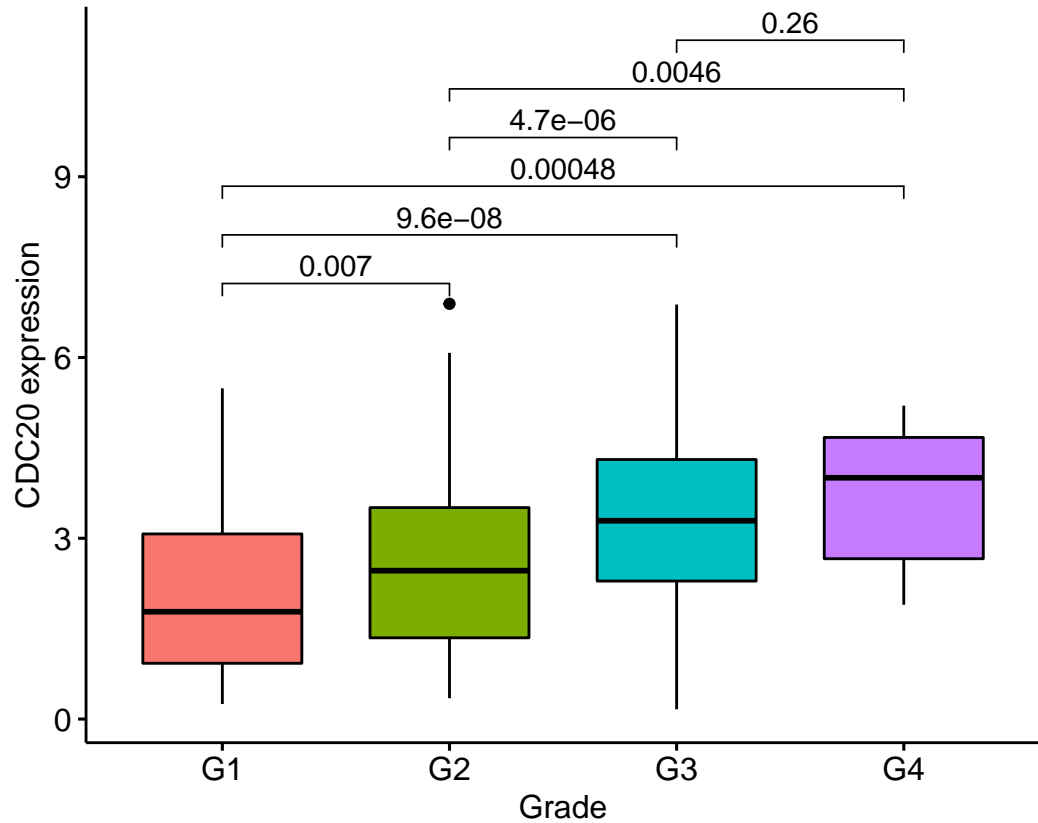

Supplement: Supplementary file 1 [file DataSheet1.zip › CDC20/Grade.pdf]

M M0 M1

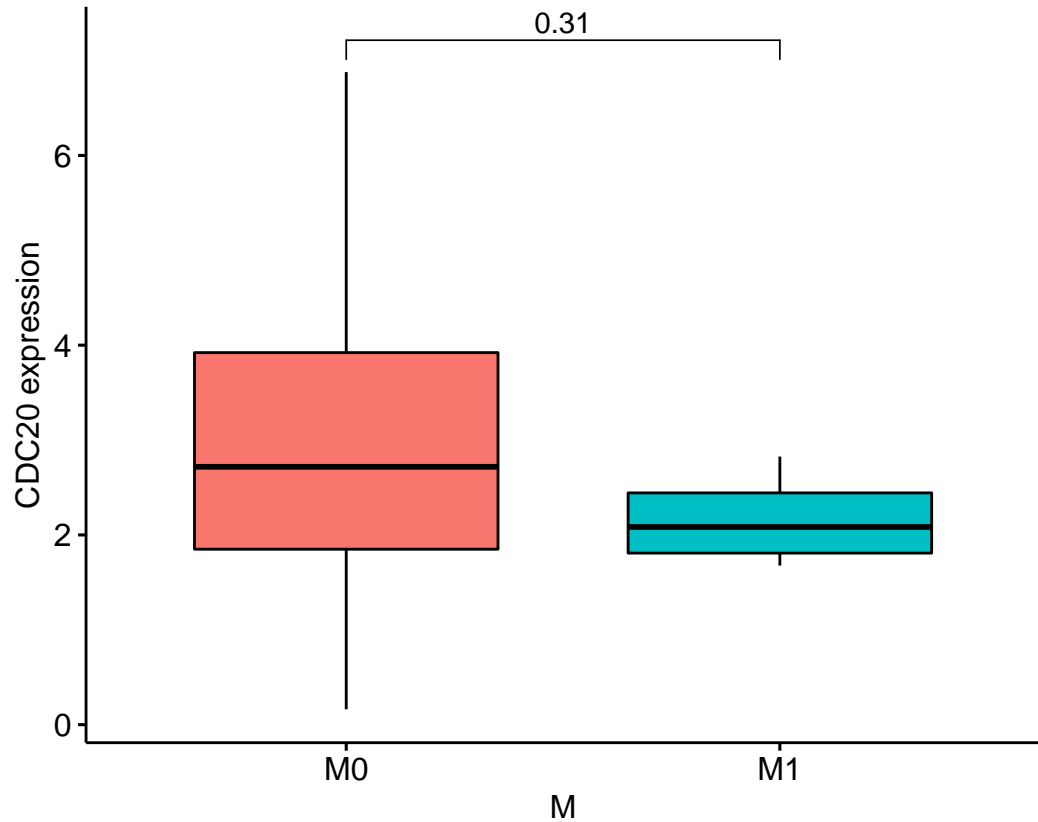

Supplement: Supplementary file 1 [file DataSheet1.zip › CDC20/M.pdf]

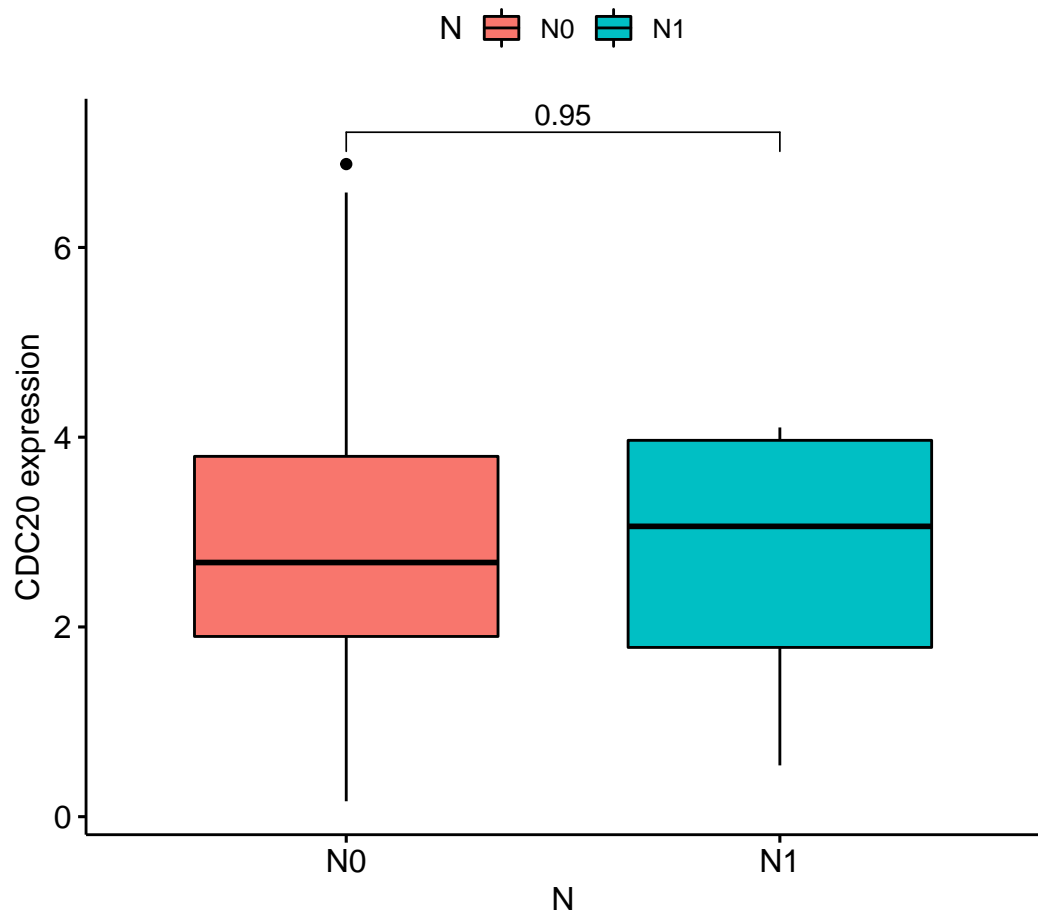

Supplement: Supplementary file 1 [file DataSheet1.zip › CDC20/N.pdf]

Stage I Stage II Stage III Stage IV

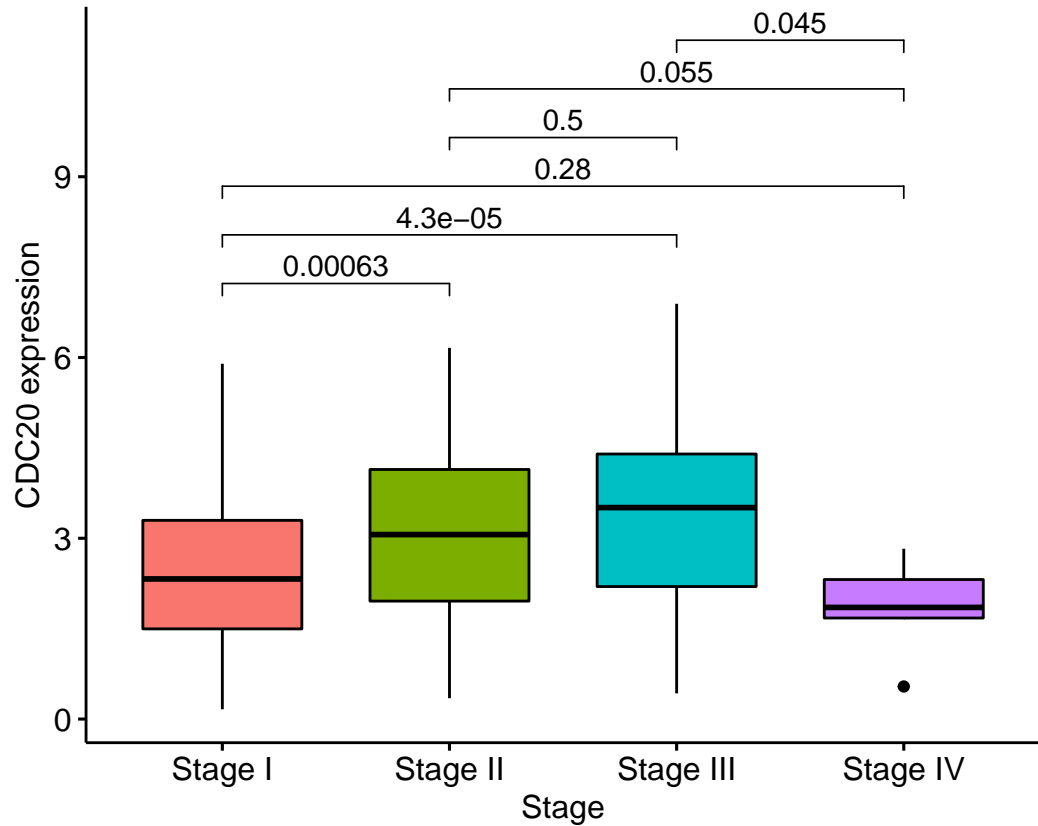

Supplement: Supplementary file 1 [file DataSheet1.zip › CDC20/Stage.pdf]

T T1 T2 T3 T4

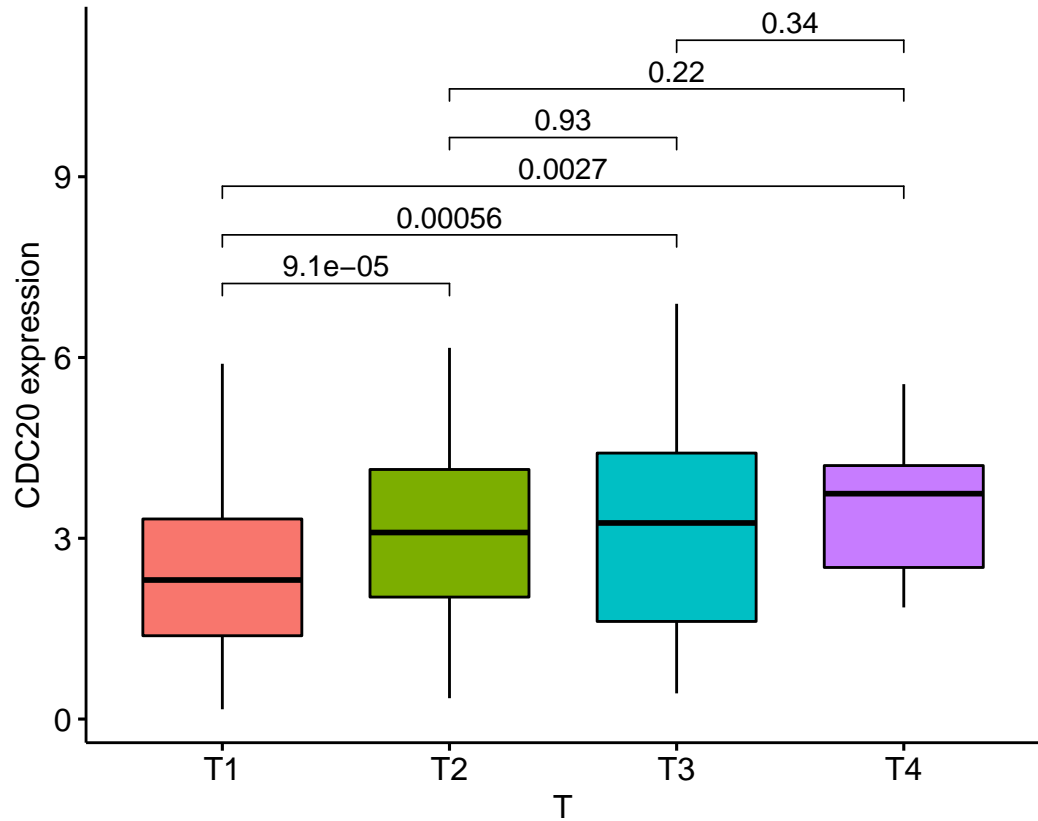

Supplement: Supplementary file 1 [file DataSheet1.zip › CDC20/T.pdf]

Age 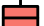 <=65 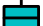 >65

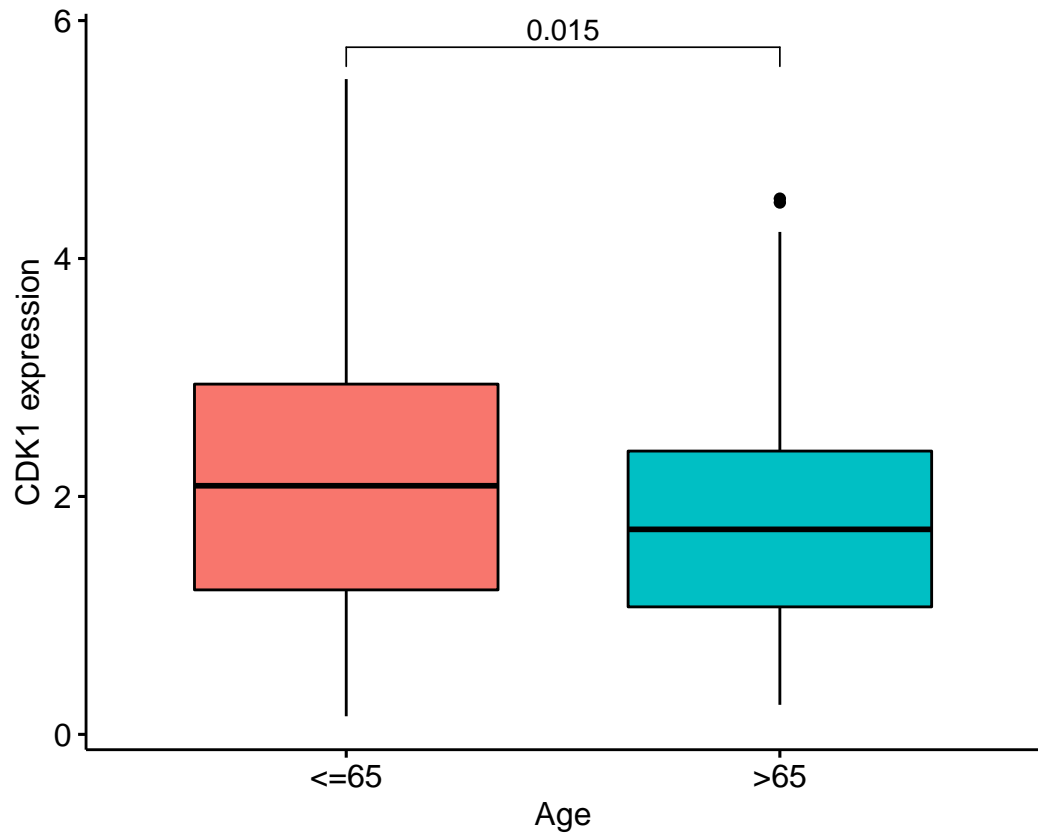

Supplement: Supplementary file 1 [file DataSheet1.zip › CDK1/Age.pdf]

Gender 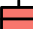 FEMALE 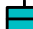 MALE

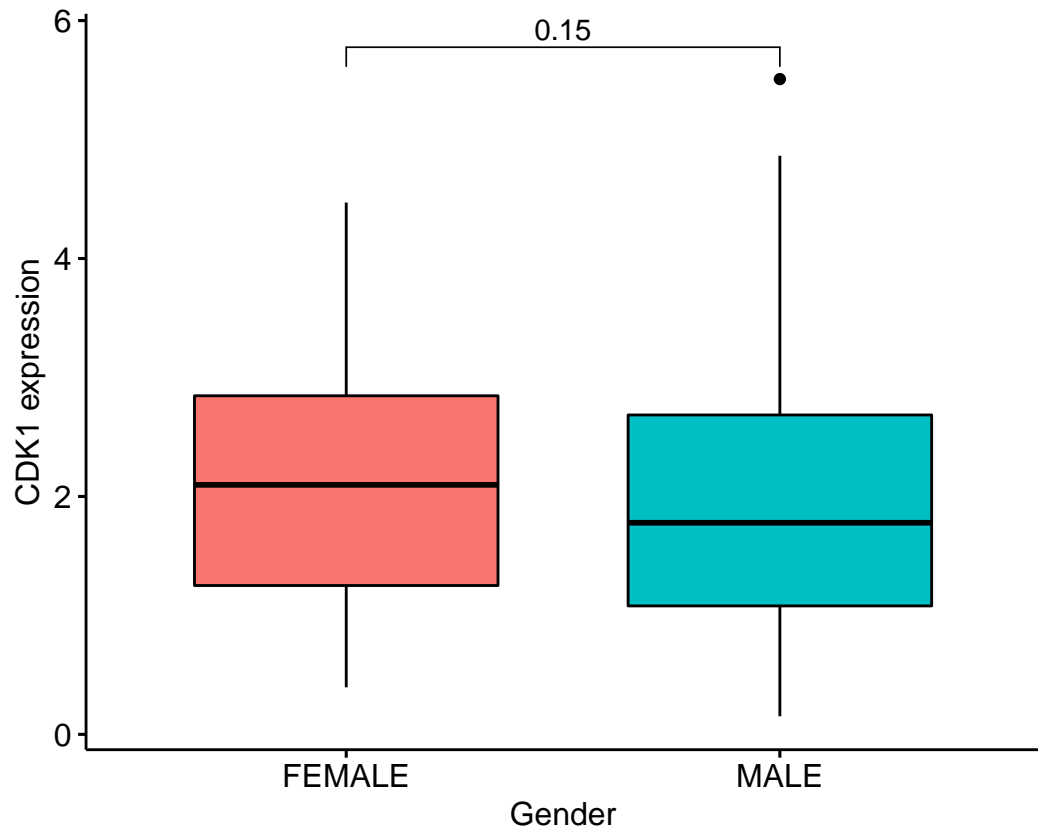

Supplement: Supplementary file 1 [file DataSheet1.zip › CDK1/Gender.pdf]

Grade 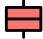 G1 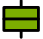 G2 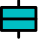 G3 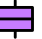 G4

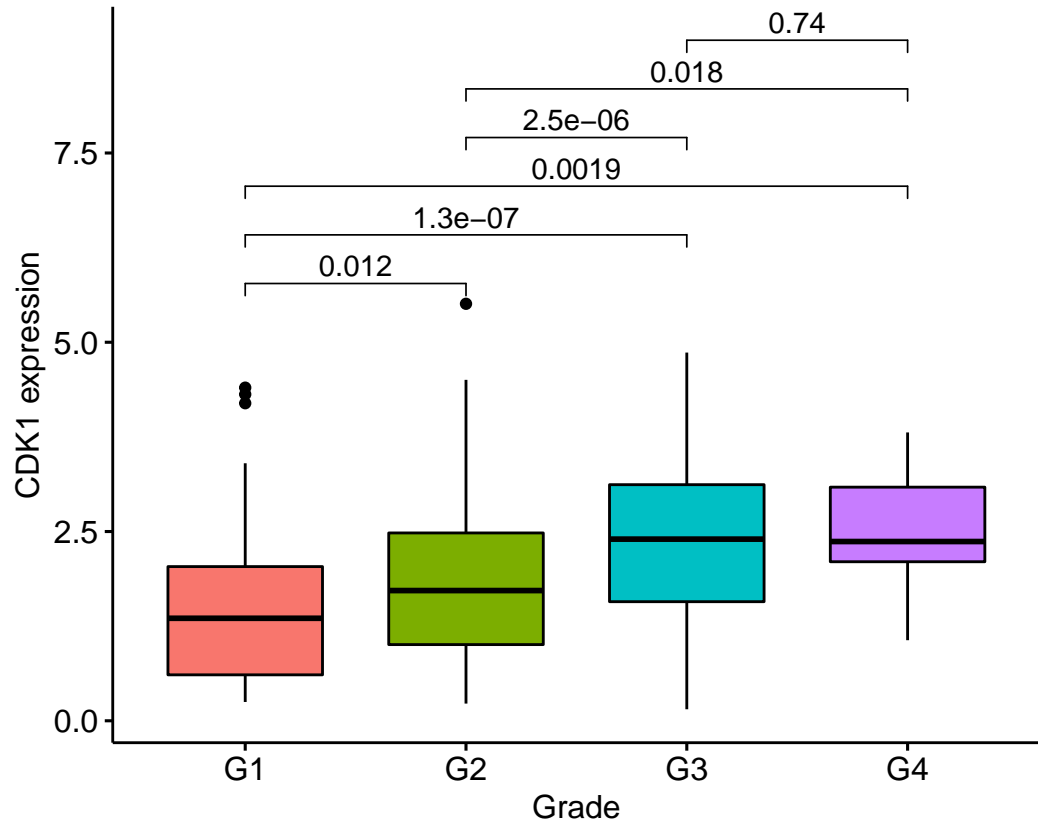

Supplement: Supplementary file 1 [file DataSheet1.zip › CDK1/Grade.pdf]

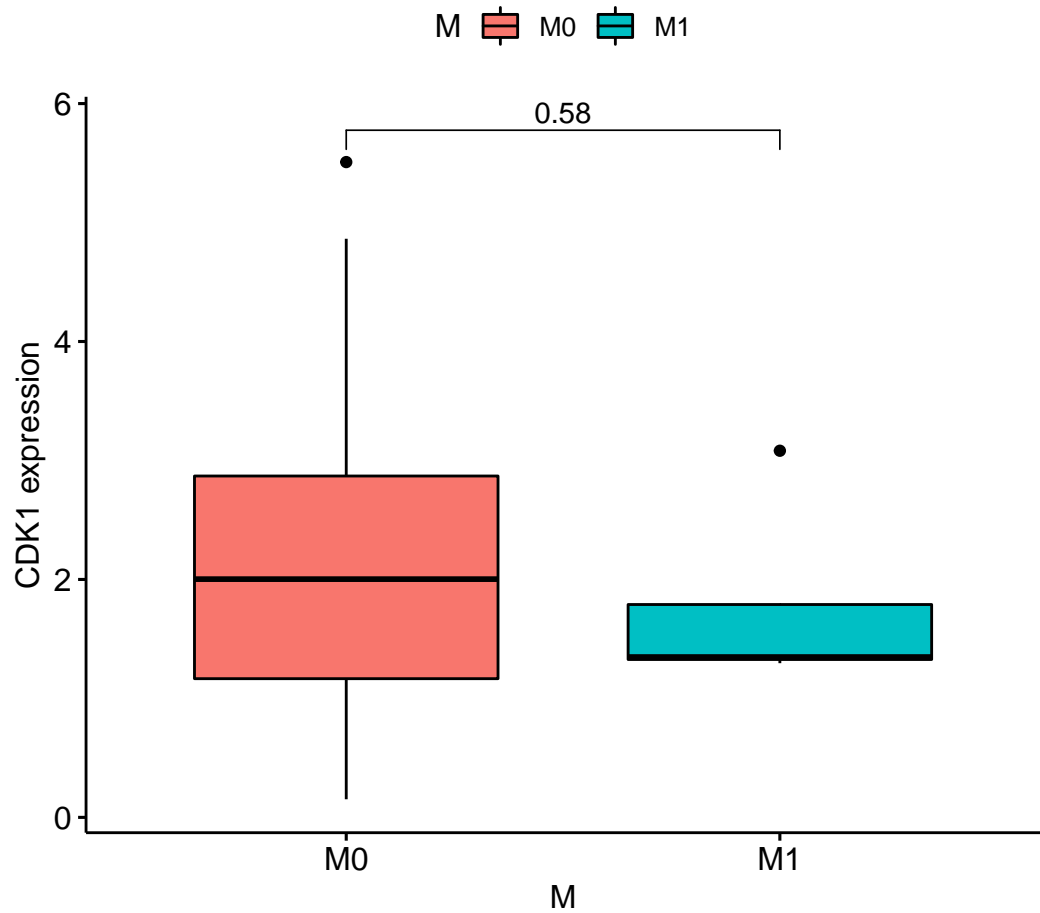

Supplement: Supplementary file 1 [file DataSheet1.zip › CDK1/M.pdf]

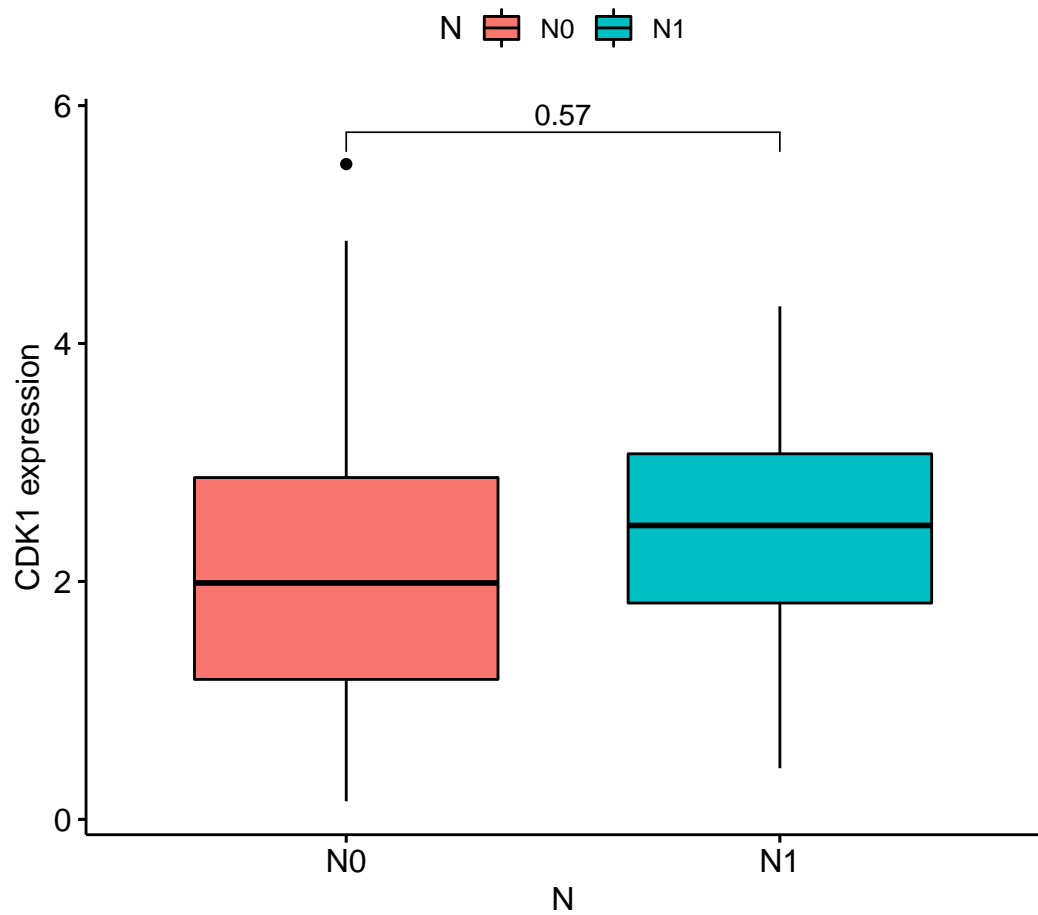

Supplement: Supplementary file 1 [file DataSheet1.zip › CDK1/N.pdf]

Stage 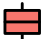 Stage I 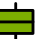 Stage II 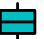 Stage III 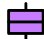 Stage IV

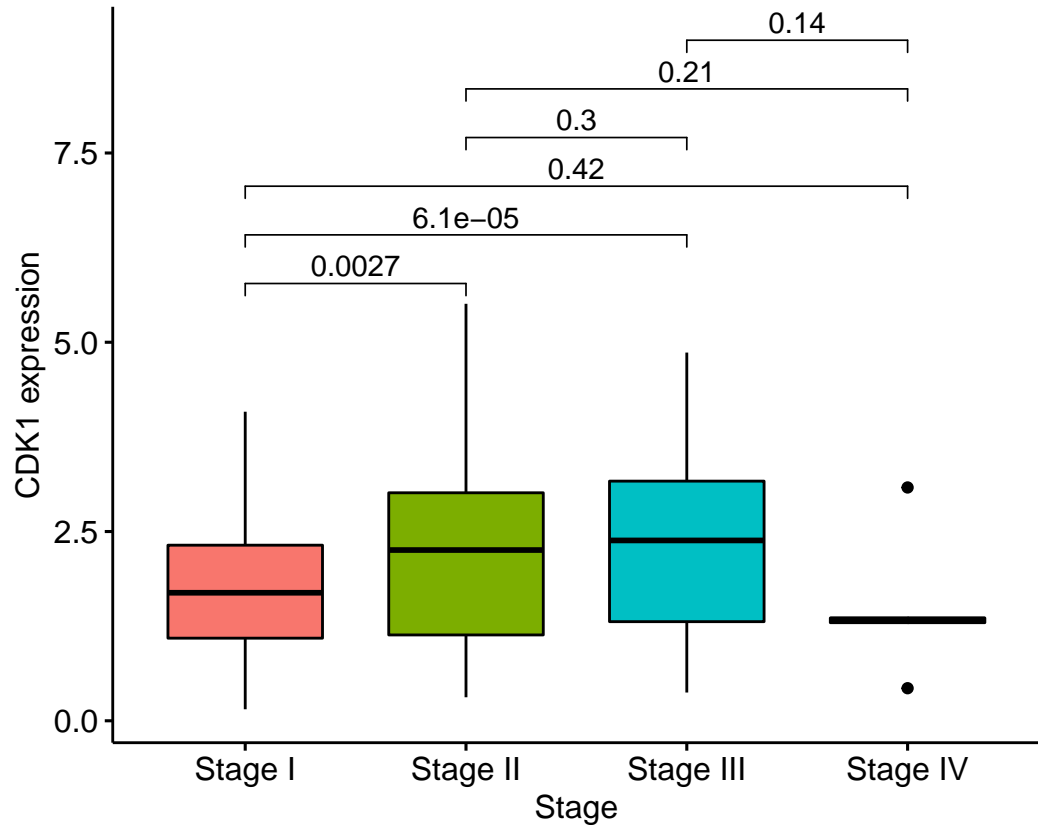

Supplement: Supplementary file 1 [file DataSheet1.zip › CDK1/Stage.pdf]

T T1 T2 T3 T4

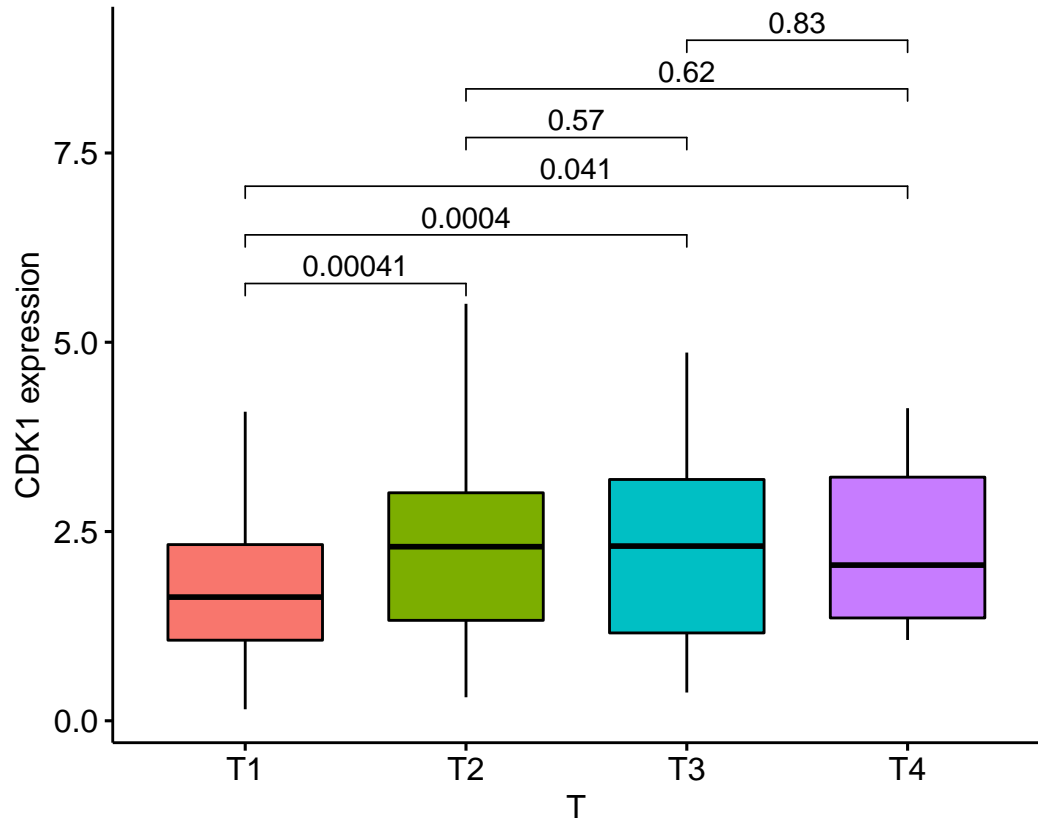

Supplement: Supplementary file 1 [file DataSheet1.zip › CDK1/T.pdf]

Age 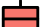 <=65 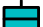 >65

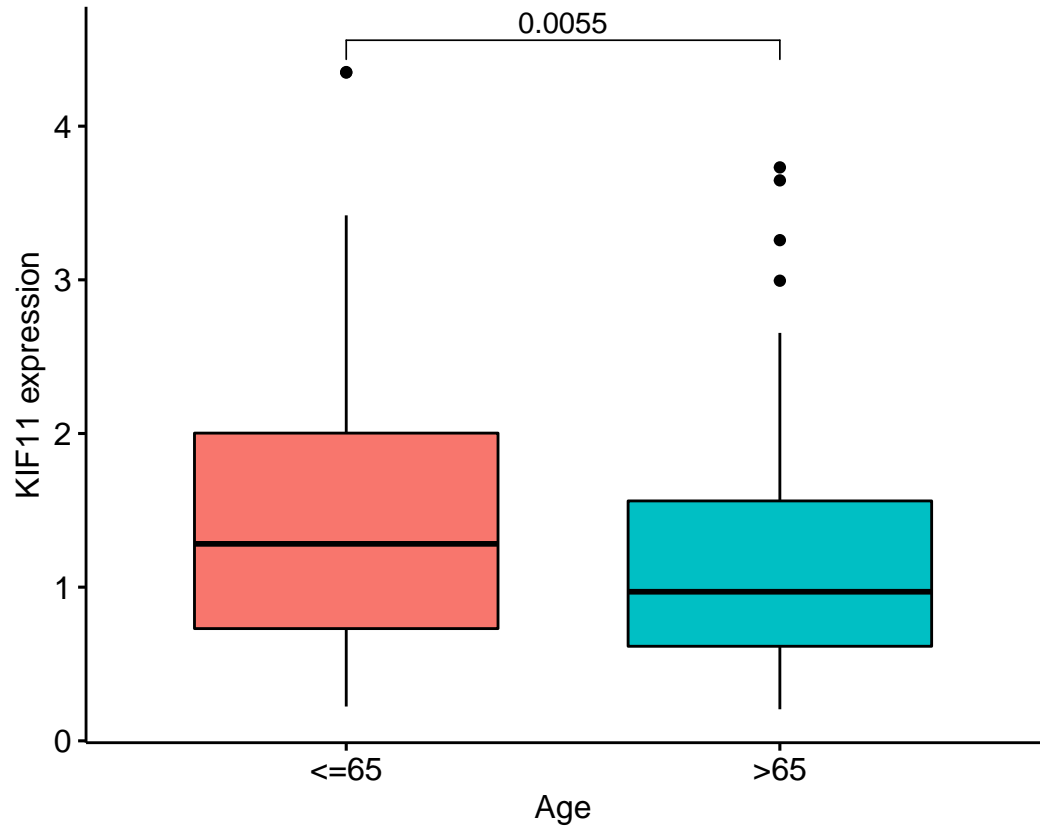

Supplement: Supplementary file 1 [file DataSheet1.zip › KIF11/Age.pdf]

Gender 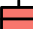 FEMALE 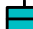 MALE

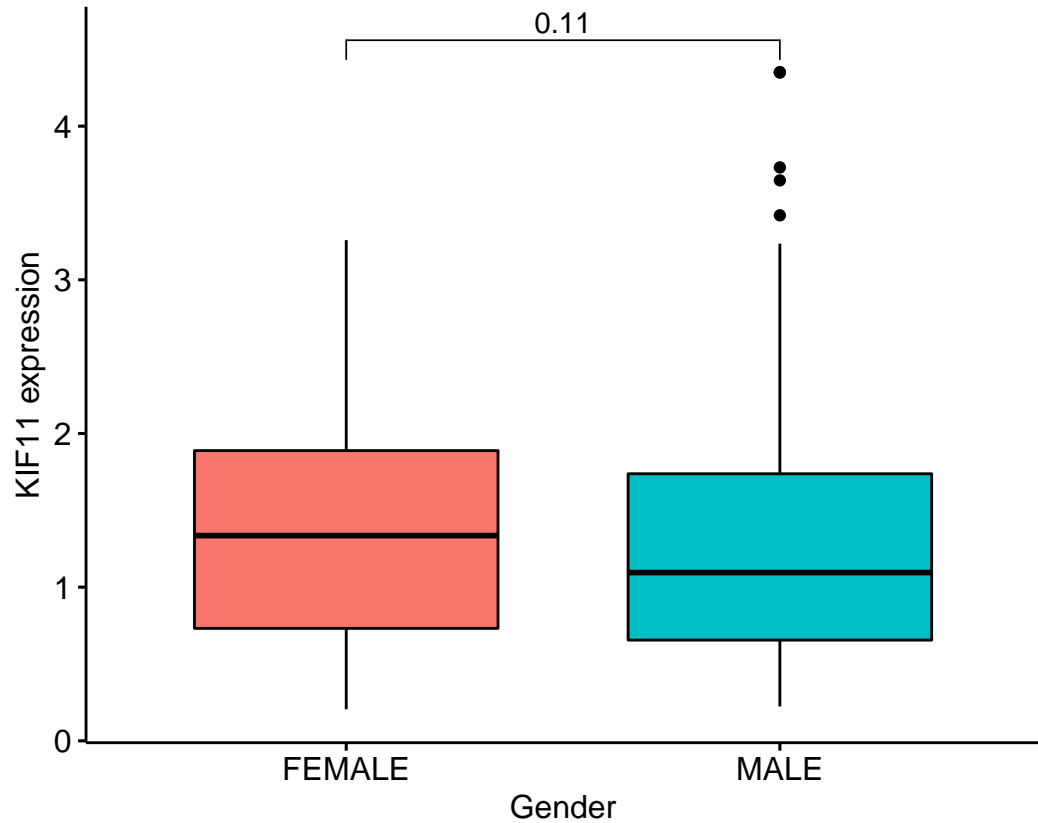

Supplement: Supplementary file 1 [file DataSheet1.zip › KIF11/Gender.pdf]

Grade G1 G2 G3 G4

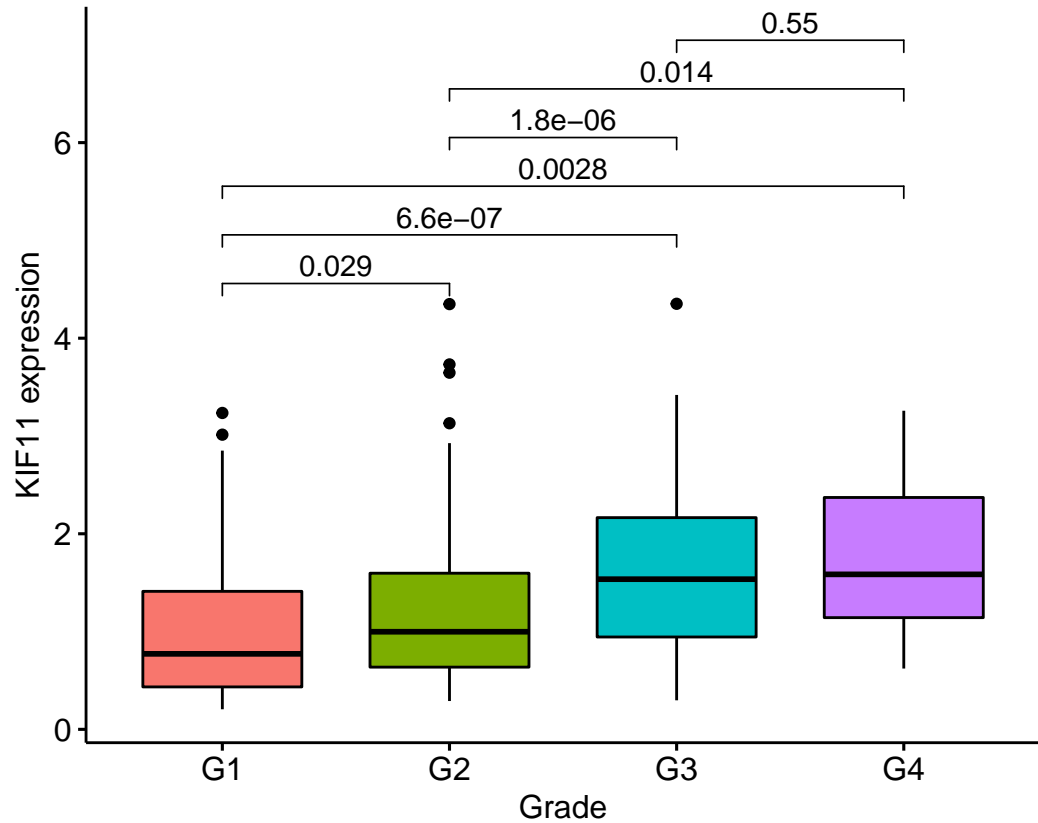

Supplement: Supplementary file 1 [file DataSheet1.zip › KIF11/Grade.pdf]

M M0 M1

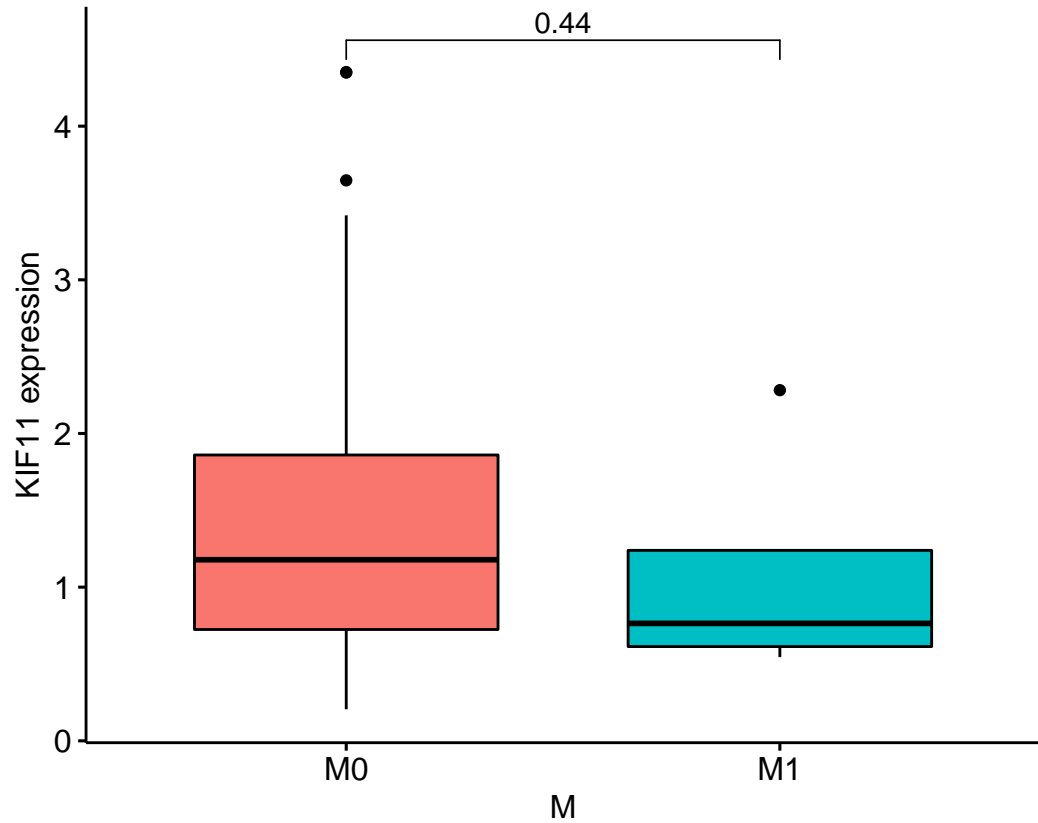

Supplement: Supplementary file 1 [file DataSheet1.zip › KIF11/M.pdf]

N 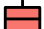 N0 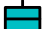 N1

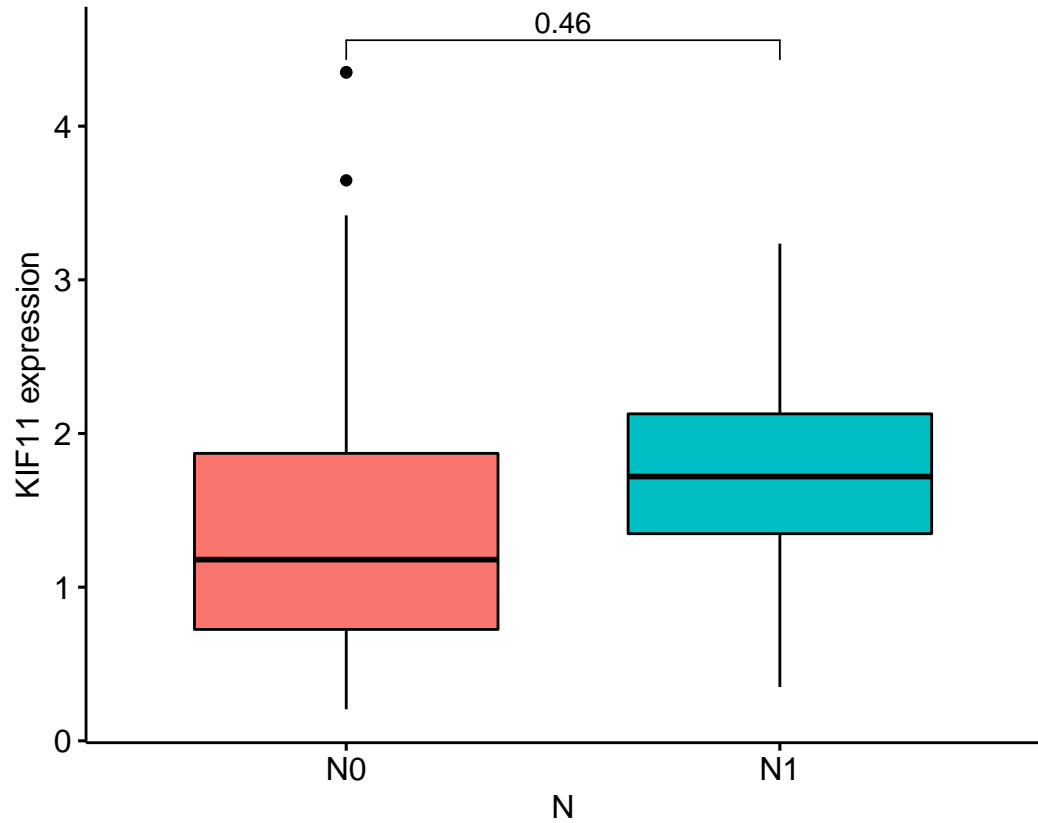

Supplement: Supplementary file 1 [file DataSheet1.zip › KIF11/N.pdf]

Stage I Stage II Stage III Stage IV

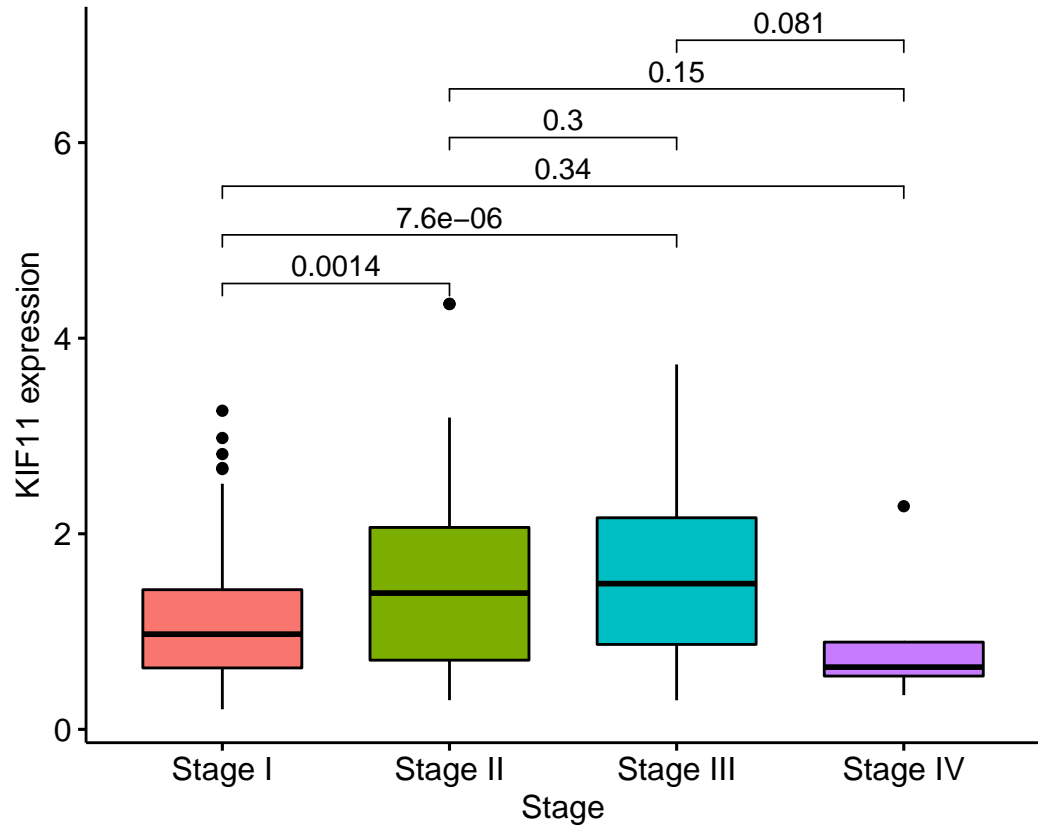

Supplement: Supplementary file 1 [file DataSheet1.zip › KIF11/Stage.pdf]

T 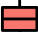 T1 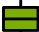 T2 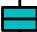 T3 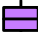 T4

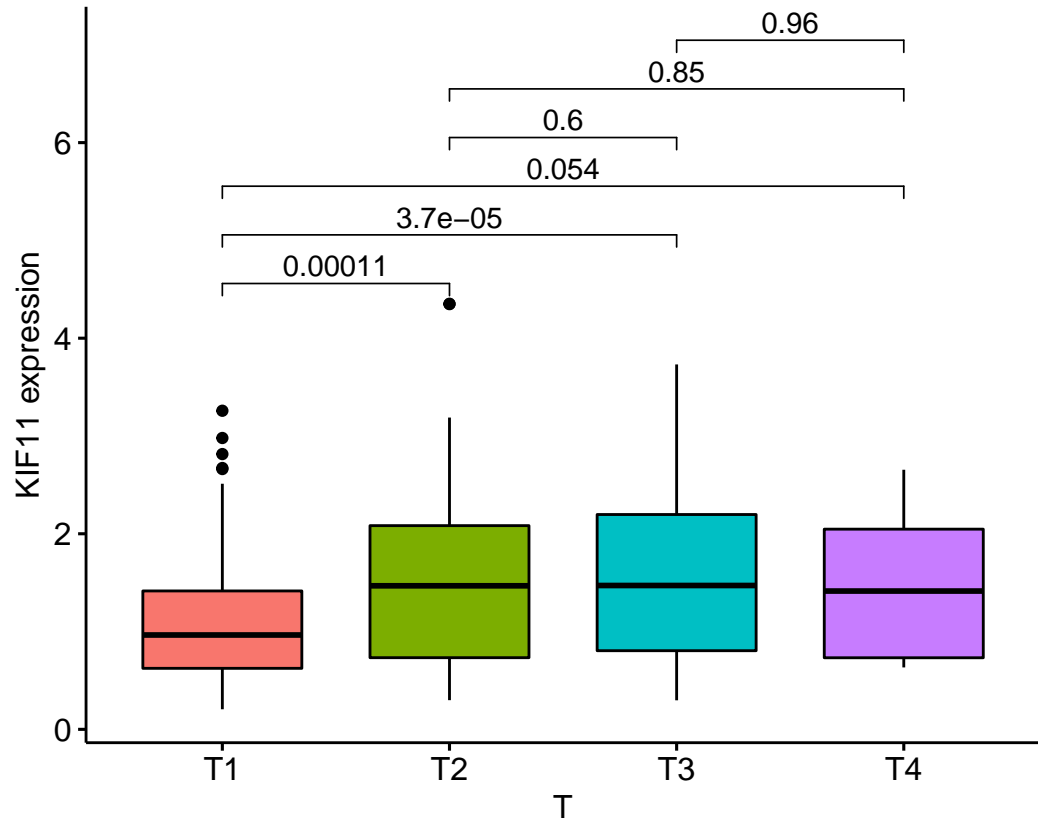

Supplement: Supplementary file 1 [file DataSheet1.zip › KIF11/T.pdf]

Age 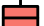 <=65 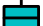 >65

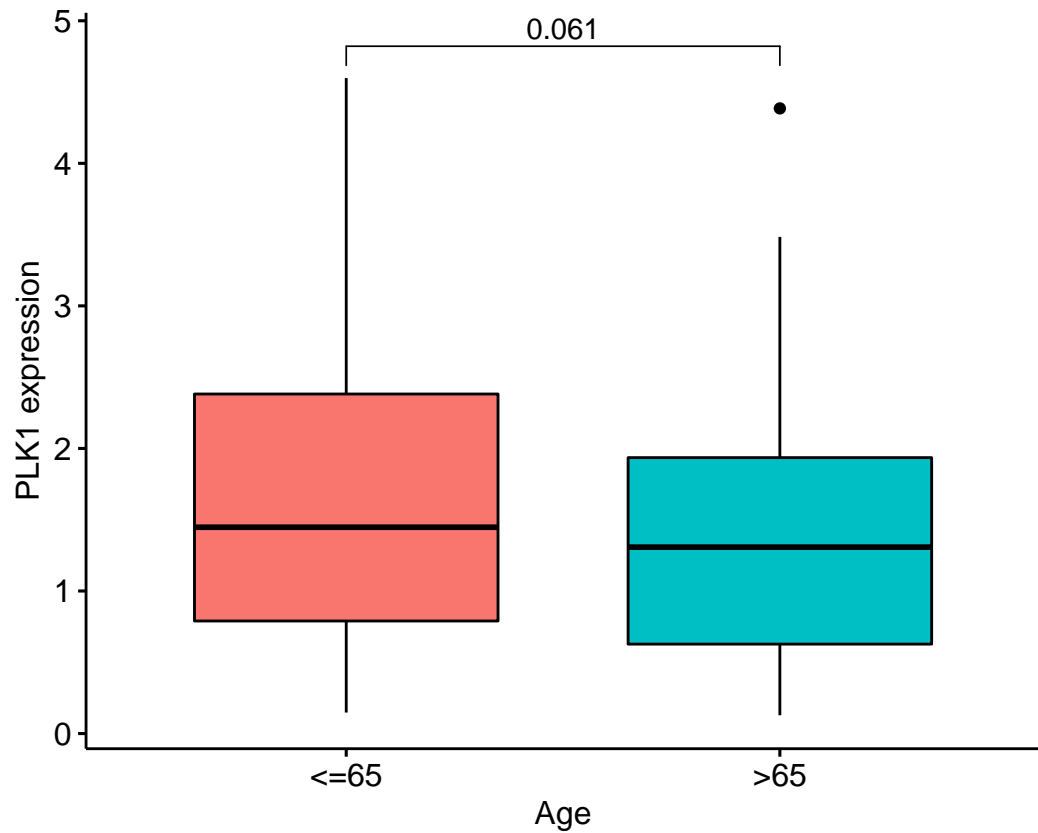

Supplement: Supplementary file 1 [file DataSheet1.zip › PLK1/Age.pdf]

Gender 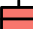 FEMALE 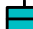 MALE

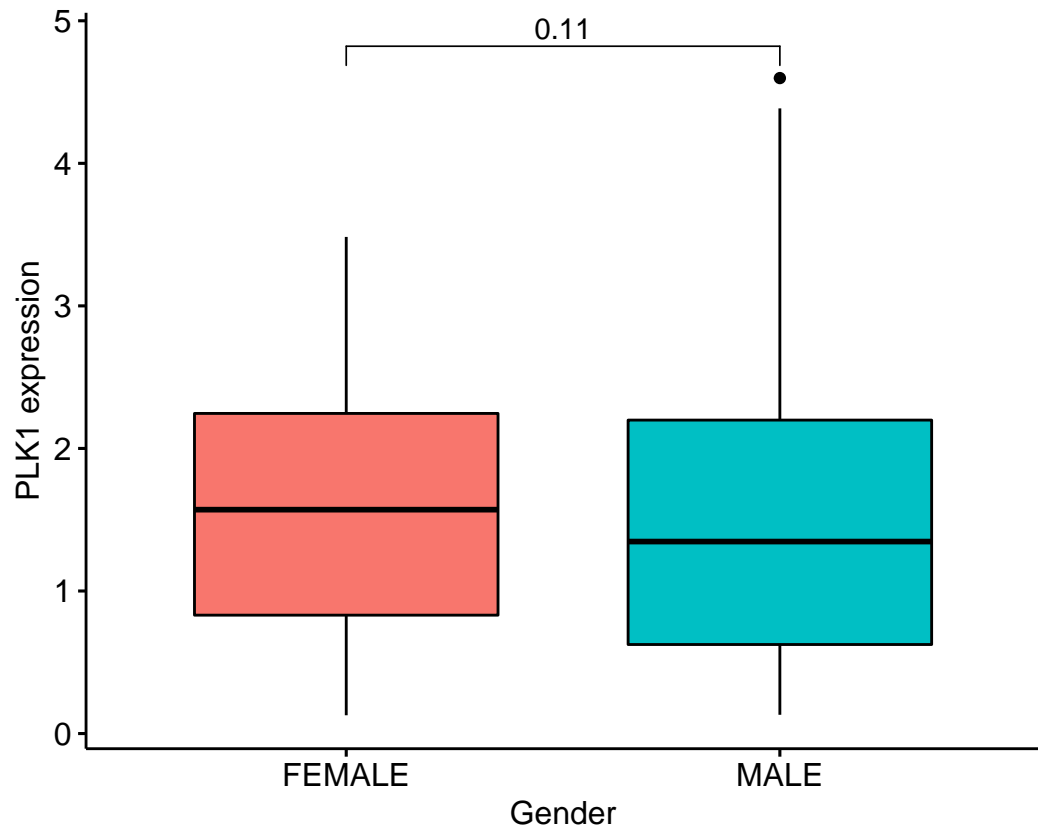

Supplement: Supplementary file 1 [file DataSheet1.zip › PLK1/Gender.pdf]

Grade G1 G2 G3 G4

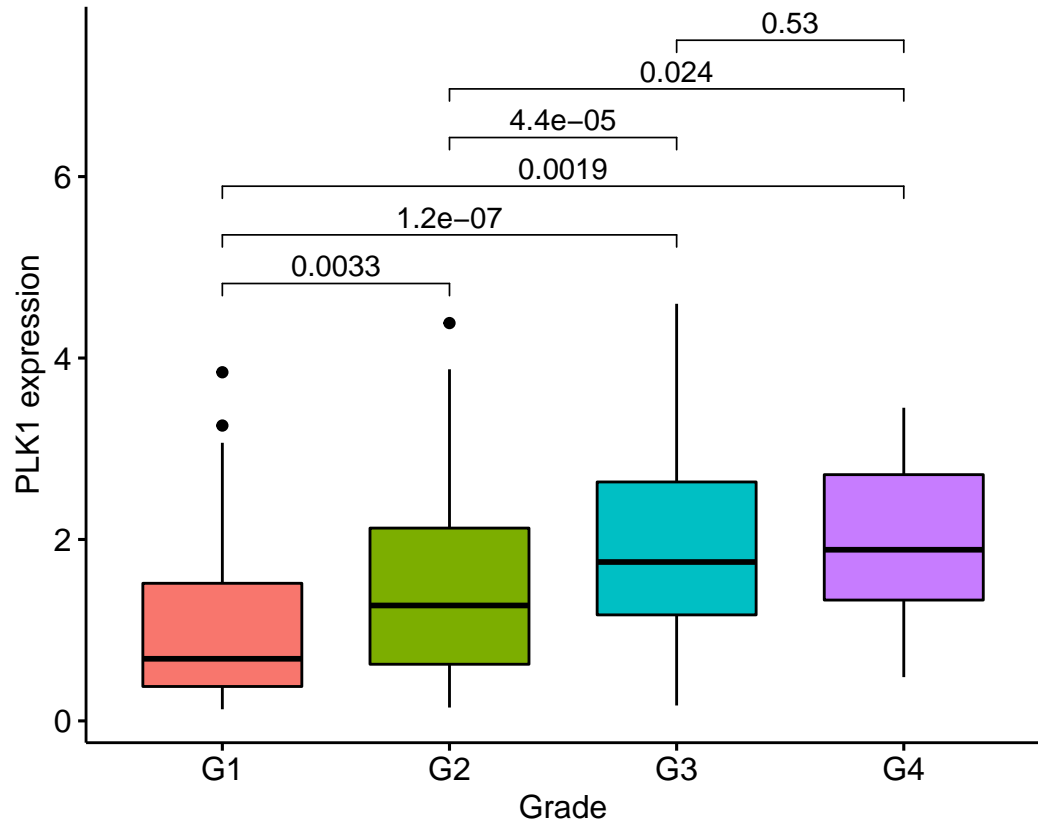

Supplement: Supplementary file 1 [file DataSheet1.zip › PLK1/Grade.pdf]

M M0 M1

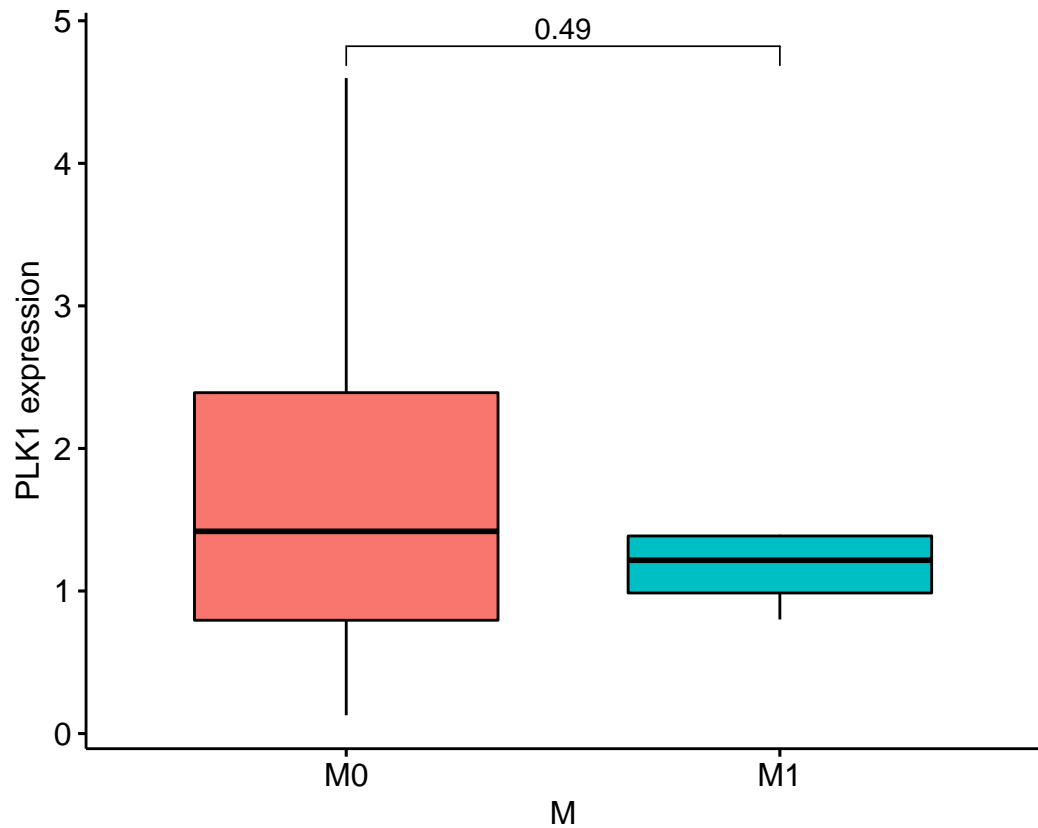

Supplement: Supplementary file 1 [file DataSheet1.zip › PLK1/M.pdf]

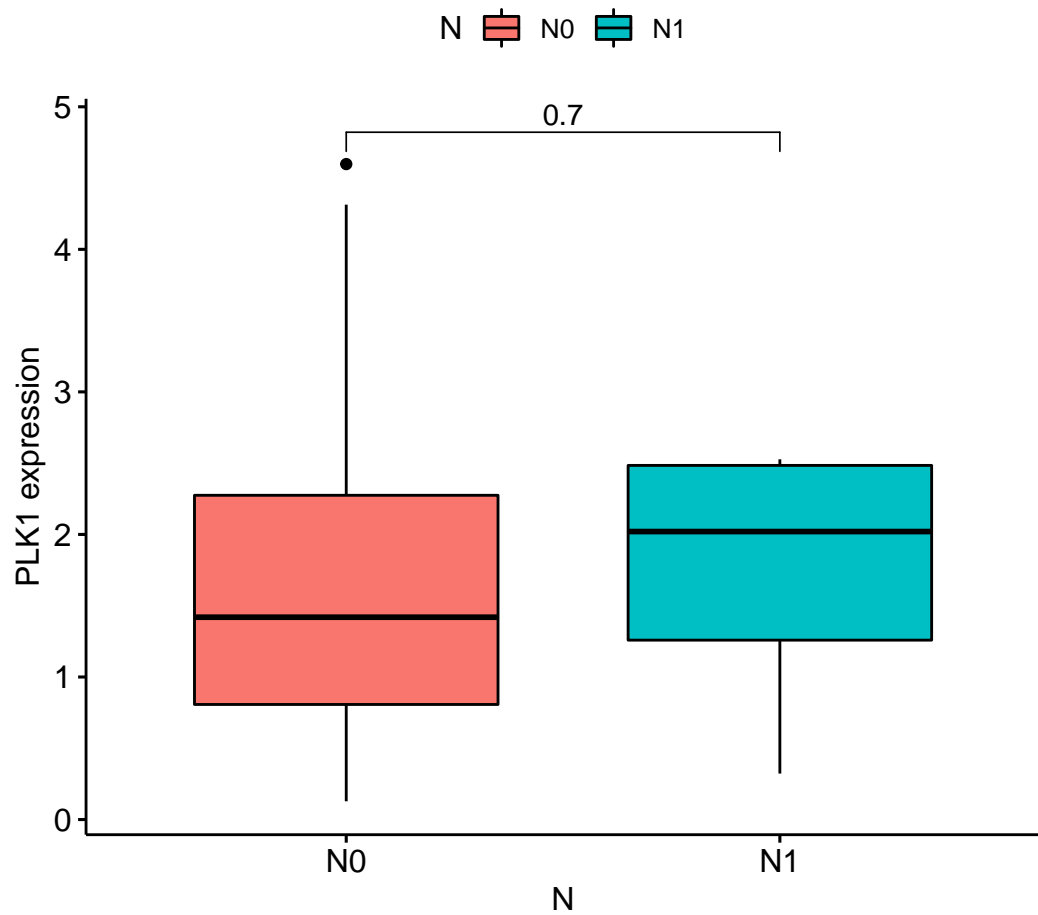

Supplement: Supplementary file 1 [file DataSheet1.zip › PLK1/N.pdf]

Stage I Stage II Stage III Stage IV

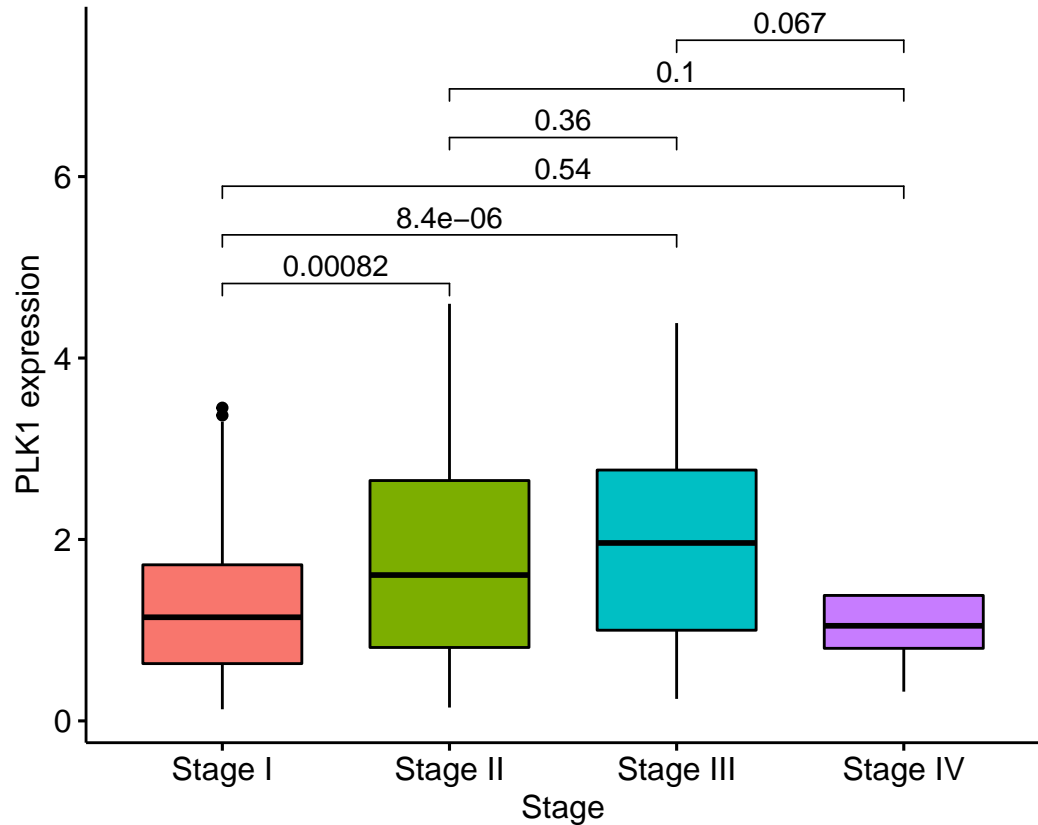

Supplement: Supplementary file 1 [file DataSheet1.zip › PLK1/Stage.pdf]

T T1 T2 T3 T4

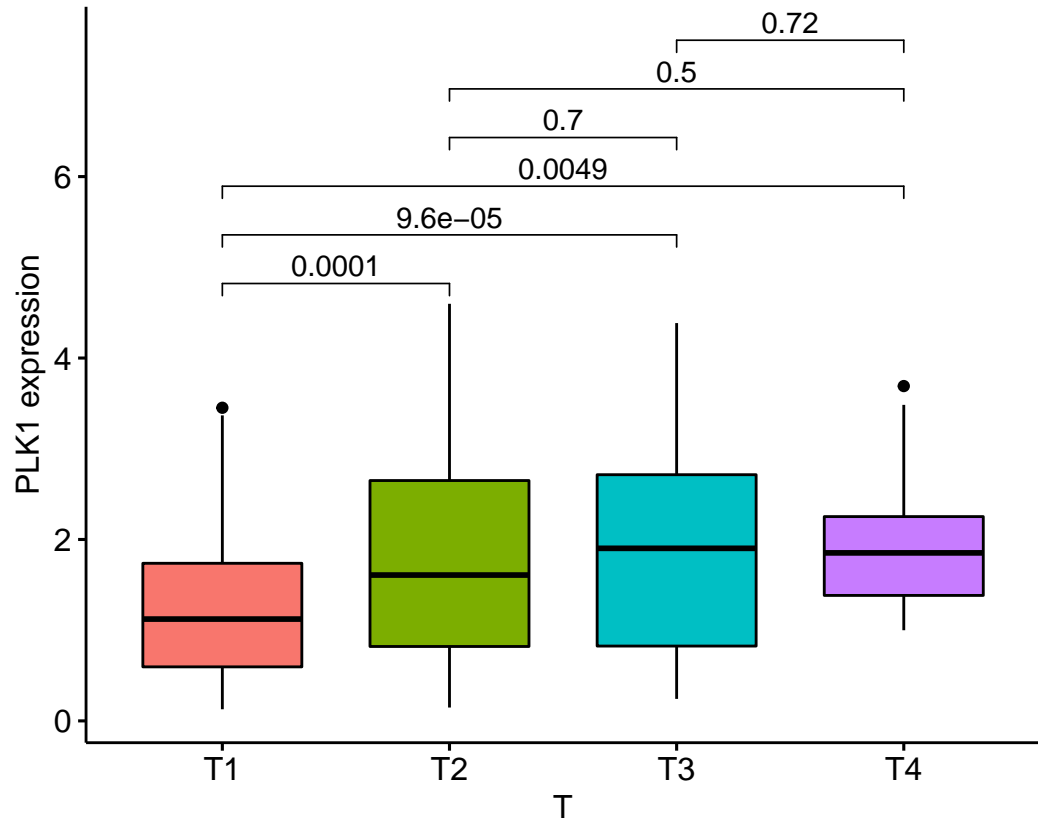

Supplement: Supplementary file 1 [file DataSheet1.zip › PLK1/T.pdf]

Age 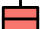 <=65 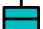 >65

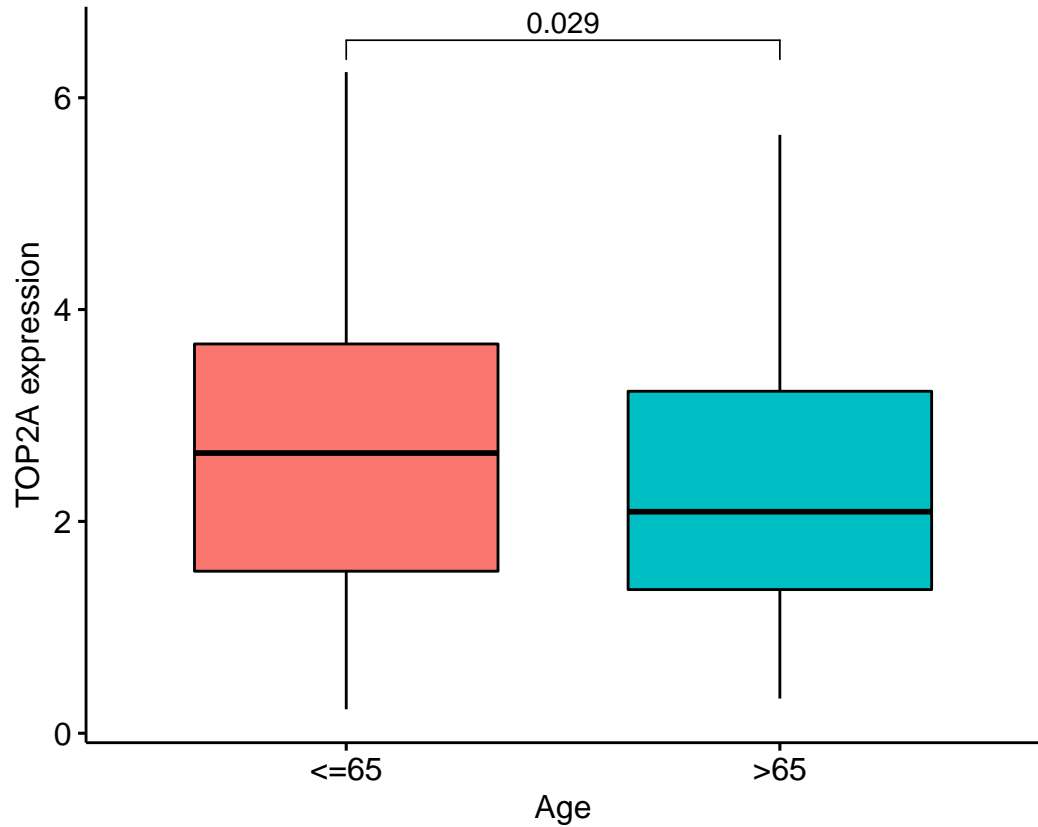

Supplement: Supplementary file 1 [file DataSheet1.zip › TOP2A/Age.pdf]

Gender 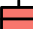 FEMALE 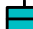 MALE

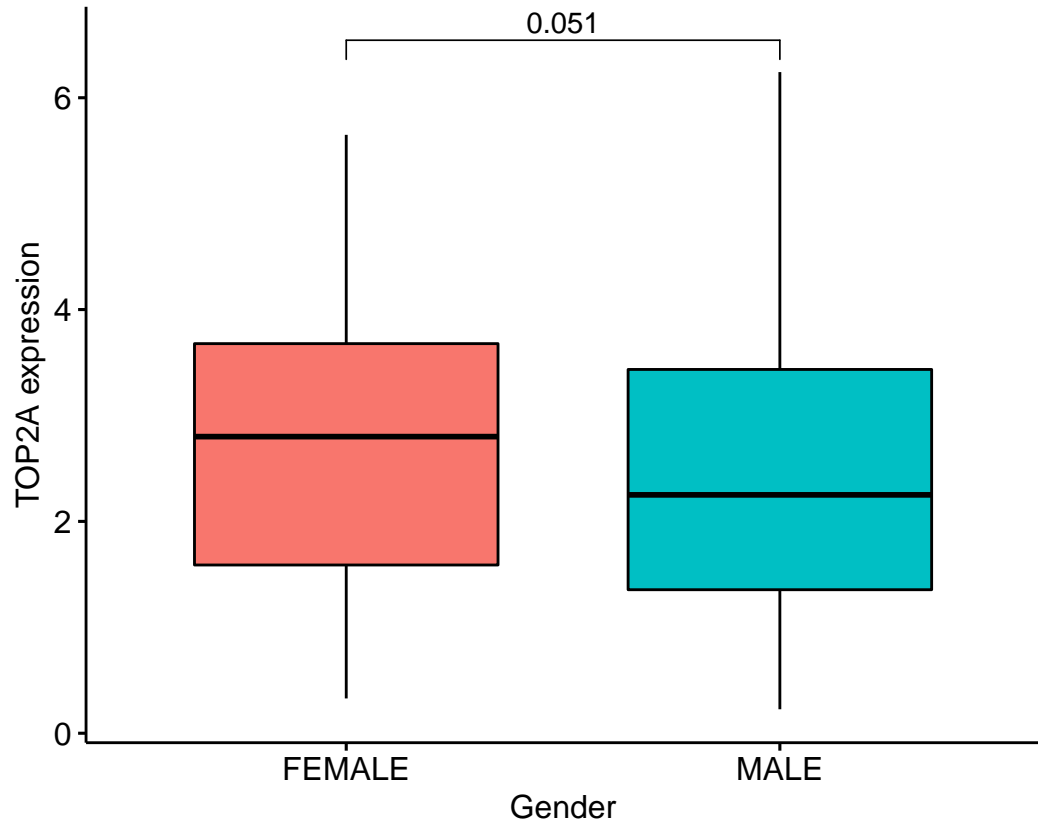

Supplement: Supplementary file 1 [file DataSheet1.zip › TOP2A/Gender.pdf]

Grade G1 G2 G3 G4

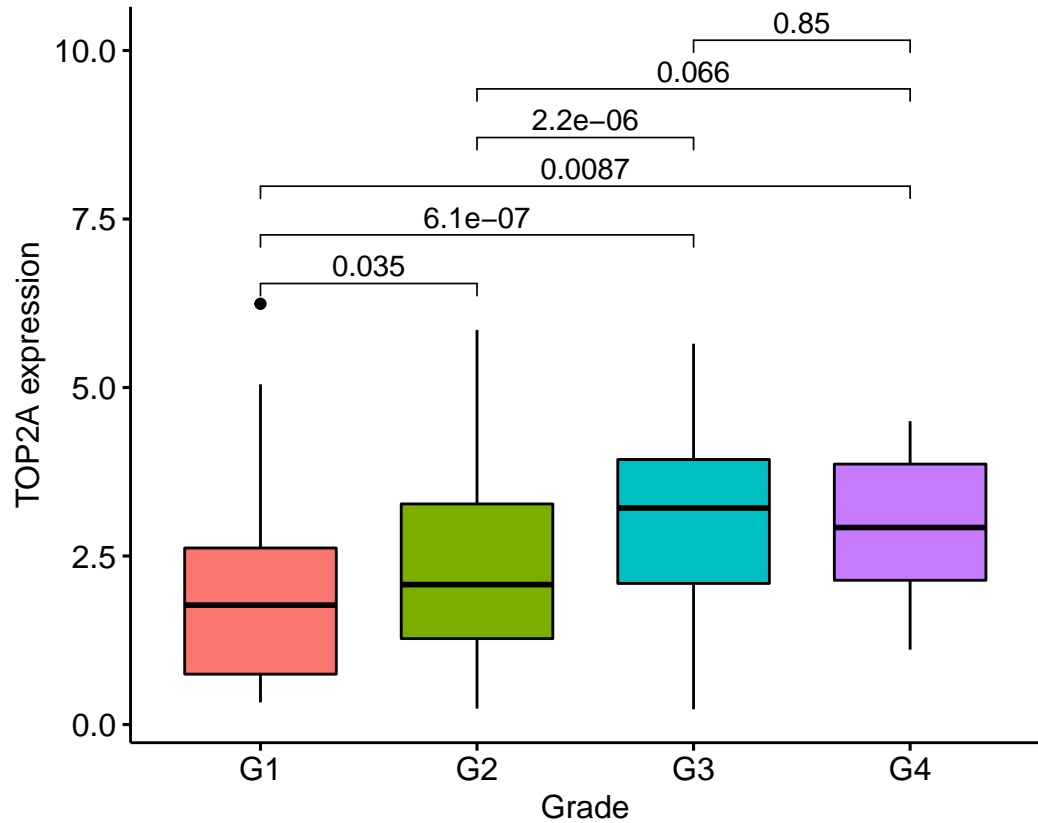

Supplement: Supplementary file 1 [file DataSheet1.zip › TOP2A/Grade.pdf]

M M0 M1

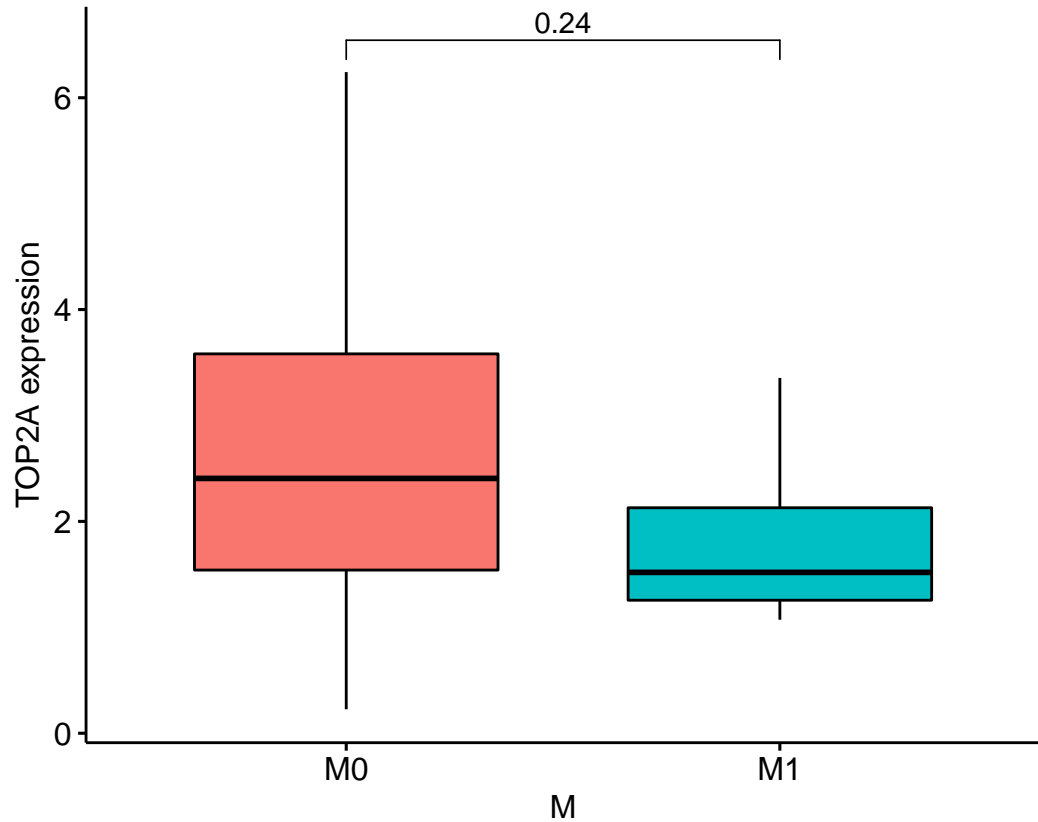

Supplement: Supplementary file 1 [file DataSheet1.zip › TOP2A/M.pdf]

TOP2A expression

N 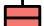 N0 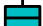 N1

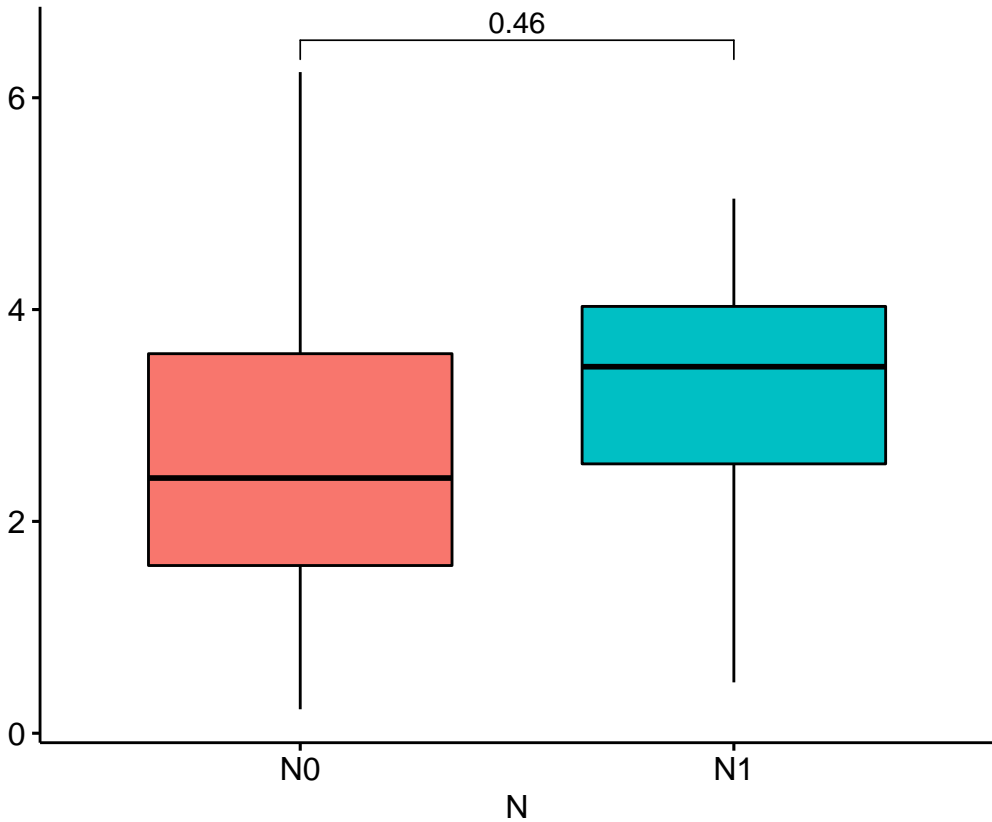

Supplement: Supplementary file 1 [file DataSheet1.zip › TOP2A/N.pdf]

Stage 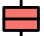 Stage I 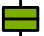 Stage II 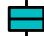 Stage III 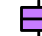 Stage IV

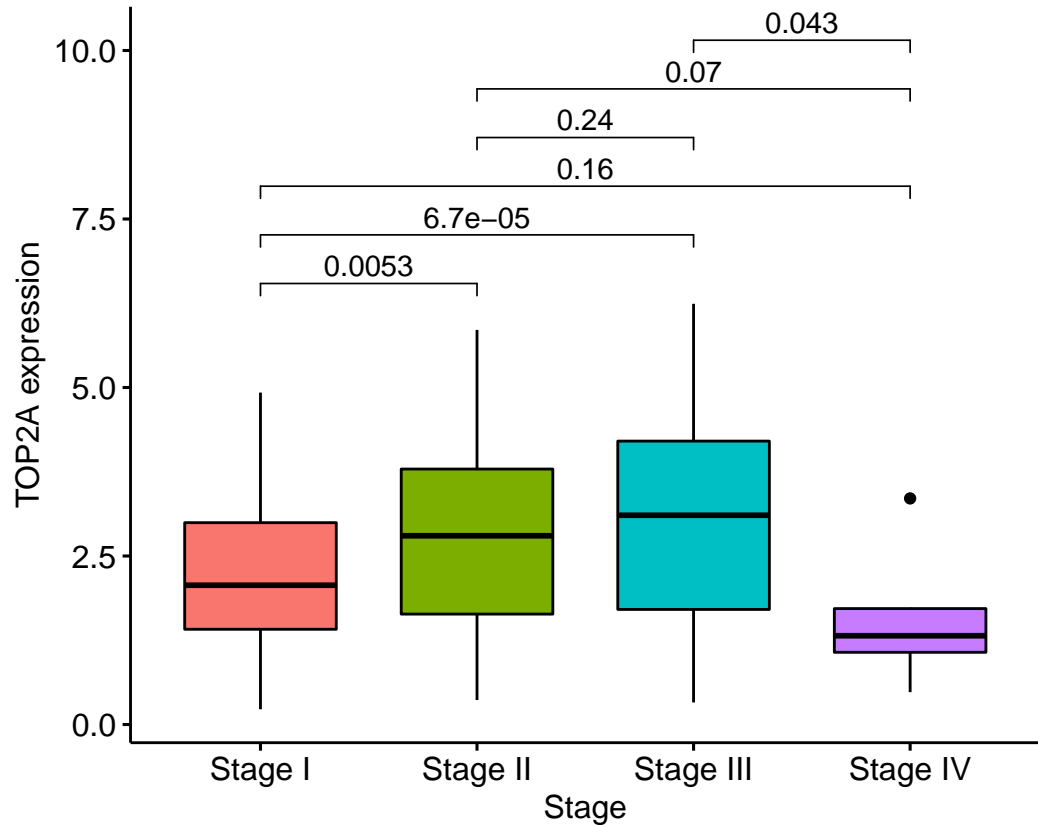

Supplement: Supplementary file 1 [file DataSheet1.zip › TOP2A/Stage.pdf]

T T1 T2 T3 T4

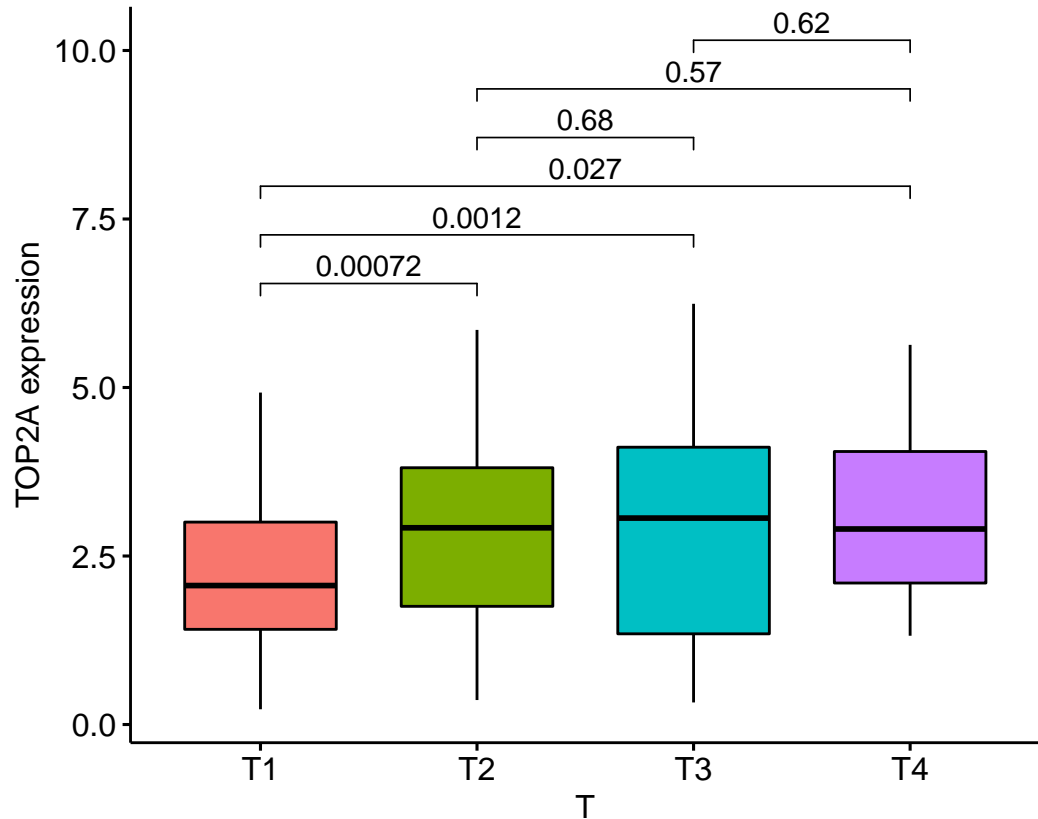

Supplement: Supplementary file 1 [file DataSheet1.zip › TOP2A/T.pdf]

ASPM low high

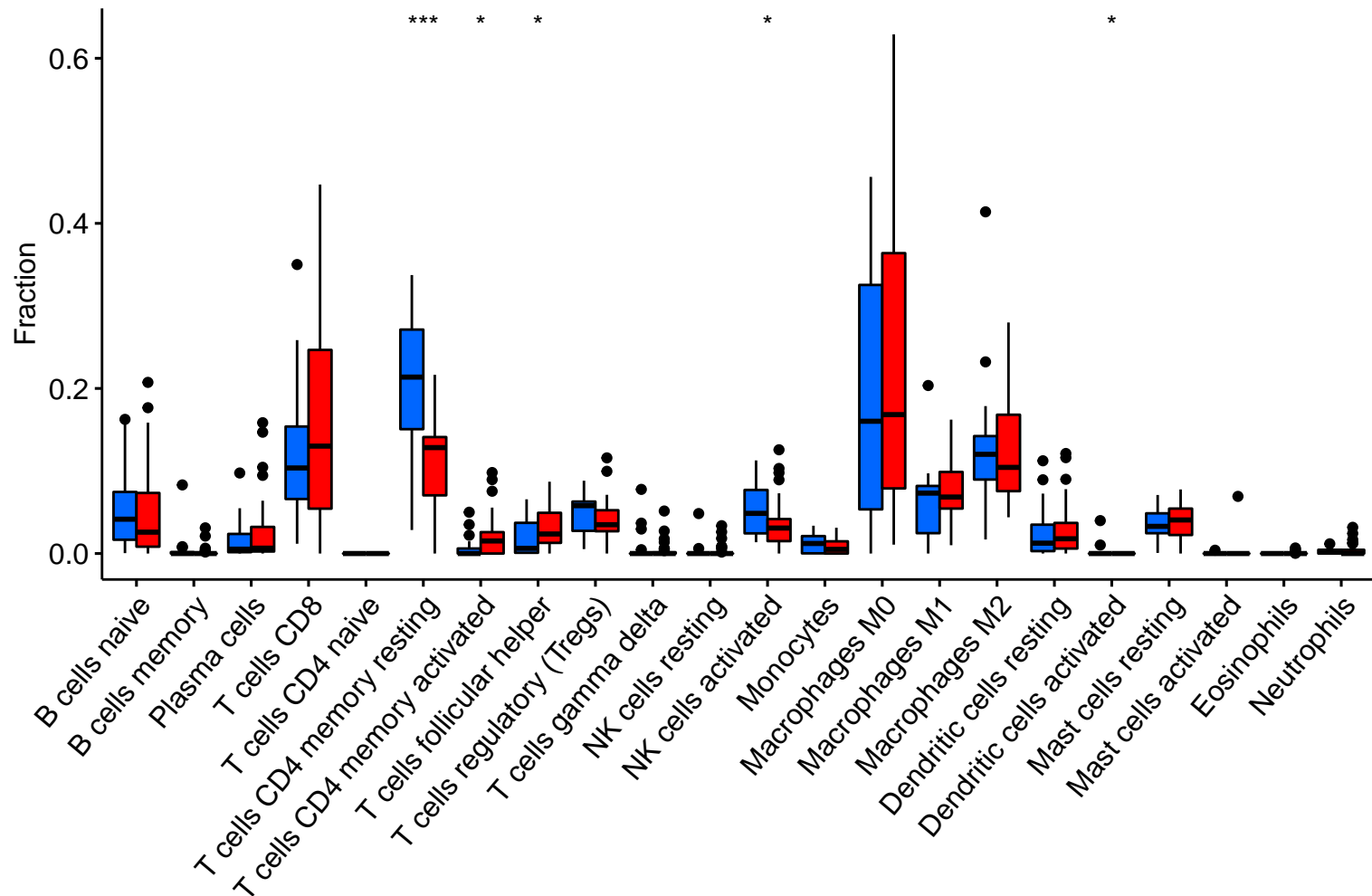

Supplement: Supplementary file 2 [file DataSheet2.zip › ASPM.pdf]

AURKB low high

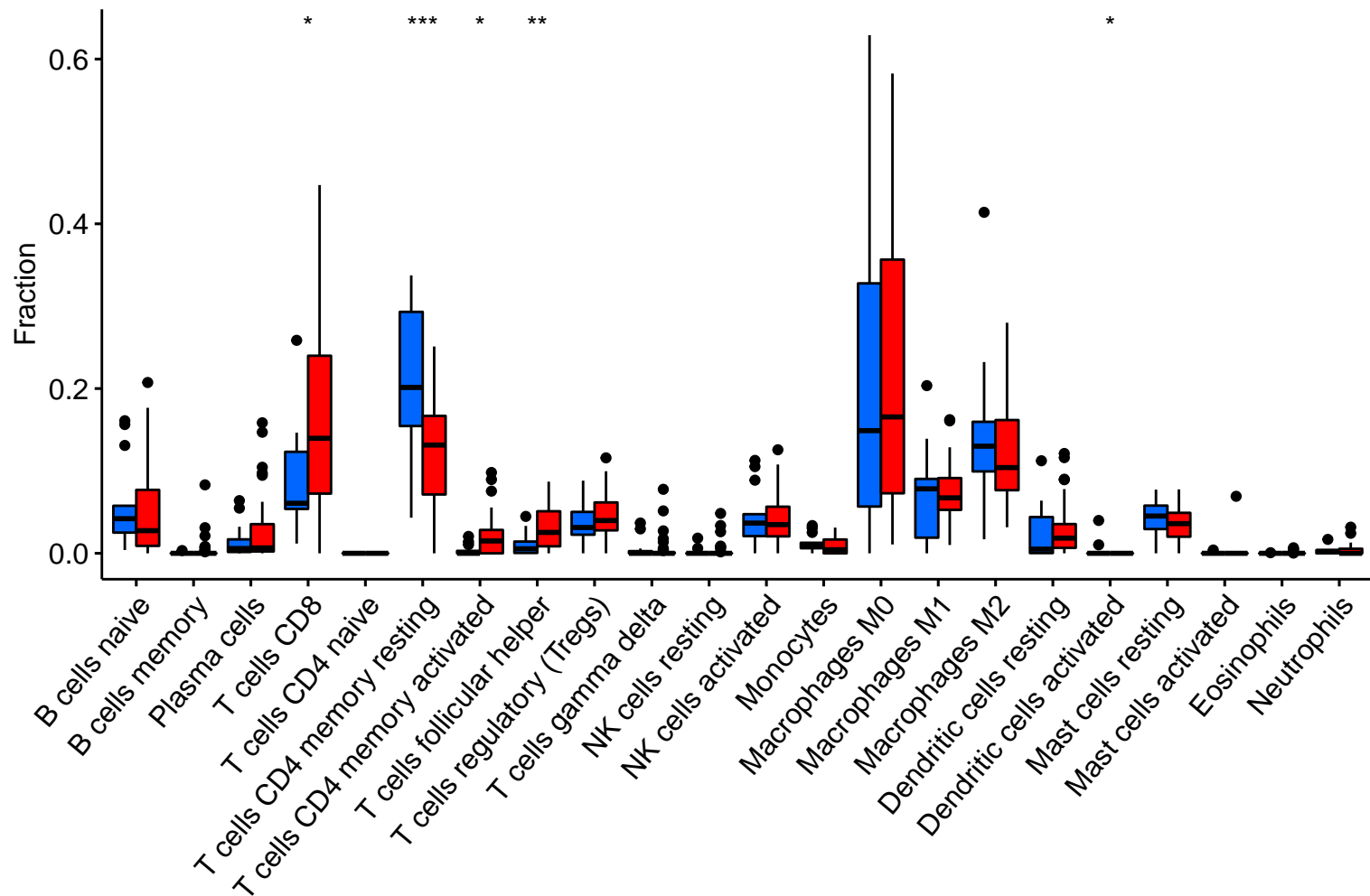

Supplement: Supplementary file 2 [file DataSheet2.zip › AURKB.pdf]

BUB1 low high

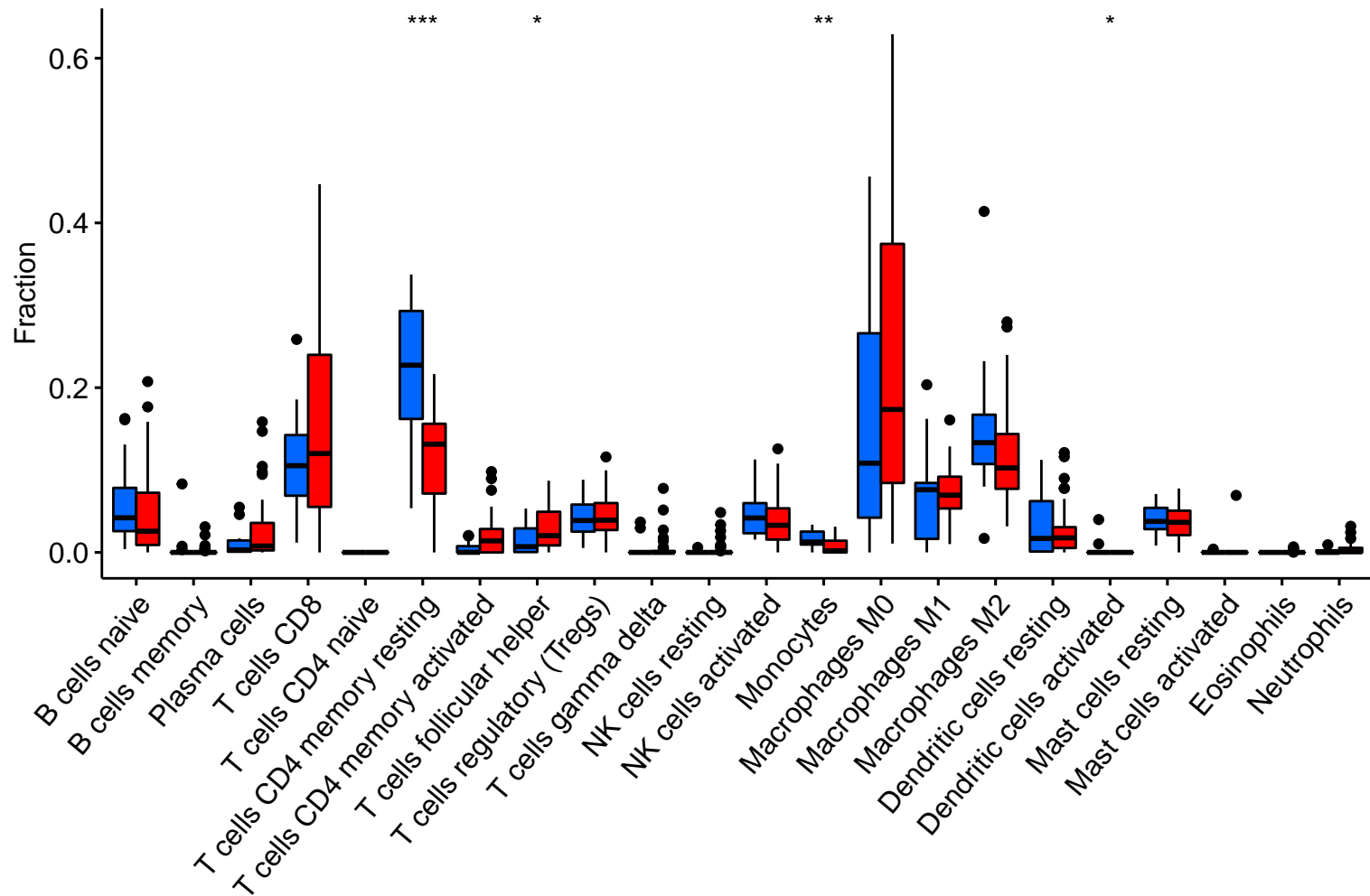

Supplement: Supplementary file 2 [file DataSheet2.zip › BUB1.pdf]

CCNB1 low high

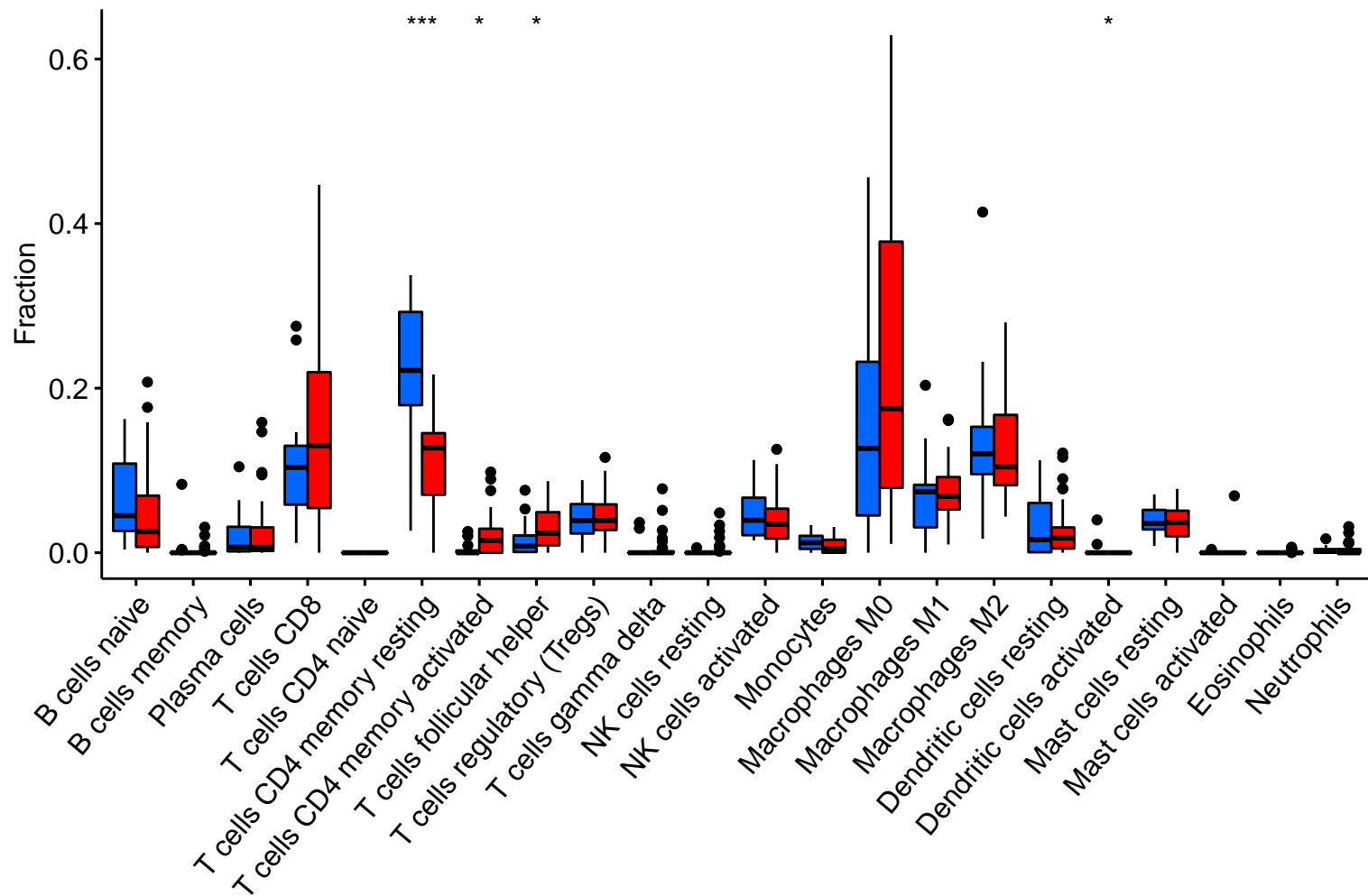

Supplement: Supplementary file 2 [file DataSheet2.zip › CCNB1.pdf]

CCNB2 low high

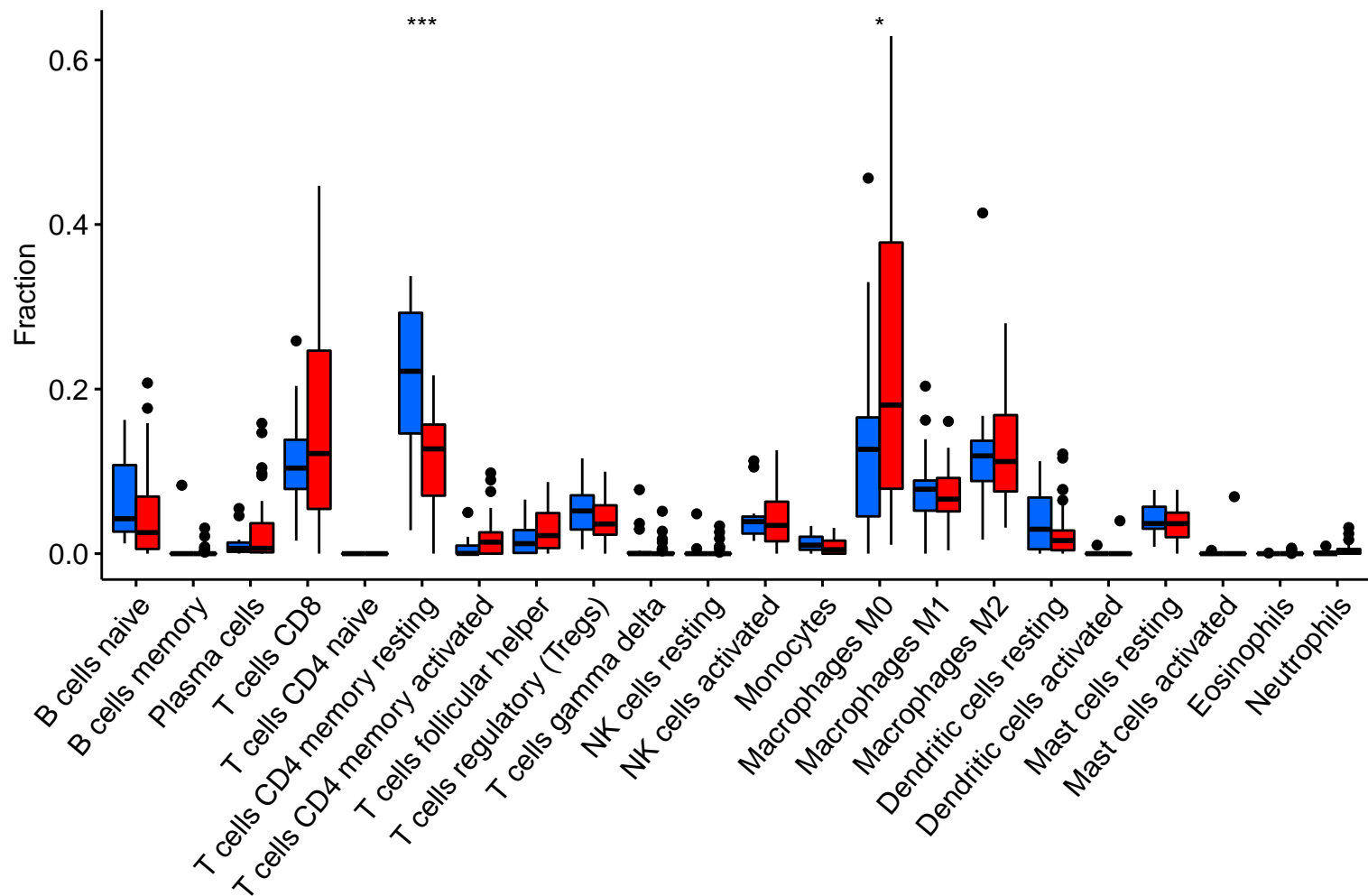

Supplement: Supplementary file 2 [file DataSheet2.zip › CCNB2.pdf]

CDC20 low high

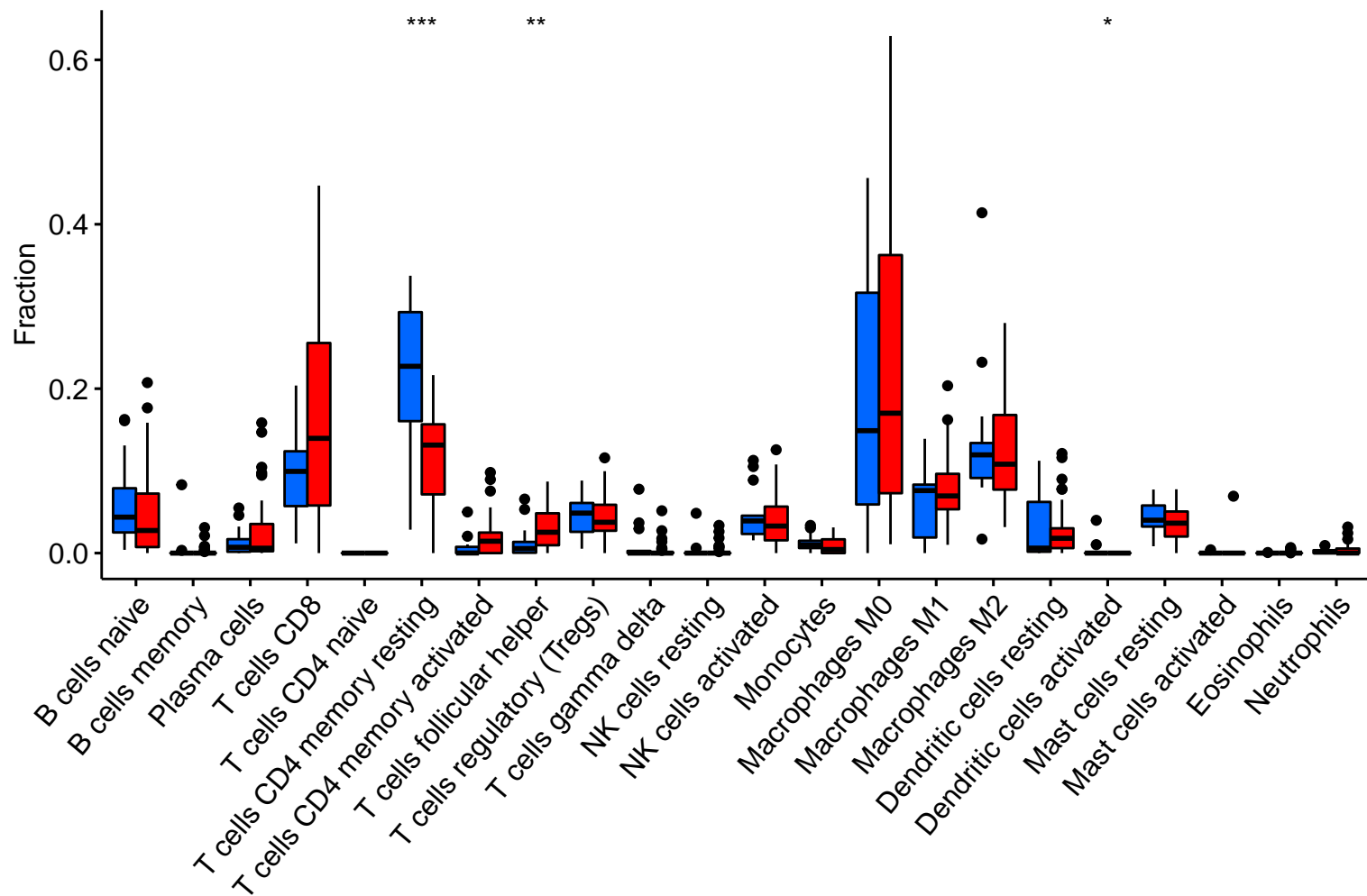

Supplement: Supplementary file 2 [file DataSheet2.zip › CDC20.pdf]

CDK1 low high

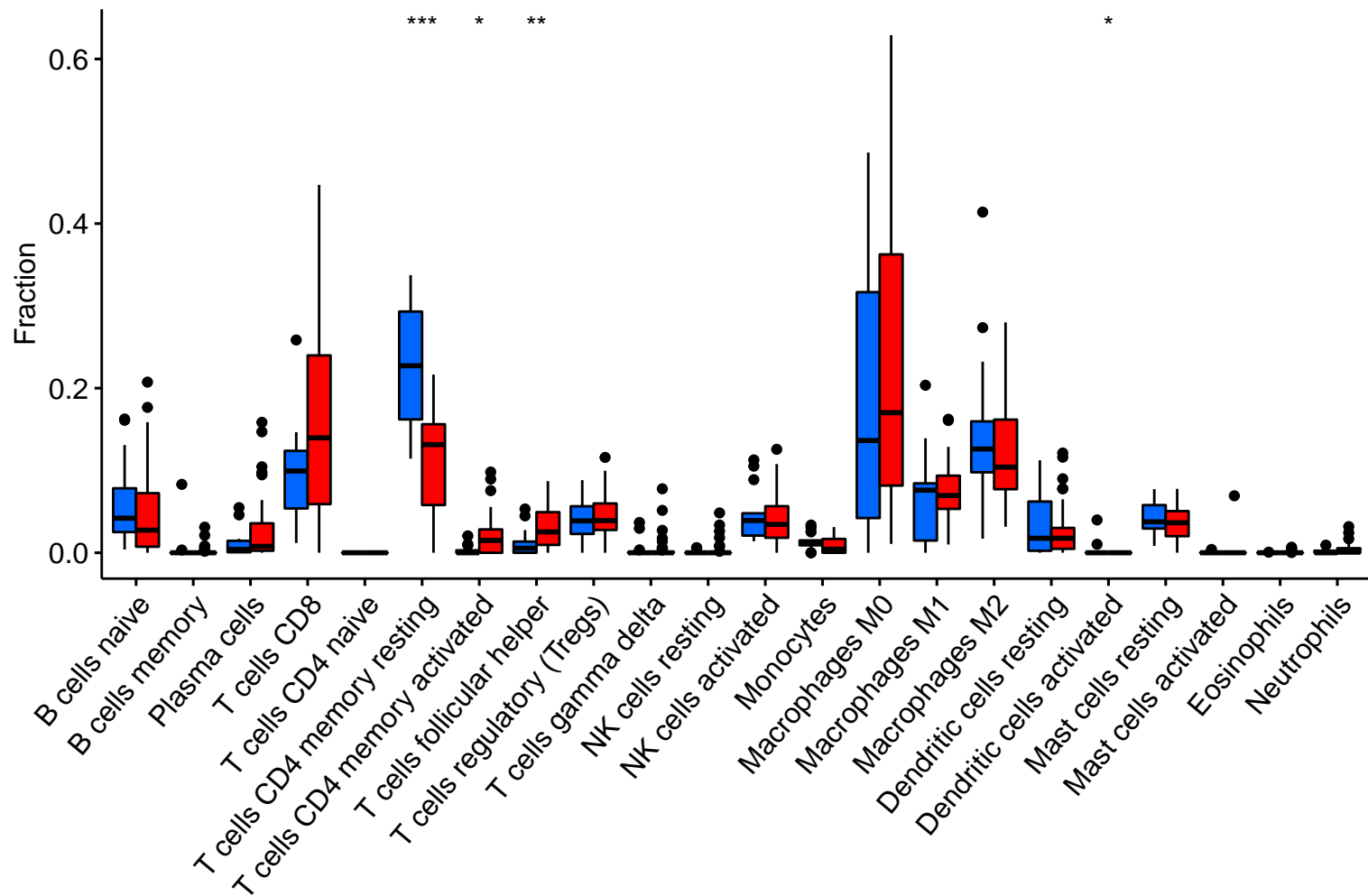

Supplement: Supplementary file 2 [file DataSheet2.zip › CDK1.pdf]

PLK1 low high

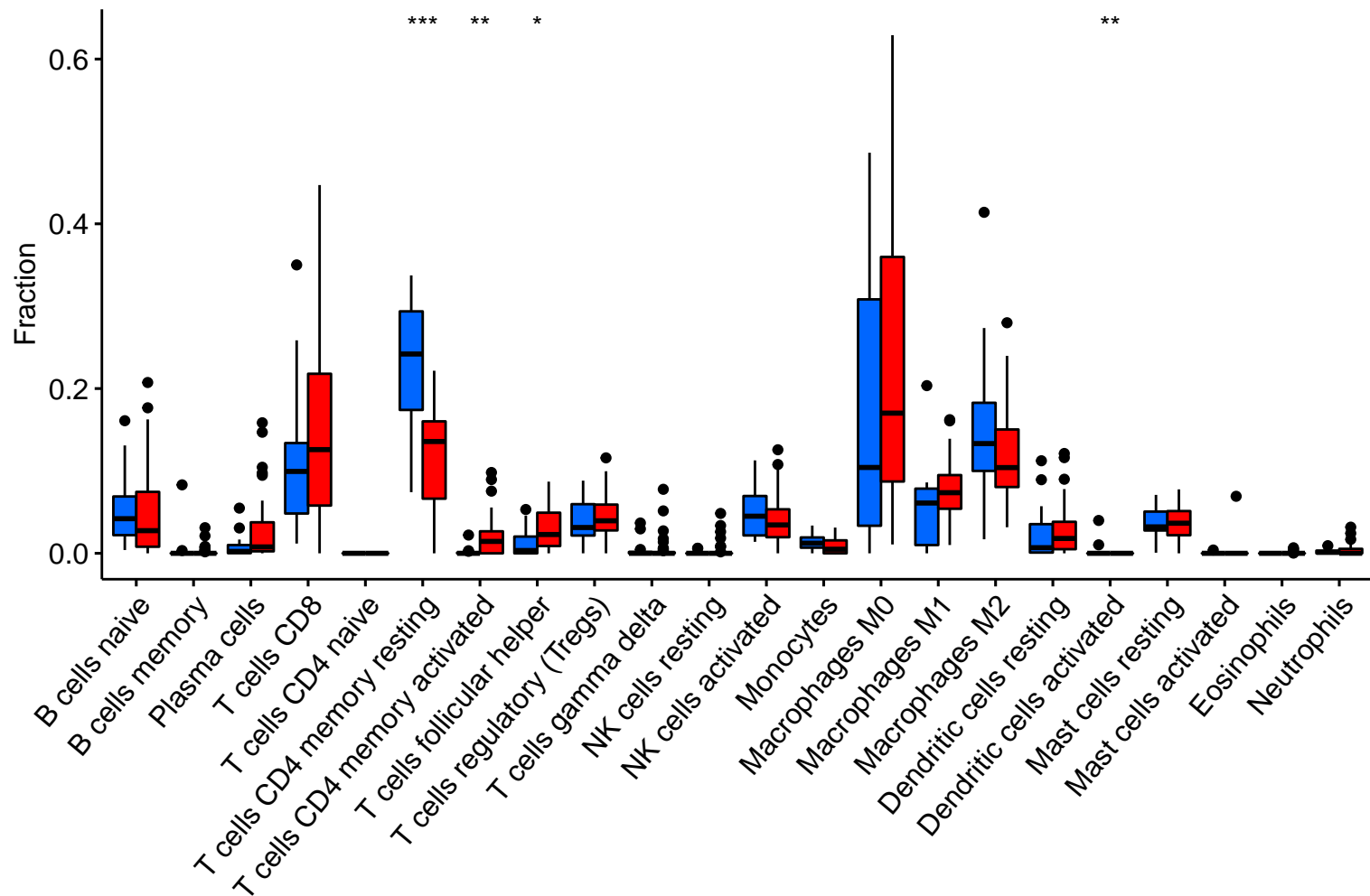

Supplement: Supplementary file 2 [file DataSheet2.zip › PLK1.pdf]

TOP2A 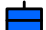 low 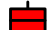 high

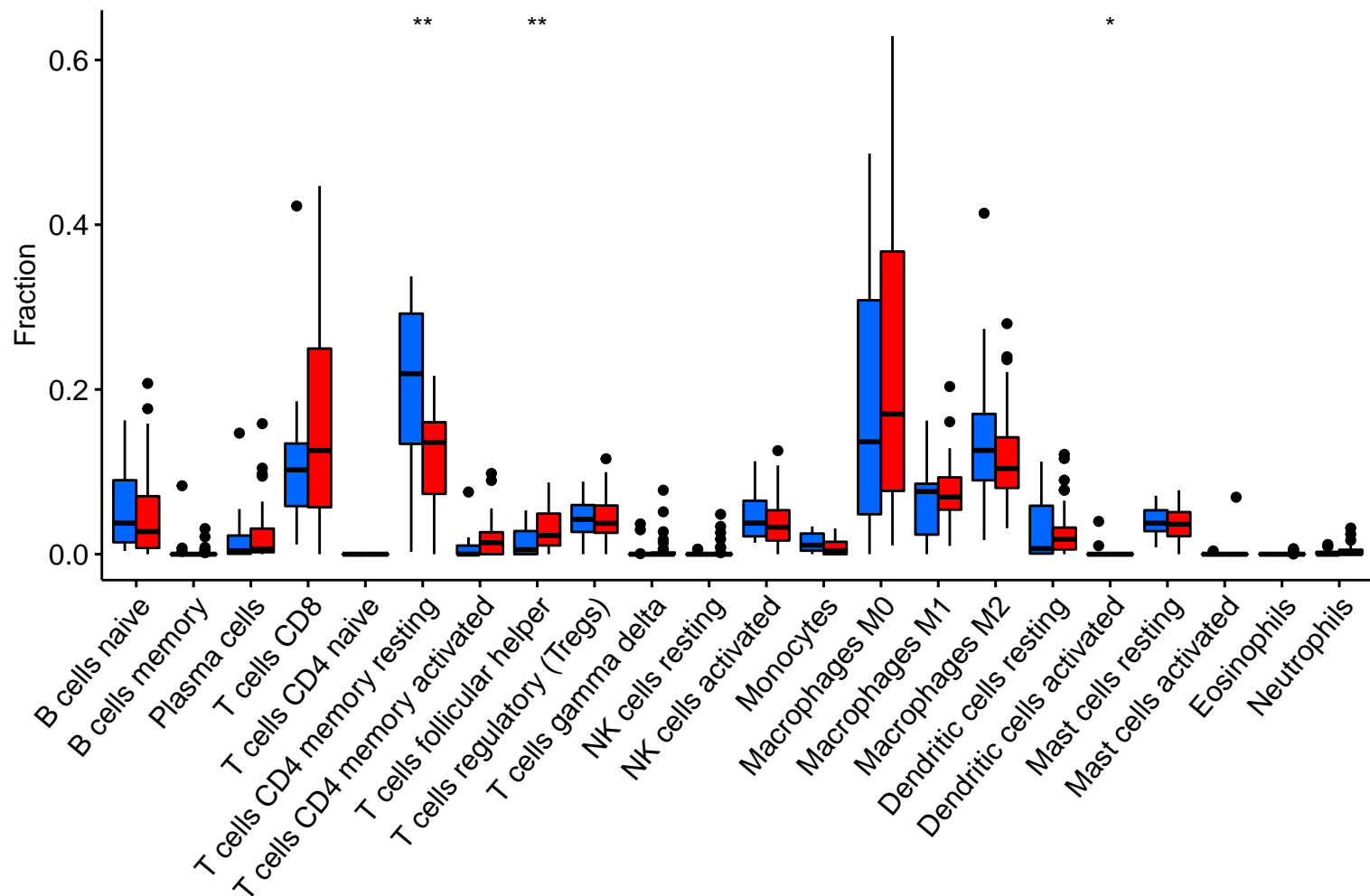

Supplement: Supplementary file 2 [file DataSheet2.zip › TOP2A.pdf]

KIF11 low high

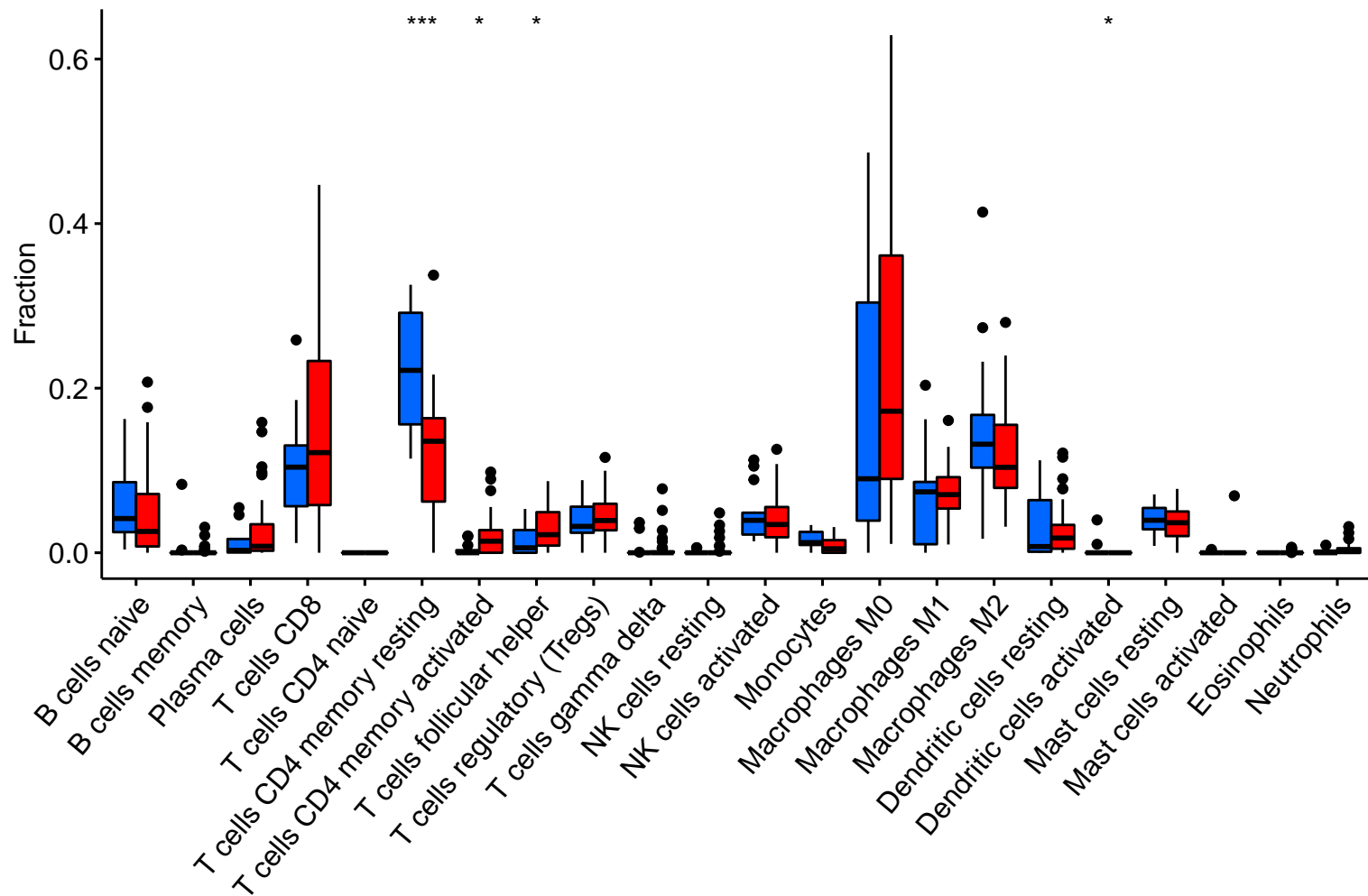

Supplement: Supplementary file 2 [file DataSheet2.zip › immune.diff.pdf]
